# Discovery of novel thiazolyl-pyrazolines as dual EGFR and VEGFR-2 inhibitors endowed with *in vitro* antitumor activity towards non-small lung cancer

**DOI:** 10.1080/14756366.2022.2104841

**Published:** 2022-08-23

**Authors:** Esraa A. Abdelsalam, Amer Ali Abd El-Hafeez, Wagdy M. Eldehna, Mahmoud A. El Hassab, Hala Mohamed M. Marzouk, Mahmoud M. Elaasser, Nageh A. Abou Taleb, Kamilia M. Amin, Hatem A. Abdel-Aziz, Pradipta Ghosh, Sherif F. Hammad

**Affiliations:** a Department of Pharmaceutical Chemistry, Faculty of Pharmacy, Helwan University, Cairo, Egypt; b Department of Cellular and Molecular Medicine, University of California San Diego, La Jolla, CA, USA; c Pharmacology and Experimental Oncology Unit, Department of Cancer Biology, National Cancer Institute, Cairo University, Cairo, Egypt; d Department of Pharmaceutical Chemistry, Faculty of Pharmacy, Kafrelsheikh University, Kafrelsheikh, Egypt; e School of Biotechnology, Badr University in Cairo, Badr City, Cairo, Egypt; f Department of Medicinal Chemistry, Faculty of Pharmacy, King Salman International University (KSIU), South Sinai, Egypt; g Department of Biochemistry, Faculty of Medicine, Minia University, El-Minia, Egypt; h The Regional Center for Mycology and Biotechnology, Al-Azhar University, Cairo, Egypt; i Department of Pharmaceutical Chemistry, Faculty of Pharmacy, Cairo University, Cairo, Egypt; j Department of Applied Organic Chemistry, National Research Centre, Dokki, Giza, Egypt; k Department of Medicine, University of California San Diego, La Jolla, CA, USA; l Moores Comprehensive Cancer Center, University of California San Diego, La Jolla, CA, USA; m Veterans Affairs Medical Center, La Jolla, CA, USA; n PharmD Program and Basic and Applied Sciences Institute, Egypt-Japan University of Science and Technology (E-JUST), Alexandria, Egypt

**Keywords:** Anticancer, molecular docking, EGFR inhibitors, VEGFR-2 inhibitors, EGFR-mutated NSCLC, dual kinase inhibitors

## Abstract

New series of thiazolyl-pyrazoline derivatives (**7a–7d**, **10a–10d** and **13a–13f**) have been synthesised and assessed for their potential EGFR and VEGFR-2 inhibitory activities. Compounds **10b** and **10d** exerted potent and selective inhibitory activity towards the two receptor tyrosine kinases; EGFR (IC_50_ = 40.7 ± 1.0 and 32.5 ± 2.2 nM, respectively) and VEGFR-2 (IC_50_ = 78.4 ± 1.5 and 43.0 ± 2.4 nM, respectively). The best anti-proliferative activity for the examined thiazolyl-pyrazolines was observed against the non-small lung cancer cells (NSCLC). Compounds **10b** and **10d** displayed pronounced efficacy against A549 (IC_50_ = 4.2 and 2.9 µM, respectively) and H441 cell lines (IC_50_ = 4.8 and 3.8 µM, respectively). Moreover, our results indicated that **10b** and **10d** were much more effective towards EGFR-mutated NSCLC cell lines (NCI-H1650 and NCI-H1975 cells) than gefitinib. Finally, compounds **10b** and **10d** induce G2/M cell cycle arrest and apoptosis and inhibit migration in A549 cancerous cells.

## Graphical Abstract

**Figure:**
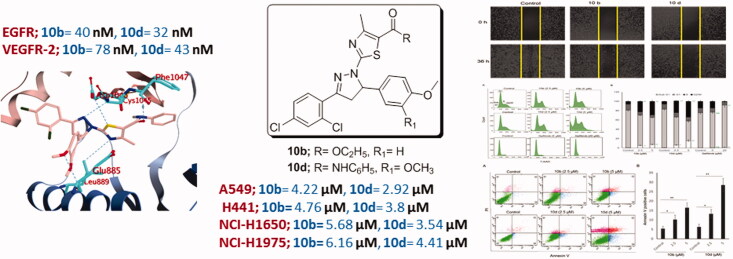

## 1. Introduction

Cancer remains as a major health problem and a life-threatening disease even after decades of fundamental biomedical advances leading to the development of a war chest of anti-tumour therapies. This is largely because of the ability of cancer cells to develop resistance against most antitumor agents^1^.

Lung cancer is the second most common cancer and the leading cause of cancer-related deaths in both men and women accounting for about 26% of all cancer deaths^2^. It has been shown that epidermal growth factor receptor (EGFR), the prototypical growth factor receptor tyrosine kinase (RTK)^3^, is one of the most important key players in the development of many lethal cancers globally^4^^,^^5^ including: colorectal cancers, ovarian cancers^6^, breast cancers^7^, and non-small-cell lung cancers (NSCLC) in which EGFR overexpression takes place in approximately 43–89% of all cases and relates with diminutive survival and chemoresistance^8^. Accordingly, interruption of the EGFR signalling pathway using small-molecule tyrosine kinase inhibitors (TKIs) is considered as a well-established therapeutic approach in cancer treatment since the approval of gefitinib by the United States Food and Drug Administration (FDA) in 2003^9^^,^^10^.

**Figure 1.**
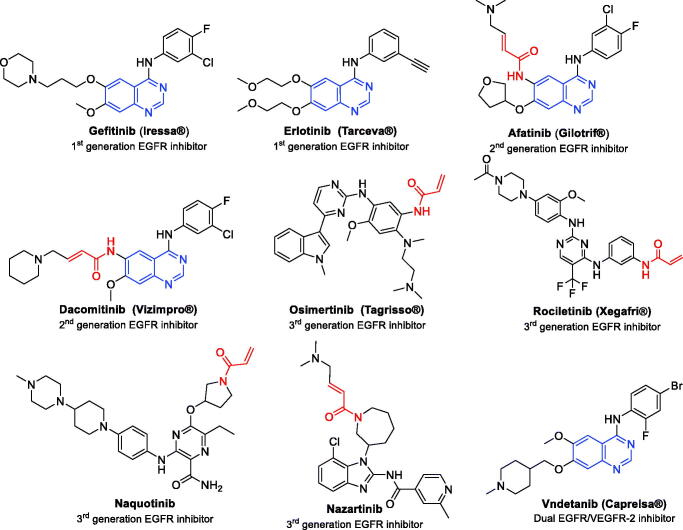
First-, second- and third generation EGFR inhibitors and dual EGFR/VEGFR-2 inhibitor.

Gefitinib and erlotinib (Figure 1) are first-generation EGFR inhibitors that have been approved for the treatment of advanced NSCLC with activating mutations in the EGFR tyrosine kinase domain mainly: L858R (EGFR^L858R^)^11–14^. However, some patients develop secondary drug resistance mutations which results in a relapse after nearly one year of treatment^15^. The “gatekeeper” mutation is the predominant one which is a single point substitution of Thr790 with Met in exon 20 (EGFR^T790M^)^16^. Hence, afatinib and dacomitinib (Figure 1) have been developed as irreversible second generation EGFR inhibitors^17^^,^^18^ because they have electrophilic Michael-acceptor systems, such as acrylamide moiety which forms a covalent bond with Cys797^19^. This resulted in blocking of T790M resistance mutation by increasing target residence time^20^.

Despite the promising *in vitro* results in patients with erlotinib-resistant cancers^21^, they showed poor efficacy at clinically achievable concentration^22^. This is due to the narrow therapeutic window, as the dose required for the inhibition of mutated EGFR^T790M^ resulted in severe side effects as rash and diarrhoea from inhibiting EGFR^wt^^23^. Recently, this problem was solved by the discovery of third generation EGFR inhibitors as osimertinib (Tagrisso®, AZD9291), rociletinib (CO-1686), naquotinib (ASP8273) and nazartinib (EGF816)^24^^,^^25^ (Figure 1), however, a resistance has been developed too^26–28^. So, exploring more efficient EGFR inhibitors is an urgent and critical demand to surmount the continual evolution of resistance to the current inhibitors.

Besides EGFR, there are other RTKs that have been shown to be important targets in cancer^29^. For example, the RTK vascular endothelial growth factor receptor-2 (VEGFR-2) stands out as a key target in cancer treatment due to its important role in angiogenesis^30^. Based on the functional crosstalk of EGFR and VEGFR-2 through shared common downstream signalling pathways^31^, it is inferred that the simultaneous inhibition of both EGFR and VEGFR is an effective approach for overcoming the reported resistance in NSCLC^32^. In fact, it has been shown that VEGFR-2 inhibition enhances the cytotoxic effect of EGFR inhibitors, whereas, VEGFR-2 activation results in accelerated tumour growth independent of EGFR signalling, thereby facilitating the emergence of resistance to EGFR inhibitors^33^. Vandetanib **(**Caprelsa^®^) is an example of FDA approved quinazoline-based drug with dual EGFR and VEGFR-2 inhibitory activity^34^ (Figure 1).

In the current medical era, thiazole is identified as an important heterocyclic motif that emerged as a promising privileged scaffold in the anticancer drug discovery^35–37^, Several FDA approved anticancer drug incorporate the heterocycle thiazole, such as dabrafenib and dasatinib. Pyrazoline moiety, on the other hand, is a highly active heterocyclic nucleus possessing interesting biological activities^38^ like; anticancer^39–41^, anti-inflammatory^42^, antidepressant^43^, anticonvulsant^44^ and antimicrobial activities^45^.

**Figure 2.**
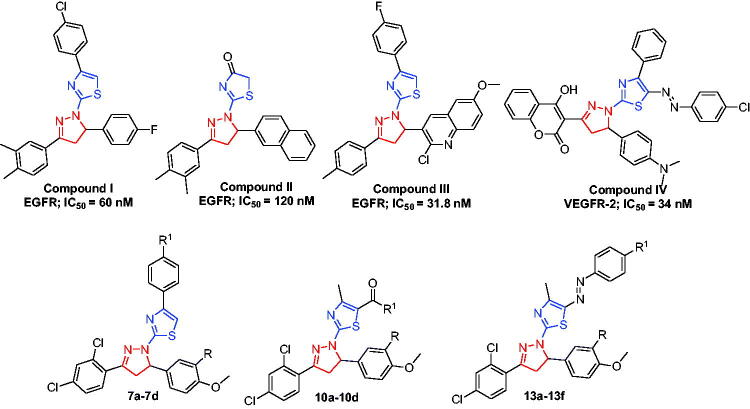
Structure of some reported thiazolyl-pyrazoline based EGFR and VEGFR-2 inhibitors (**I–IV**), and the target compounds in this study (**7a–7d**, **10a–10d** and **13a–13f**).

Recently, some research groups have devoted their efforts to hybridise both the thiazole and pyrazoline into a single frame work, producing novel series based on the thiazolyl-pyrazoline system which thereafter identified as a substantial scaffold in anticancer drug discovery^46^. Interestingly, there is a large number of reported compounds with potent cytotoxic effect based on thiazolyl-pyrazoline scaffold having EGFR (compounds **I**, **II** and **III**, Figure 2)^47–49^ and VEGFR-2 (compound **IV**, Figure 2)^50^ suppressing potentials. It is worth mentioning that none of these studies have developed dual or multiple kinase inhibitors, in addition they have not evaluated the biological activity against EGFR-mutated cancer cell lines.

Motivated by the above mentioned findings, here we sought to develop new different sets of novel thiazolyl-pyrazoline-based small molecules (**7a–7d**, **10a–10d** and 13**a–13f**) as potential dual EGFR/VEGFR-2 inhibitors with improved anticancer activities based on the molecular hybridisation between thiazole and pyrazoline motifs (Figure 2).

It is worth to mention that the reported pharmacophoric features required for EGFR inhibition include a central flat hetero-aromatic ring scaffold that should occupy the adenine binding pocket, a terminal hydrophobic head that occupies the hydrophobic sub-pocket, as well as a hydrophobic tail to occupy a second hydrophobic region^51^^,^^52^. Additionally, certain pharmacophoric features have been identified for the VEGFR-2 inhibitors including a flat hetero-aromatic moiety to be fitted in the ATP binding region, a terminal hydrophobic motif to achieve several hydrophobic interactions in the allosteric hydrophobic pocket, and the presence of H-bond acceptor (HBA) functionalities^53–55^. Interestingly, the target thiazolyl-pyrazoline-based small molecules (**7a–**7**d**, **10a–**10**d** and 13**a–**13**f**) achieved these features required for inhibition of both EGFR and VEGFR-2. Furthermore, the substitution pattern on the pendant phenyl moieties was selected so as to ensure different electronic and lipophilic environments which should manipulate the activity of the target thiazolyl-pyrazolines.

Once created, all derivatives were assessed for their potential inhibitory activity towards EGFR and VEGFR-2 kinases. Also, the inhibitory activities against a panel of 11 kinases (TEK, SYK, EPHB2, ABL1, LCK, CLK1, ROCK1, PKC, AKT1, CDK1, and CDK5) were further investigated to determine the selectivity profile. Then, all compounds were screened for their cytotoxic effect against nine cell lines derived from five tumour subpanels including leukaemia (K562, and KG-1a), breast (MCF-7, BT-549, and HCC70), lung (A549 and H441), colon (HCT116) and liver (HepG2) cancer cell lines. In addition, the anti-proliferative potential of the most potent EGFRWT inhibitors was analysed towards two EGFR mutated cancer cell lines; NCI-H1650 and NCI-H1975. Thereafter, the most efficient cells growth and kinase inhibitors **10b** and **10d** were selected to explore their possible cellular mechanism of action *via* cell cycle, apoptosis Annexin-V-FITC and migration assays in NSCLC A549 cells. Finally, *in silico* studies were carried out to explore the binding interactions of thiazolyl-pyrazolines **10b** and **10d** within the vicinity of ATP-binding sites on EGFR and VEGFR-2 kinase domains.

## 2. Results and discussion

### 2.1. Chemistry

**Scheme 1.**
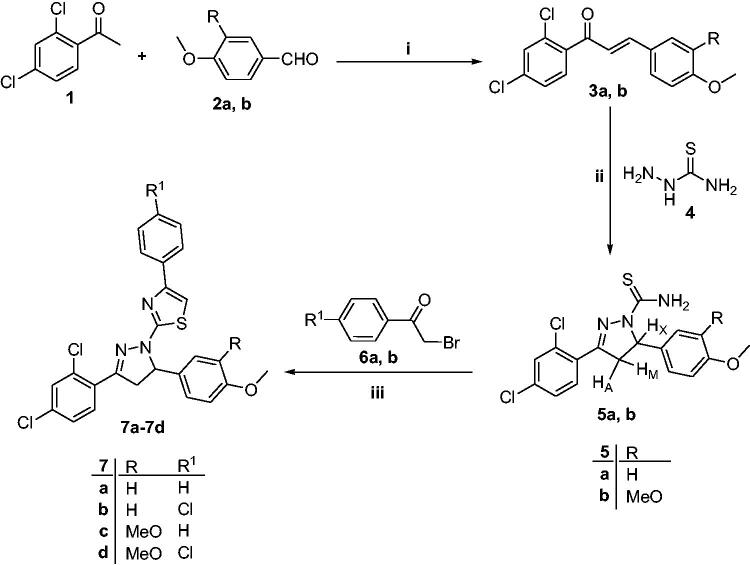
Synthetic pathway for preparation of intermediates **5a, b** and compounds **7a–7d**; reagents and reaction conditions: (i) 10% NaOH, EtOH, RT, 4 h; (ii) NaOH, EtOH, reflux, 6 h; (iii) EtOH, reflux, 4 h.

**Scheme 2.**
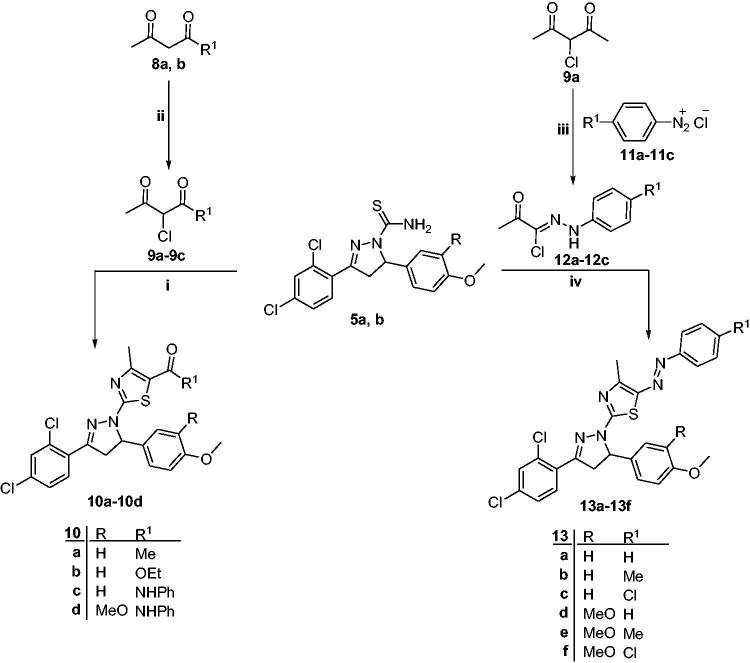
Synthetic pathway for preparation of compounds **10a–10d** and **13a–13f**; reagents and reaction conditions: (i) EtOH, reflux, 4 h; (ii) SO_2_Cl_2_, dry Et_2_O, 0–5 °C, 2 h; (iii) (a) NaNO_2_-HCl, 0–5 °C, 2 h; (b) NaOAc.3H_2_O, EtOH, 0–5 °C, 4 h; (iv) EtOH, reflux, 4 h.

The synthetic pathways adopted for preparation of the intermediates **5a**–**b** and the target thiazolyl-pyrazoline derivatives (**7a–**7**d**, **10a–**10**d** and **13a–**13**f**) were depicted in Schemes 1 and 2. In Scheme 1, chalcones **3a, b** were prepared *via* reaction of 2,4-dichloroacetophenone **1** with the appropriate aromatic aldehydes **2a, b**, adopting the base catalysed Claisen–Schmidt condensation reaction as reported^56^ (Scheme 1). As active intermediates, chalcones **3a, b** were used to prepare pyrazolines **5a, b** by the reaction with thiosemicarbazide **4** in the presence of sodium hydroxide as a catalyst (Scheme 1). Pyrazoline formation occurs *via* two steps; the first one is the condensation reaction between the chalcones **3a, b** and thiosemicarbazide **4**, whereas, the second step is the intramolecular cyclisation through *Michael* addition of the NH group to the double bond^57^ (Scheme 1). Structures of compounds **5a, b** were confirmed by microanalyses and spectral data. IR spectra of **5a, b** showed NH_2_ bands at 3418, 3444 and 3247, 3322 cm^−1^, respectively, in addition to two bands at 1236, 1254 and 828, 833 cm^−1^ referring to C=S. ^1^H NMR spectra of **5a** displayed three doublets of doublets for H_A_, H_M_ and H_X_ of the pyrazoline ring at *δ* 3.18, 4.03 and 5.87 ppm, respectively. This AMX pattern confirmed the formation of the pyrazoline ring and the presence of two diastereotopic protons at C-4 (H_A_ and H_M_) and one single proton at C-5 (H_X_)^58^. Furthermore, ^1^H NMR spectra of **5a, b** appeared two D_2_O exchangeable singlet signals of NH_2_ around *δ* 7.75, 7.76 and 8.13 ppm, respectively. In addition, ^13^C NMR spectra of **5a, b** revealed two signals for C-4 and C-5 carbons of pyrazoline ring at *δ* 45.7 and 63.0, 63.3 ppm, respectively.

Furthermore, reaction of carbothioamide derivatives **5a, b** with 1-aryl-2-bromoethanones **6a, b** in refluxing ethanol yielded the corresponding 4-(aryl)-2-(3-(2,4-dichlorophenyl)-5-(4-aryl)-4,5-dihydro-1*H*-pyrazol-1-yl)thiazoles **7a–7d**, respectively, *via* Hantzsch thiazole synthesis^54^^,^^55^ (Scheme 1). Initially, nucleophilic substitution of Br in phenacyl bromide by the S-atom of thioamide generates the isothiourea, which subsequently undergoes cyclocondensation and water elimination to furnish the thiazole ring^57^ (Scheme 1). The latter synthesised compounds **7a–7d** were confirmed based on their elemental analysis and spectral data. IR spectra showed the disappearance of the characteristic band of NH_2_ in the region of 3418, 3444 and 3247, 3322 cm^−1^. Their ^1^H NMR spectra showed an increase in the aromatic integration due to thiazole proton and protons of the extra phenyl ring at *δ* 6.91–7.82 ppm. ^13^C NMR spectra of **7b, c** showed peak around *δ* 105 ppm corresponding to C-5 of thiazole ring along with an increase in signals assigned for aromatic carbons at *δ* 111.6–164.8 ppm.

Similarly, thiazole derivatives **10a–10d** were obtained from the reaction of the carbothioamides **5a, b** with the appropriate *α*-chloro-1,3-dicarbonyl compounds **9a–9c** in absolute ethanol through the Hantzsch thiazole synthesis^49^^,^^55^ (Scheme 2). Compounds **10a–10d** were confirmed based on their elemental analysis and spectral data.

IR spectra of **10a–10d** showed a band at 1637–1669 cm^−1^ due to C=O group, while the spectra of **10c, d** revealed a band at 3257 and 3262 cm^−1^, respectively corresponding to NH group. ^1^H NMR spectra of **10a–10d** showed a signal at *δ* 2.36–2.54 ppm attributed to the protons of the methyl group at the 4-position of the thiazole ring. Moreover, ^1^H NMR spectrum of **10a** showed a singlet signal at *δ* 2.40 ppm attributed to CH_3_ protons of the acetyl group. Furthermore, ^1^H NMR spectrum of **10b** showed two signals at *δ* 1.35 and 4.29 ppm, respectively attributed to the CH_3_ and CH_2_ protons of the ethyl group. Additionally, ^1^H NMR spectra of **10c**, **d** displayed a D_2_O exchangeable singlet signal of NH proton at *δ* 9.72, 8.14 ppm, respectively, in addition to an increase in the aromatic integration at *δ* 6.78–7.81 ppm assignable to the extra phenyl ring. ^13^C NMR of compound **10a** showed signals assigned to CH_3_ carbon of the thiazole ring, CH_3_ and C=O carbons of the acetyl group at *δ* 19.1, 30.1 and 189.7 ppm, respectively. Additionally, ^13^C NMR spectrum of **10b** displayed signals attributed to ethyl carbons at *δ* 14.7 and 60.7 ppm along with signal due to ester carbonyl carbon at *δ* 165.3 ppm. Furthermore, ^13^C NMR spectrum of **10d** revealed signals at *δ* 18.2 and 163.8 ppm due to CH_3_ carbon of the thiazole ring and C=O, respectively.

Finally, 2-(3-(2,4-dichlorophenyl)-5-(aryl)-4,5-dihydro-1*H*-pyrazol-1-yl)-4-mthyl-5-(aryldiazenyl)thiazole derivatives **13a–13f** were prepared by heating the carbothioamide derivatives **5a, b** with 2-oxo-*N*-arylpropanehydrazonyl chlorides **12a–12c** in absolute ethanol^56^^,^^57^ (Scheme 2). 2-Oxo-*N*-arylpropanehydrazonyl chlorides **12a–12c** were prepared through a coupling reaction between *α*-chloroacetylacetone and aryl diazonium salts *via* Japp–Klingeman rearrangement^59^.

IR spectra of compounds **13a–13f** showed a band at 1582–1589 cm^−1^ referring to (N = N) in addition to the absence of the band corresponding to NH_2_ group at 3418, 3444 and 3247, 3322 cm^−1^. ^1^H NMR spectra of **13b**, **e**, **f** revealed singlet signals at *δ* 2.53–2.61 ppm equivalent to CH_3_ protons of the thiazole ring in addition to an increase in the integration of the aromatic protons at *δ* 6.78–7.90 ppm. Also, ^1^H NMR spectra of **13b**, **e** showed additional singlet signals at *δ* 2.42 and 2.41 ppm attributed to the three protons of 4-CH_3_, respectively. ^13^C NMR of **13a–13e** showed a signal for CH_3_ carbon of the thiazole ring at *δ* 16.1–16.6 ppm along with an increase in the number of signals of aromatic carbons at *δ* 109.4–165.3 ppm. Also, ^13^C NMR of **13b, e** revealed the presence of a signal at *δ* 21.4 ppm indicating an additional CH_3_ carbon at the phenyldiazenyl ring.

### 2.2. Biological evaluation

#### 2.2.1. EGFR and VEGFR-2 kinase inhibitory activities of thiazolyl-pyrazoline derivatives

In the current study, the potential inhibitory activity of target thiazolyl-pyrazoline derivatives (**7a–7d**, **10a–10d** and **13a–13f**) towards EGFR and VEGFR-2 were examined, with the aim of exploring the plausible mechanism. The IC_50_ values of the tested compounds were evaluated compared to reference EGFR inhibitor (gefitinib) and VEGFR-2 inhibitor (vandetanib), Table 1.

**Table 1.** EGFR and VEGFR-2 kinases inhibitory activity of target thiazolyl-pyrazoline derivatives. 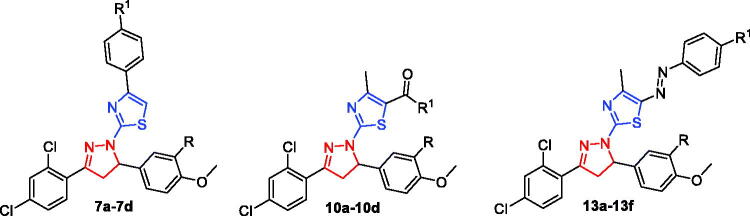

Compound	R	R_1_	IC_50_ (nM) ± SEM	
			EGFR	VEGFR-2
**7a**	H	H	145.1 ± 2.0	124.9 ± 1.4
**7b**	H	Cl	114.2 ± 0.4	195.2 ± 4.6
**7c**	OCH_3_	H	511.2 ± 3.2	915.2 ± 4.3
**7d**	OCH_3_	Cl	132.9 ± 1.2	185.5 ± 2.3
**10a**	H	CH_3_	274.0 ± 2.3	450.3 ± 8.3
**10b**	H	OCH_2_CH_3_	40.7 ± 1.0	78.4 ± 1.5
**10c**	H	NHC_6_H_5_	130.7 ± 1.1	102.2 ± 4.3
**10d**	OCH_3_	NHC_6_H_5_	32.5 ± 2.2	43.0 ± 2.4
**13a**	H	H	973.0 ± 2.0	1151.2 ± 4.3
**13b**	H	CH_3_	179.4 ± 0.0	214.3 ± 4.0
**13c**	H	Cl	134.5 ± 1.5	173.3 ± 2.6
**13d**	OCH_3_	H	93.6 ± 3.0	335.8 ± 6.3
**13e**	OCH_3_	CH_3_	67.7 ± 2.0	216.4 ± 4.3
**13f**	OCH_3_	Cl	107.13 ± 1.2	113.3 ± 1.8
**Gefitinib**	**–**	**–**	55.74 ± 1.0	45.3 ± 1.2
**Vandetanib**	**–**	**–**	43.14 ± 1.3	29.1 ± 1.0

Examination of the obtained results (Table 1) hinted out that the target thiazolyl-pyrazoline derivatives (**7a–7d**, **10a–10d** and **13a–13f**) have better kinase inhibitory activity towards EGFR (IC_50_ range: 32.5–973.0 nM) than VEGFR-2 (IC_50_ range: 43.0–1151.2 nM), except for compounds **7a** and **10c**. As shown in Table 1, compounds **10b** and **10d** displayed the highest inhibitory activity against EGFR (IC_50_ = 40.7 and 32.5 nM, respectively) as well as VEGFR-2 (IC_50_ = 78.4 and 43.0 nM, respectively), due to the ability of the carbonyl group in the ester functionality to form hydrogen bonds with the key amino acids in both EGFR and VEGFR-2 active sites. Moreover, compounds **7a**, **7b**, **7d**, **10a**, **10c** and **13b–13d** exhibited moderate inhibitory activity against EGFR (IC_50_: 67.7–274.0 nM) and VEGFR-2 (IC_50_: 102.2–450.3 nM). On the other hand, compounds **7c** and **13a** displayed the lowest activity against EGFR (IC_50_ = 511.2 and 973.0 nM, respectively) and VEGFR-2 (IC_50_ = 915.2 and 1151.2 nM, respectively).

The following structure–activity relationships (SARs) can be concluded from the kinases inhibition data displayed in Table 1. Regarding the EGFR inhibitory activity of the first series **7a–**7**d**, it was found that substitution of the pendant phenyl ring at C-4 of the thiazole moiety (compounds **7b** and **7d**; IC_50_ = 114.2 ± 0.4 and 132.9 ± 1.2 nM, respectively) elicited an enhancement of effectiveness towards EGFR in comparison to the unsubstituted analogues (compounds **7a** and **7c**; IC_50_ = 145.1 ± 2.0 and 511.2 ± 3.2 nM, respectively). In contrast, the 3,4-dimethoxy substitution for the pyrazoline C-5 phenyl ring (compounds **7c** and **7d**; IC_50_ = 511.2 ± 3.2 and 132.9 ± 1.2 nM, respectively) resulted in a worsening of inhibitory activity against EGFR in comparison to the 4-methoxy monosubstituted counterparts (compounds **7a** and **7b**; IC_50_ = 145.1 ± 2.0 and 114.2 ± 0.4 nM, respectively).

Moreover, the obtained results for the second series **10a–***10***d** towards EGFR revealed that the incorporation of the ester functionality at C-5 of the thiazole moiety (compound **10b**; IC_50_ = 40.7 ± 1.0 nM) was more beneficial for the activity more than the acetyl (compound **10a**; IC_50_ = 274.0 ± 2.3 nM) and amide (compound **10c**; IC_50_ = 130.7 ± 1.1 nM) functionalities. In addition, the 3,4-dimethoxy substitution for the pyrazoline C-5 phenyl ring in the amide-bearing **10d** led to the best EGFR inhibition in this study; IC_50_ = 32.5 ± 2.2 nM. In a similar fashion, incorporation of a 3,4-dimethoxyphenyl moiety within the third series **13a–***13***f** (compounds **13d–***13***f**; IC_50_ = 93.6 ± 3.0, 67.7 ± 2.0 and 107.13 ± 1.2 nM, respectively) elicited an enhancement of EGFR inhibitory activity in comparison to the monosubstituted analogues (compounds **13a–***13***c**; IC_50_ = 973.0 ± 2.0, 179.4 ± 0.0 and 134.5 ± 1.5 nM, respectively).

On the other hand, the SAR outcomes for VEGFR-2 inhibition pointed out that utilisation of the ester group at C-5 of the thiazole moiety within the second series **10a–10d** (compound **10 b**; IC_50_ = 78.4 ± 1.5 nM) led to an enhanced activity more than the acetyl (compound **10a**; IC_50_ = 450.3 ± 8.3 nM) and amide (compound **10c**; IC_50_ = 102.2 ± 4.3 nM) groups. Similar to the SAR for EGFR inhibition, grafting a second methoxy group for the amide-bearing counterpart **10c** (IC_50_ = 102.2 ± 4.3 nM) produced compound **10d** that identified as the most potent VEGFR-2 inhibitor in this work (IC_50_ = 43.0 ± 2.4 nM). Further analysis of the VEGFR-2 inhibition data for the third series **13**, highlighted that both the *para*-substitution of the appended phenyl ring at C-5 of the thiazole moiety, and the 3,4-dimethoxy substitution for the pyrazoline C-5 phenyl ring resulted in an improvement of the VEGFR-2 inhibitory activity.

#### 2.2.2. Western blotting of EGFR and VEGFR-2 in A549 cells

**Figure 3.**
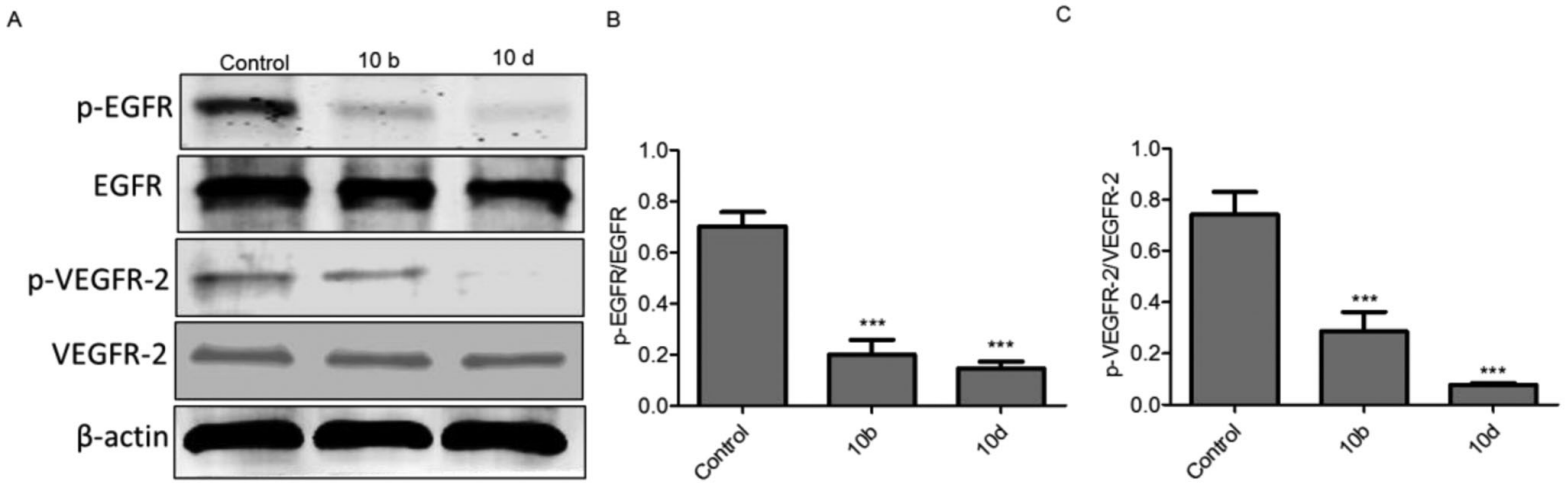
Thiazolyl-pyrazoline derivatives downregulated p-EGFR and p-VEGFR-2 protein levels (A) Western blotting analysis of the expression of p-EGFR, EGFR, p-VEGFR-2 and VEGFR-2 in A549 cells treated with **10b** or **10d** and untreated cells used as a negative control. β-actin served as a loading control. (B) The relative protein expression level of p-EGFR normalised with total EGFR was quantified by the Image J software (C) The relative protein expression level of p-VEGFR-2 normalised with total VEGFR-2. The values are expressed as the mean ± SEM based on three different experiments. ***p < 0.001 indicate a significant difference compared with control.

To confirm the ability of thiazolyl-pyrazolines derivatives to dual inhibit the activity of EGFR and VEGFR-2 kinases, we examined the effect of the most potent compounds **10b** and **10d** on the expression levels of p-EGFR, EGFR, p-VEGFR-2 and VEGFR-2 proteins in A549 cells using Western blot analysis. As shown in Figure 3, the protein expression levels of p-EGFR and p-VEGFR-2 were significantly downregulated by compounds **10b** and **10d**.

#### 2.2.3. Kinase selectivity profiling

As compounds **10b** and **10d** showed high potency against EGFR and VEGFR-2 kinases (Table 1), we further investigated their inhibitory activities against 11 kinases (TEK, SYK, EPHB2, ABL1, LCK, CLK1, ROCK1, PKC, AKT1, CDK1, and CDK5) to determine their selectivity profile (Table 2).

**Table 2.** Kinase selectivity profile of **10b** and **10d.**

Kinase	IC_50_ (nM)	
	10b	10d
TEK	>2000	>2000
SYK	1120 ± 22	870 ± 31
EPHB2	>2000	>2000
ABL1	>2000	>2000
LCK	>2000	>2000
CLK1	1069 ± 54	798 ± 19
ROCK1	>2000	>2000
PKC	>2000	>2000
AKT1	816 ± 38	702 ± 24
CDK1	310 ± 12	245 ± 7
CDK5	1136 ± 20	993 ± 15

Interestingly, compounds **10b** and **10d** displayed moderate inhibition against CDK1 (IC_50_ = 310 and 245 nM, respectively) and low inhibition against SYK, CLK1, AKT1 and CDK5 (IC_50_ range: 702–1136 nM). Meanwhile, **10b** and **10d** did not exhibit measurable inhibition (with IC_50_ > 2000 nM) against TEK, EPHB2, ABL1, LCK, ROCK1 and PKC kinases, demonstrating significant selectivity of **10b** and **10d** towards EGFR and VEGFR-2 kinases.

#### 2.2.4. Anti-proliferative activity of thiazolyl-pyrazoline derivatives

To evaluate the anti-proliferative activity of the synthesised thiazolyl-pyrazoline derivatives (**7a–7d**, **10a–10d**, and **13a–13f**), a preliminary *in vitro* one dose (10 µM) anticancer assay was performed. Nine cell lines derived from five tumour subpanels were examined; leukaemia (K562 and KG-1a), breast (MCF-7, BT-549 and HCC70), lung (A549 and H441), colon (HCT116) and liver (HepG2) cancer cell lines. The compounds were added at a single concentration (10 µM) and the cultures were incubated for 24 h, then the per cent growth inhibition (%GI) was calculated.

As shown in Tables 3 and 4, compounds **10b** and **10d** revealed promising cell growth inhibition activity against almost all the screened cell lines (%GI ranging from 31.0% to 95.3%), especially against the non-small cell lung cancer A549 and H441 cell lines (%GI ranging from 79.1% to 95.3%). Other compounds showed moderate to weak anti-proliferative effect against the tested cell lines; however, the non-small lung cancer cells (A549 and H441) were more sensitive to the target thiazolyl-pyrazoline derivatives.

**Table 3.** % Growth inhibition ± SEM of thiazolyl-pyrazoline derivatives (**7a–7d**, **10a–10d** and **13a–13f**) towards leukaemia (K562 and KG-1a) and breast cancer (MCF-7, BT-549 and HCC70) cell lines.

Compound	K562	KG-1a	MCF-7	BT-549	HCC70
**7a**	14.2 ± 2.1	18.4 ± 1.3	0.07 ± 0.0	26.4 ± 3.6	27.3 ± 1.0
**7b**	21.5 ± 3.0	21.7 ± 4.6	25.9 ± 1.2	5.2 ± 1.0	19.6 ± 0.3
**7c**	5.2 ± 0.5	4.3 ± 0.7	9.2 ± 0.9	3.1 ± 0.6	7.3 ± 1.2
**7d**	22.0 ± 1.6	25.1 ± 2.0	2.2 ± 0.6	18.0 ± 2.7	17.3 ± 0.6
**10a**	12.2 ± 1.9	22.2 ± 1.6	13.6 ± 2.9	9.4 ± 1.0	24.3 ± 2.5
**10b**	**54.2 **±** **3.8	**31.0 **±** **2.5	**48.5 **±** **2.3	**47.2 **±** **4.9	**41.5 **±** **2.1
**10c**	11.2 ± 1.0	9.1 ± 1.0	2.5 ± 0.8	22.3 ± 0.8	18.3 ± 1.6
**10d**	**60.2 **±** **2.3	**57.2 **±** **3.4	**60.9 **±** **4.3	**40.1 **±** **2.0	**54.1 **±** **2.8
**13a**	2.2 ± 0.2	4.1 ± 0.9	17.8 ± 1.0	10.3 ± 1.1	15.3 ± 1.4
**13b**	11.0 ± 0.6	7.1 ± 0.2	3.4 ± 0.5	5.1 ± 0.3	8.3 ± 0.9
**13c**	5.2 ± 0.9	12.4 ± 1.6	10.4 ± 2.9	8.7 ± 1.9	1.1 ± 0.1
**13d**	18.2 ± 1.8	24.1 ± 2.7	11.9 ± 1.8	14.2 ± 2.2	22.3 ± 1.3
**13e**	24.0 ± 2.0	15.2 ± 1.8	1.8 ± 0.6	12.5 ± 1.5	16.1 ± 0.9
**13f**	31.5 ± 1.0	20.0 ± 2.8	0.8 ± 0.2	24.6 ± 1.3	6.3 ± 1.0

**Table 4.** % Growth inhibition ± SEM of thiazolyl-pyrazoline derivatives (**7a–7d**, **10a–10d** and **13a–13f**) towards lung (A549 and H441), colon (HCT116) and liver (HepG2) cancer cell lines.

Compound	A549	H441	HepG2	HCT116
**7a**	34.9 ± 4.3	36.1 ± 2.1	0.5 ± 0.0	0.5 ± 0.1
**7b**	45.2 ± 2.9	35.2 ± 1.2	34.4 ± 1.4	18.8 ± 1.9
**7c**	8.9 ± 1.1	16.2 ± 0.3	17.1 ± 2.7	4.9 ± 0.8
**7d**	31.2 ± 1.4	34.8 ± 2.4	5.1 ± 1.0	0.7 ± 0.0
**10a**	32.3 ± 2.2	6.3 ± 0.7	28.0 ± 4.3	19.0 ± 2.6
**10b**	**88.3 **±** **4.9	**79.1 **±** **2.6	**50.6 **±** **2.2	**37.7 **±** **3.9
**10c**	28.9 ± 2.0	8.7 ± 0.1	11.9 ± 1.1	7.9 ± 1.1
**10d**	**95.3 **±** **5.3	**89.2 **±** **3.9	**65.5 **±** **3.9	**56.5 **±** **2.0
**13a**	11.0 ± 0.8	22.2 ± 0.9	15.8 ± 2.2	19.5 ± 1.4
**13b**	16.2 ± 1.3	23.3 ± 2.4	0.08 ± 0.0	0.8 ± 0.2
**13c**	14.8 ± 1.0	19.9 ± 1.7	14.2 ± 1.0	8.7 ± 0.6
**13d**	12.6 ± 2.7	38.9 ± 2.4	18.6 ± 2.5	10.1 ± 3.5
**13e**	30.3 ± 2.4	46.9 ± 3.9	0.3 ± 0.0	9.9 ± 0.8
**13f**	47.5 ± 3.1	17.3 ± 0.6	0.9 ± 0.3	0.2 ± 0.0

Overexpression of EGFR and VEGFR-2 is well-reported in the literature to be significantly correlated with the induction of the NSCLC cells proliferation^31^. Thereafter, the quantitative IC_50_ values have been determined for thiazolyl-pyrazoline derivatives (**7a–7d**, **10a–10d** and **13a–13f**) towards the most susceptible cell lines at the preliminary assay (non-small lung cancer A549 and H441 cell lines), at testing concentrations of 1, 5, 10, 25, 50 or 100 µM for 24 h. The antitumor drugs gefitinib and vandetanib were used as reference drugs in this MTT cytotoxicity assay (Table 5).

**Table 5.** Inhibitory activities of thiazolyl-pyrazoline derivatives (**7a–7d**, **10a–10d** and **13a**–**13f**) against cancerous and normal lung cells. Values represent the mean IC_50_ values (µM) ± SEM (*n* = 3) for each drug. Compared IC_50_s; a compounds versus gefitinib and b compounds versus Vandetanib.

Compound	IC_50_ (µM) ± SEM		
	A549	H441	WI-38
**7a**	22.5 ± 1.2^a^ ns, ^b^***	38.4 ± 2.3^a^***, ^b^***	88.2 ± 4.2 ^a^***, ^b^***
**7b**	12.0 ± 0.4^a^***, ^b^***	26.5 ± 1.1^a^***, ^b^***	60.6 ± 3.1^a^***, ^b^***
**7c**	>100	>100	>100
**7d**	20.2 ± 2.0^a^ ns, ^b^***	29.1 ± 3.5^a^***, ^b^***	40.1 ± 2.7^a^*, ^b^***
**10a**	49.1 ± 3.2^a^***, ^b^***	>100	>100
**10b**	4.2 ± 0.8^a^***, ^b^ns	4.8 ± 0.1^a^*, ^b^ns	72.4 ± 7.2^a^***, ^b^***
**10c**	23.5 ± 1.3^a^ ns, ^b^***	45.2 ± 2.3^a^***, ^b^***	>100
**10d**	2.9 ± 0.2^a^***, ^b^ns	3.8 ± 0.5^a^*, ^b^ns	51.4 ± 3.5^a^***, ^b^***
**13a**	>100	94.4 ± 5.5^a^***, ^b^***	>100
**13b**	59.1 ± 3.2^a^***, ^b^***	24.2 ± 1.8^a^***, ^b^***	>100
**13c**	46.3 ± 2.5^a^***, ^b^***	33.5 ± 3.9^a^***, ^b^***	91.4 ± 8.9^a^***, ^b^***
**13d**	57.1 ± 4.1^a^***, ^b^***	21.7 ± 1.3^a^***, ^b^***	64.2 ± 2.8^a^***, ^b^***
**13e**	19.1 ± 1.3^a^*, ^b^***	15.2 ± 3.6^a^***, ^b^***	87.0 ± 4.2^a^***, ^b^***
**13f**	13.7 ± 2.2^a^**, ^b^***	24.8 ± 1.4^a^***, ^b^***	>100
Gefitinib	22.3 ± 1.4	7.1 ± 1.3	35.1 ± 2.0
Vandetanib	3.4 ± 1.0	3.9 ± 0.8	8.6 ± 0.4

*p < 0.05, **p < 0.01, ***p < 0.001 and ns = non-significant.

The results illustrated in Table 5 showed that compounds **10b** and **10d** have the best anti-proliferative activity, herein reported, against A549 (IC_50_ = 4.2 and 2.9 µM, respectively) and H441 (IC_50_ = 4.8 and 3.8 µM, respectively) cell lines, whereas compounds **7c** and **13a** have non-significant anti-proliferative activity (IC_50_ more than 100 μM). Other compounds showed moderate to low anti-proliferative effect against A549 (IC_50_ range: 12.0–59.1 µM) and H441 (IC_50_ range: 15.2–94.4 µM). Interestingly, the anti-proliferative effect of compounds **10b** and **10d** was higher than the reference drug gefitinib and was comparable to vandetanib. In addition, compounds **7a**, **7b**, **7d**, **10c**, **13e** and **13f** exerted moderate anticancer activity against A549 cell lines with IC_50_s values spanning between 12.0 and 23.5 µM, whereas compounds **7b**, **7d**, **13b** and **13d–13f** showed moderate growth inhibitory activity towards H441 cells with IC_50_s spanning in the range 15.2–29.1 µM, Table 5.

It is noteworthy that the synthesised thiazolyl-pyrazoline derivatives (**7a–7d**, **10a–10d**, and **13a–13f**) showed weak or non-significant cytotoxicity to human normal WI-38 lung fibroblast cells (IC_50_ range: 40.1 ± 2.7 – >100 µM) (Table 5). The mean tumour selectivity value calculated for compounds **10b** and **10d** was found to be 16.1 and 15.3, respectively, which reveals good selectivity towards tumour cells with efficient safety index (Table 6).

**Table 6.** Cytotoxic effect of **10b** and **10d** towards human non-tumorigenic lung WI-38 cells and mean tumour selectivity index (S. I.) (WI-38/A549 and H441).

Compound	IC_50_ (µM) ± SEM			Mean tumour selectivity
	WI-38	A549	H441	
**10b**	72.4 ± 7.2	4.2 ± 0.8	4.7 ± 0.1	16.1
**10d**	51.4 ± 3.5	2.9 ± 0.2	3.8 ± 0.5	15.3

#### 2.2.5. Effect of thiazolyl-pyrazoline derivatives on EGFR-mutated NSCLC cell lines

**Figure 4.**
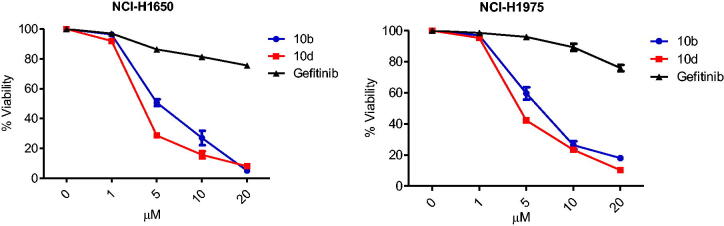
Effect of thiazolyl-pyrazoline derivatives on EGFR mutated NSCLC cell lines; dose–response curves for **10b** and **10d** in comparison with gefitinib control. The percentage of viable cells is shown relative to that of untreated control cells. Data were presented as means ± standard error mean (SEM) of three independent experiments.

The prolonged use of the TKIs targeting EGFR or VEGFR-2 kinases led to drug resistance due to mutations development and toxicity. In this study, we analysed the anti-proliferative potential of the most potent EGFRWT inhibitors **10b** and **10d** towards two EGFR mutated cancer cell lines; NCI-H1650 possesses exon19 deletion (delE746-A750) mutation of EGFR and NCI-H1975 carries L858R/T790M double mutation of EGFR. Interestingly, compounds **10b** and **10d** exhibited significant growth inhibitory activity against NCI-H1650 (IC_50_ = 5.7 and 3.5 µM, respectively) and NCI-H1975 (IC_50_ = 6.2 and 4.4 µM, respectively) Table 7 and Figure 4.

**Table 7.** Inhibitory activity of thiazolyl-pyrazoline derivatives **10 b** and **10d** against EGFR-mutated NSCLC cell lines.

Compound	IC_50_ (µM)	
	NCI-H1650	NCI-H1975
**10b**	5.7 ± 0.5	6.2 ± 0.9
**10d**	3.5 ± 0.2	4.4 ± 0.6
**Gefitinib**	>20	>20

Moreover, the obtained results indicated that both **10b** and **10d** were much more sensitive towards NCI-H1650 and NCI-H1975 cells than gefitinib (IC_50_ > 20 µM towards the two examined cell lines). Accordingly, **10b** and **10d** may serve as a lead compounds for the treatment of gefitinib-resistant EGFR mutant NSCLC.

#### 2.2.6. Thiazolyl-pyrazoline derivatives inhibited cancer cell migration

**Figure 5.**
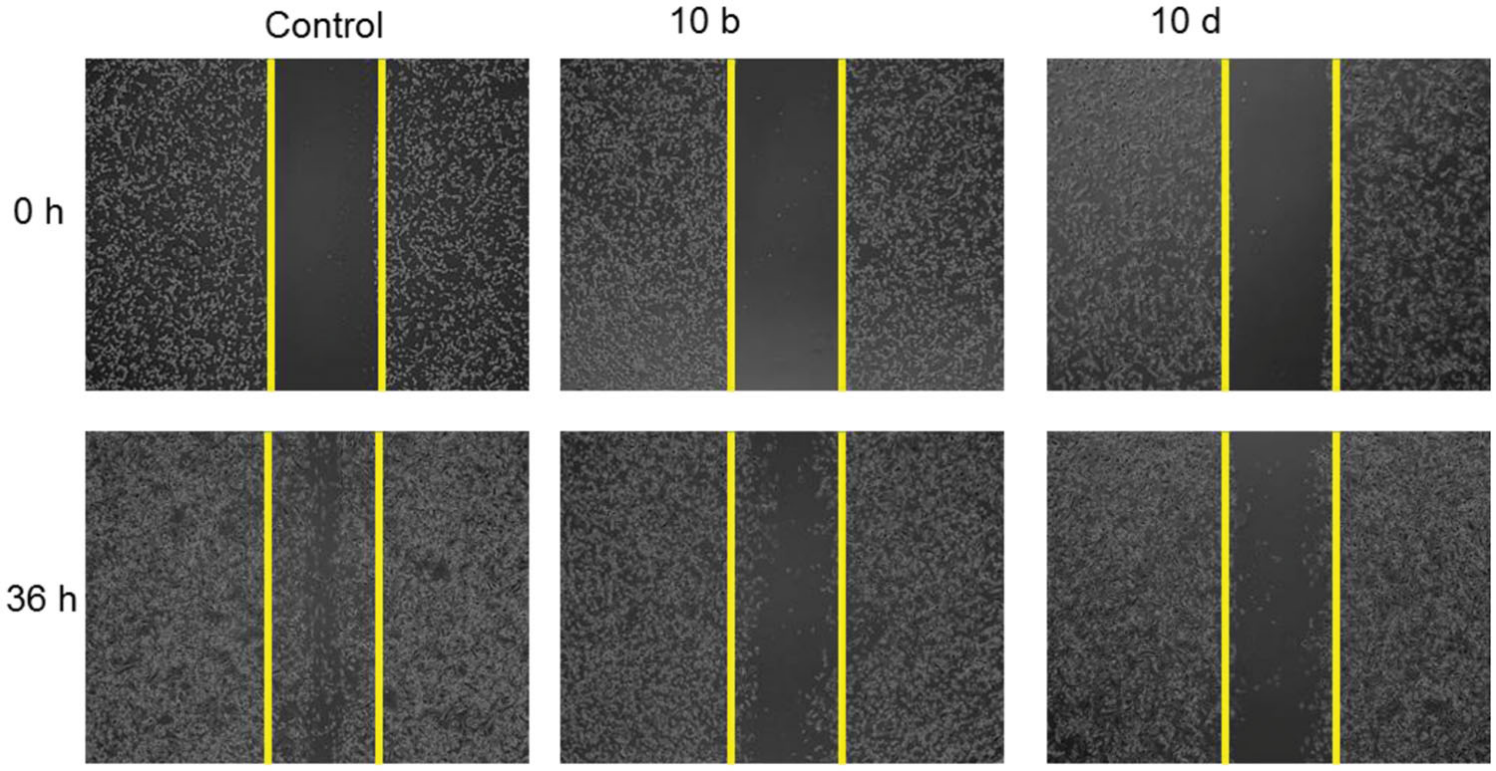
Effect of compounds **10b** and **10d** on A549 cells migration. Scratch wound healing assay was used to analyse the inhibition of cell migration in A549 cells treated for 36 h with either **10b** or **10d** and the untreated cells served as a negative control. Representative images of scratched areas in confluent A549 cell layers.

As the thiazolyl-pyrazoline derivatives **10b** and **10d** showed potent inhibitory effect against VEGFR-2 Kinase that plays an important role in cancer cell migration, we investigated the effect of **10b** and **10d** on the A549 cancer cell migration using wound healing assay. As shown in Figure 5, compounds **10b** and **10d** were effective in reducing the cancer cell migration compared to the control, highlighting that the VEGFR-2 kinase inhibitory effect of **10b** and **10d** is the engine for the inhibition of the cancer cell migration.

#### 2.2.7. Thiazolyl-pyrazoline derivatives induced G_2_/M cell cycle arrest

**Figure 6.**
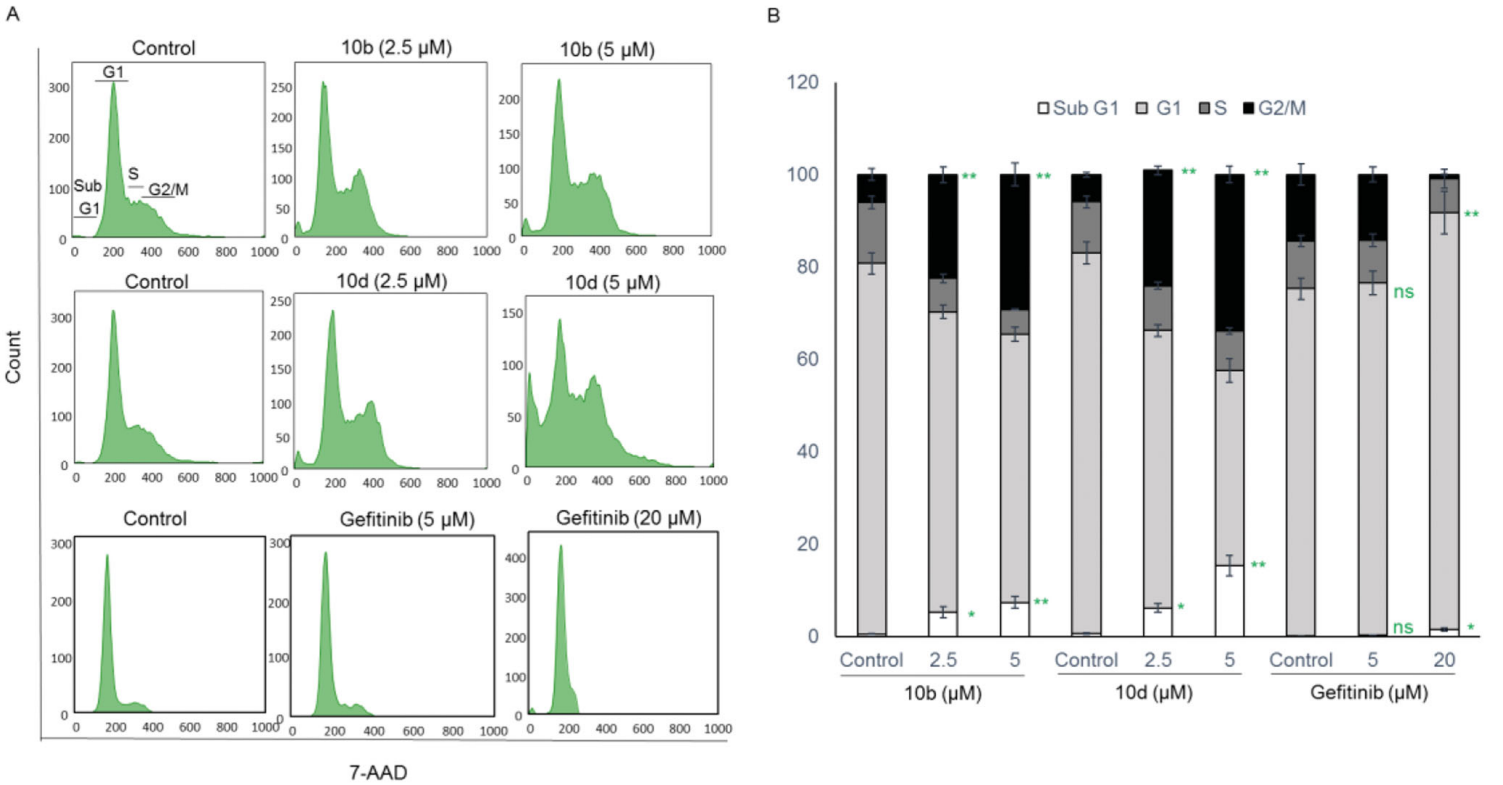
Compounds **10b** and **10d** arrested the cell cycle at a G2/M phase while gefitinib arrested the cell cycle at a G1 phase in A549 cells. (A) Representative cell cycle distribution data are shown by flow cytometric analysis upon incubation with 0 (control), 2.5 or 5 μM of **10b** or **10d** or with 0 (control), 5 or 20 μM of gefitinib for 24 h. (B) Quantification of cell cycle distribution upon 24 h incubation with **10b** or **10d**. The values are means ± SEM of three different experiments. *p < 0.05 and **p < 0.01 indicate significant differences compared with control. ns = non-significant.

In general, the anticancer agents inhibit the proliferation of cancerous cells by arresting cell division at various checkpoints. These checkpoints present at G_1_/S phase, S-phase and G_2_/M phases. Therefore, we tested the effect of the most potent compounds (**10b** and **10d**) on cell cycle progression in A549 cell line. Results in Figure 6 obviously indicate that compounds **10b** and **10d** arrested the cell cycle at a G_2_/M phase (29.2% and 33.92%, respectively) when compared to the untreated controls (6.06% and 5.93%, respectively). Parallel to these findings, the cell population in G_1_ and S phases decreased after treatment with **10b** and **10d** (Figure 6(A,B)).

On the other hand, gefitinib (20 µM) significantly arrested the cell cycle at G1 phase. This difference between the synthesised compounds and gefitinib in the arrested cell cycle phase could be attributed to the moderate inhibitory effect of **10b** and **10d** on the CDK1 kinase (IC_50_ = 310 and 245 nM, respectively) that regulates the G2/M phase transition. This result strongly suggests that the synthesised thiazolyl-pyrazoline derivatives inhibit the proliferation of A549 cells by arresting cell cycle at G_2_/M phase.

#### 2.2.8. Thiazolyl-pyrazoline derivatives induced apoptosis

**Figure 7.**
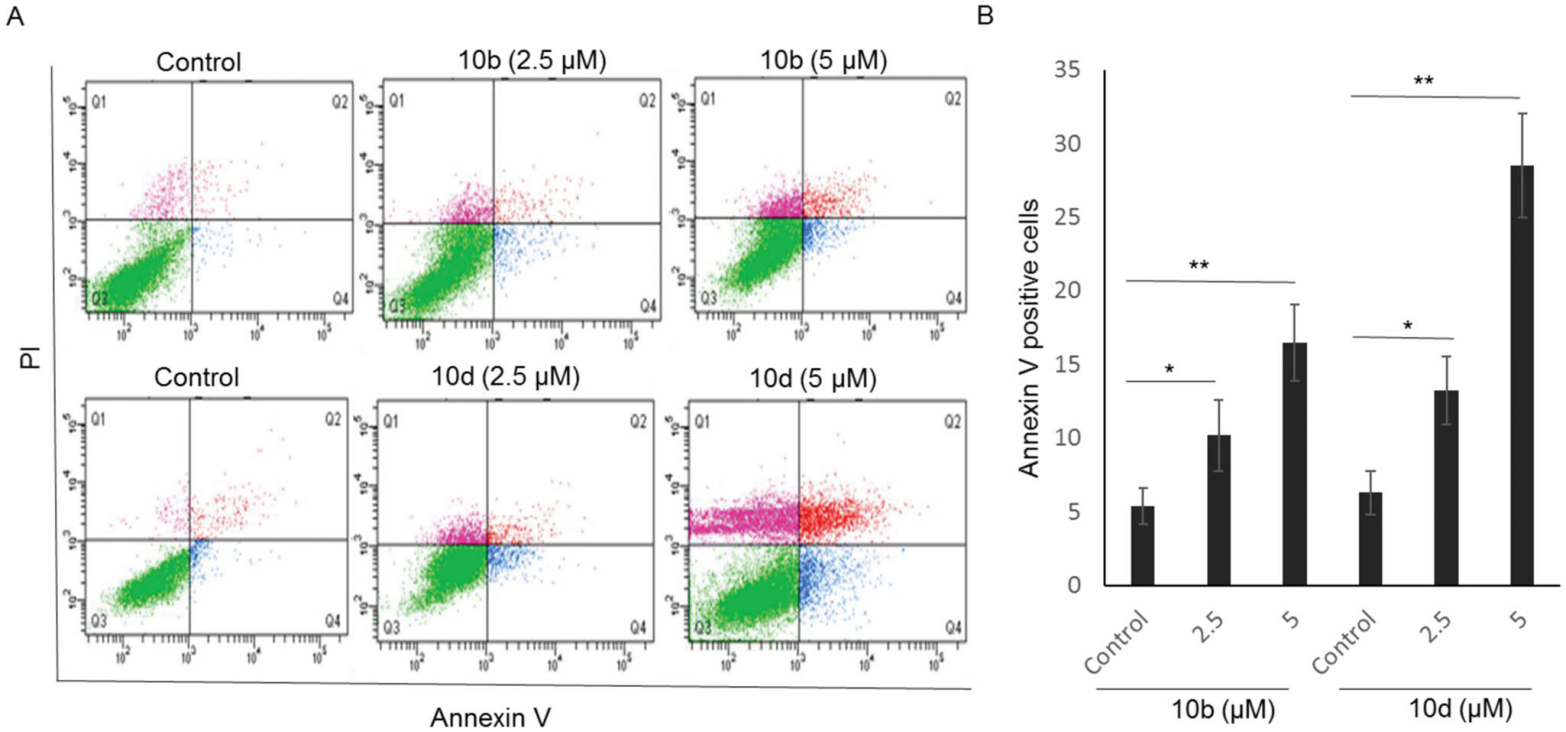
Apoptotic effect of compounds **10b** and **10d** on A549 cells. (A) Representative cytograms of apoptotic A549 cells stimulated with 0 (control), 2.5 or 5 μM **10b** or **10d**, for 24 h. The lower-right (annexin V + PI- cells) and the upper-right (annexin V + PI + cells) quadrants show early and late apoptotic cells, and the lower-left (annexin V-PI- cells) and the upper-left (annexin V-PI- cells) quadrants represent viable and necrotic cells, respectively. (B) Quantification of annexin V-positive apoptotic cells upon stimulation with **10b** or **10d**. The values are the means ± SEM of three different experiments. *p < 0.05 and *p < 0.01 indicate significant differences compared with control.

Furthermore, the pro-apoptotic effect of compounds **10b** and **10d** was also investigated on A549 cells using the Annexin V-FITC/PI double staining. The proportion of annexin V-positive apoptotic cells increased gradually from 5.4% at control to 16.5% at 5 μM **10b** (Figure 7(A,B)) and from 6.3% at control to 28.5% at 5 μM **10d**. These data indicate that the synthesised thiazolyl-pyrazoline derivatives treatment provokes the induction of apoptosis in A549 cells.

### 2.3. Molecular docking

**Figure 8.**
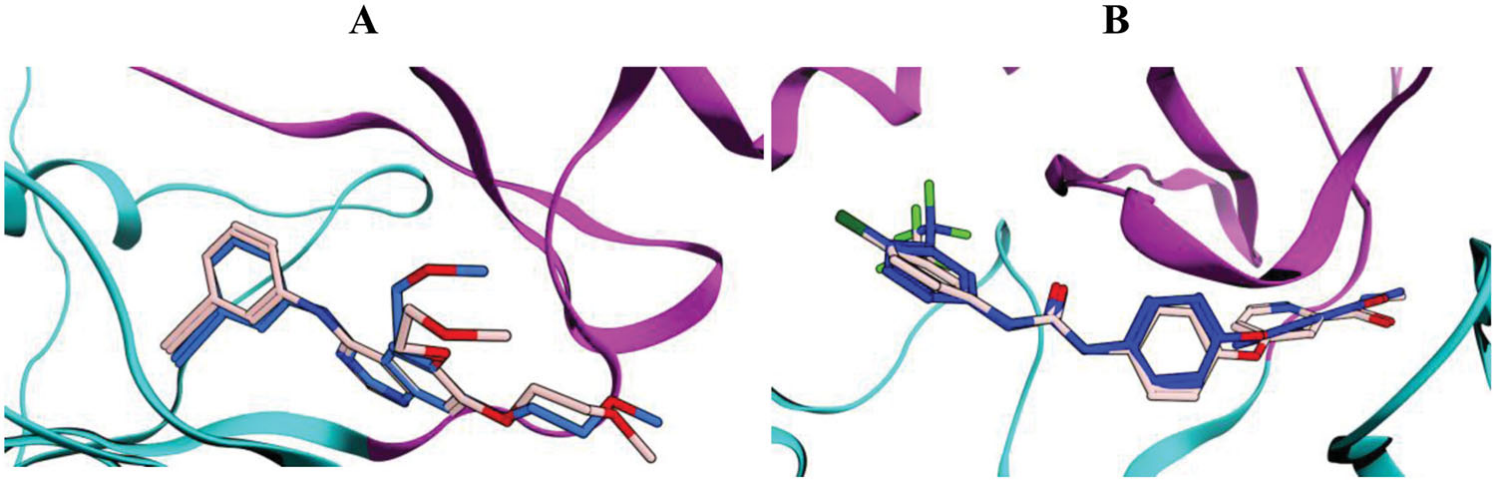
Superimposition of the co-crystallised (blue) and the docking pose (pink) of erlotinib in the EGFR (A) and sorafenib in the VEGFR-2 (B) binding sites.

In view of the promising kinase inhibitory activity of the thiazolyl-pyrazoline derivatives, herein reported, a molecular docking study was carried out for the most potent derivatives **10b** and **10d** to provide insights for their potential binding interactions within the examined kinases binding sites. The molecular operating environment (MOE) 2019.02 was implemented in the conduction, analysis and visualisation of the entire docking studies. The 3D structural co-ordinates of EGFR and VEGFR-2 were downloaded from the protein databank PDB IDs 1M17 and 4ASD, respectively. Both the selected PDB IDs have excellent resolution of 2.6 Å and 2.03 for 1M17 and 4ASD, respectively. In addition, 1M17 contains EGFR in complex with potent inhibitor erlotinib, while 4ASD contains VEGFR-2 in complex with the potent inhibitor sorafenib, making them optimum choice for the docking studies. Pose retrieval step of the co-crystallised ligands resulted in values of 0.85 and 0.63 Å between the docked and the co-crystallised poses for erlotinib and sorafenib, respectively, indicating a valid docking protocol (Figure 8).

Moreover, the previous step resulted in an energy scores (*S*) of −11.7 and −14.9 kcal/mol, for erlotinib and sorafenib, respectively. The retrieved docking scores of the co-crystalized ligands were used as comparative means to benchmark the docking score values of compounds **10b** and **10d**.

#### 2.3.1. Docking of compounds 10b and 10d into EGFR active site

**Figure 9.**
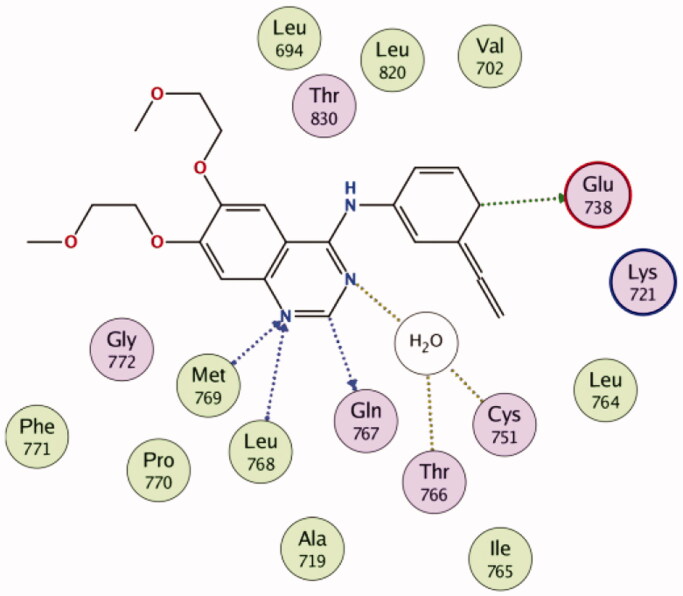
2D diagram for the binding of erlotinib in the EGFR active site (PDB: 1M17).

Visual inspection of erlotinib binding with EGFR active site, revealed the formation of two hydrogen bonds with the key residues Leu768 and Met769, in addition, two carbon hydrogen bonds were noticed with Glu738 and Gln767. Through a water molecule, erlotinib was able also to interact with Cys751 and Thr766 (Figure 9).

**Figure 10.**
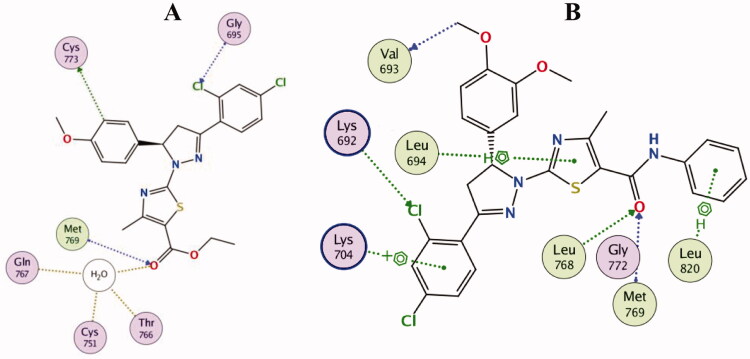
2D diagram for the binding interactions of compound **10b** (A) and compound **10d** (B) in the EGFR active site (PDB: 1M17).

Docking simulations for thiazolyl-pyrazoline derivatives **10b** and **10d** showed that they fit well into the EGFR active site with good docking scores (−12.9 and −14.1 kcal/mol, respectively) comparable to that of erlotinib (−11.7 kcal/mol). The general binding patterns of compounds **10b** and **10d** are consistent with crystallographic binding mode of erlotinib within the EGFR-TK active site (PDB: 1M17), Figure 10.

**Figure 11.**
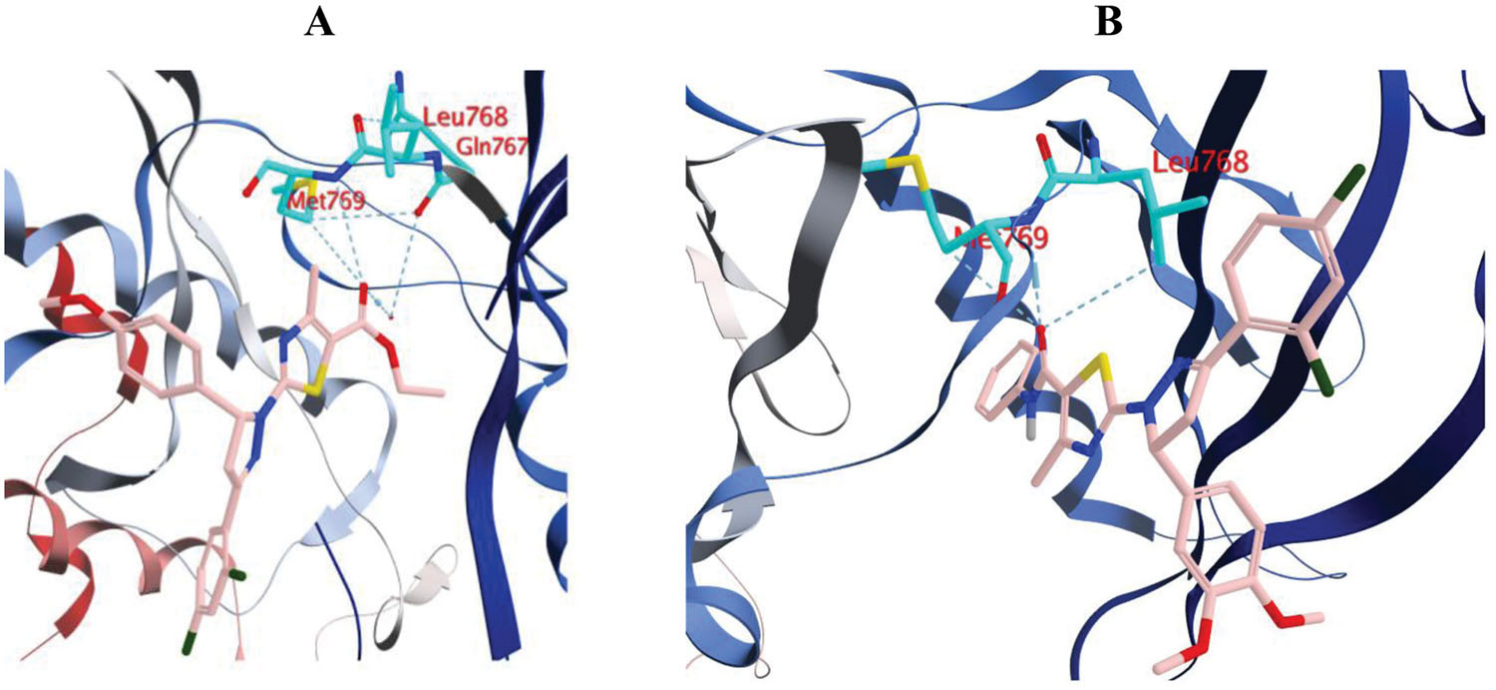
3D diagram of compounds **10b** (A) and **10d** (B) showing their binding interaction with the EGFR active site (PDB: 1M17).

For instance, the carbonyl group in the amide functionality of compound **10d** was engaged in two hydrogen bonds with the key residues Leu768 and Met769. Similarly, the carbonyl group in the ester functionality of compound **10b** was engaged in hydrogen bond interaction with Met769, in addition to three hydrogen bonds with Gln767, Cys751 and Thr766 through a water molecule (Figures 10 and 11).

Compounds **10b** and **10d** formed a carbon hydrogen bond with Cys773 and Val693, respectively. The *ortho*-chloride atom of compound **10b** formed a halogen interaction with Gly695, while the *ortho*-chloride atom of compound **10d** formed a halogen interaction with Lys692. On the other hand, only compound **10d** was engaged in arene bond interactions with Leu694, Lys704 and Leu802, which explains the superior activity and docking score of compound **10d** over compound **10b**. Also, it highlights the important role of the additional phenyl ring attached to the amide linker in compound **10d** in EGFR inhibition (Figures 10 and 11).

#### 2.3.2. Docking of compounds 10b and 10d into VEGFR-2 active site

**Figure 12.**
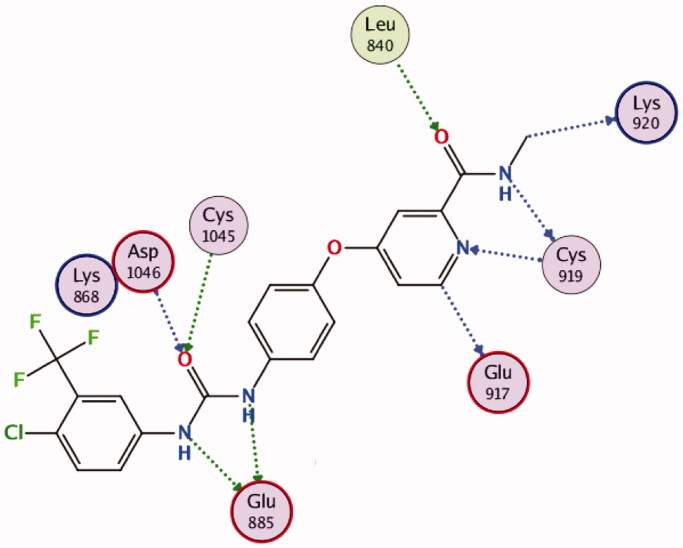
2D diagram for the binding interactions of sorafenib in the VEGFR-2 active site (PDB: 4ASD).

The efficient VEGFR-2 inhibitory activity of compounds **10b** and **10d** were illustrated by their good docking scores (−13.7 and −14.4 kcal/mol) comparable to that of sorafenib (−14.9 kcal/mol). As depicted in Figure 12, sorafenib exerts its VEGFR-2 inhibition activity through formation of hydrogen bonds with the essential residues; Glu885, Cys1045, Asp1046 and Cys919, in addition to other multiple interactions.

**Figure 13.**
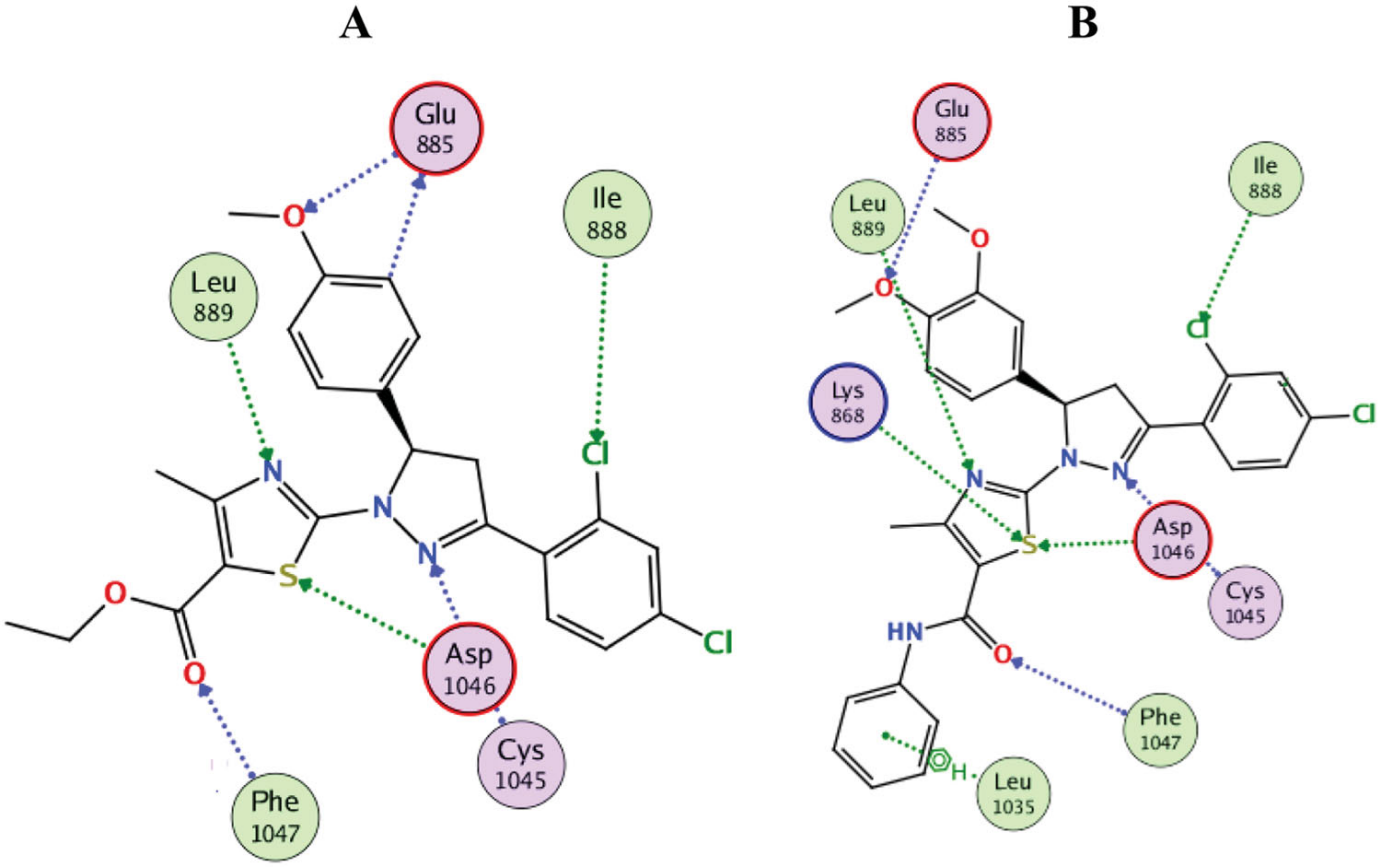
2D diagram of compounds **10b** (A) and **10d** (B) showing their binding interaction with the VEGFR-2 active site (PDB: 4ASD).

**Figure 14.**
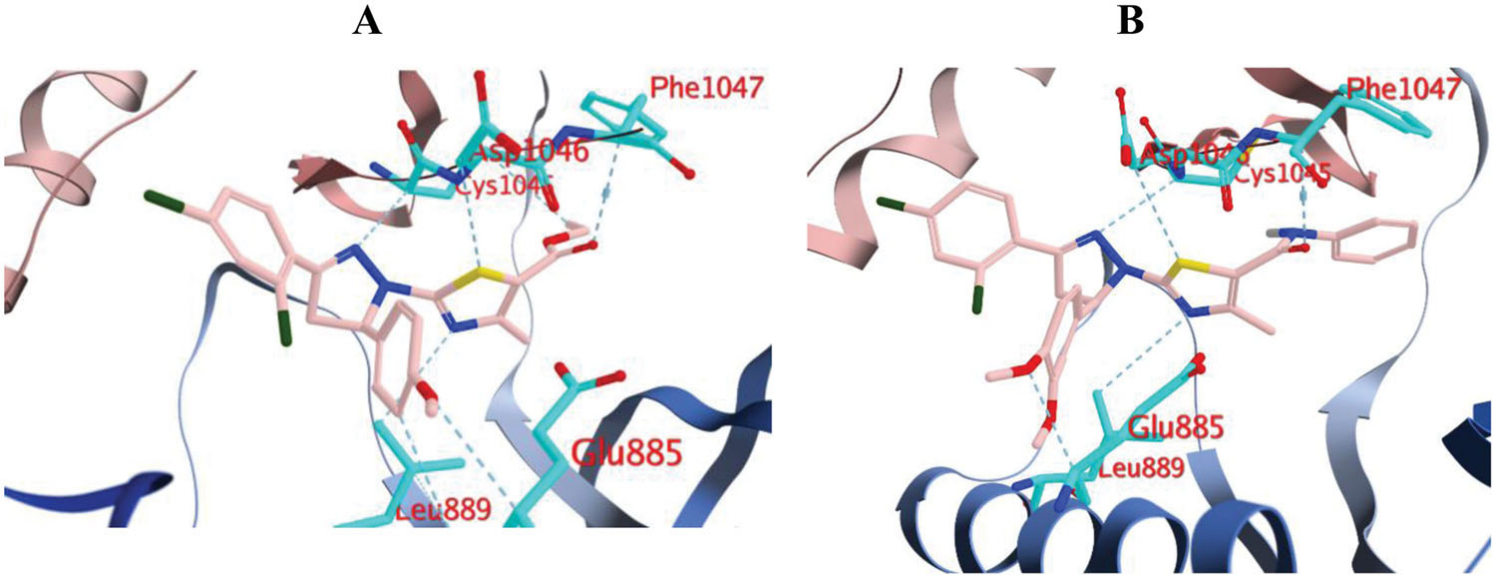
3D diagram of compounds **10b** (A) and **10d** (B) showing their binding interaction with the VEGFR-2 active site (PDB: 4ASD).

Interestingly, both compounds **10b** and **10d** were proven to maintain multiple essential interactions upon docking into VEGFR-2 active site (Figures 13 and 14). For instance, compound **10b** was engaged in hydrogen bond interactions with Leu889 and Asp1046 *via* the nitrogen and sulphur atoms of the thiazole ring, respectively. The nitrogen of pyrazoline ring formed two hydrogen bonds with Cys1045, in addition, the carbonyl group in the ester functional contributed to a hydrogen bond with Phe1047. The *ortho*-chloride and the *para*-methoxy substituents contributed to bonding interactions with Ile888 and Glu885, respectively. In a similar manner, all the previous interactions were achieved by compound **10d** in addition to an extra hydrogen bond interaction with Lys868, in addition to an arene interaction with Leu1035 (Figures 13 and 14).

## 3. Conclusion

In conclusion, this study reports the facile synthesis of potent anticancer series of thiazolyl-pyrazoline derivatives acting as dual EGFR/VEGFR-2 inhibitors with significant proapoptotic properties. All the synthesised thiazolyl-pyrazolines have been screened for their inhibitory activities against EGFR and VEGFR-2 kinases and for their cytotoxic effect against nine cell lines derived from five tumour subpanels. Compounds **10b** and **10d** displayed potent and selective inhibitory activity towards EGFR-TK (IC_50_ = 40.7 ± 1.0 and 32.5 ± 2.2 nM, respectively) and VEGFR-2 (IC_50_ = 78.4 ± 1.5 and 43.0 ± 2.4 nM, respectively). Furthermore, compounds **10b** and **10d** showed a promising anticancer activity against non-small lung cell lines as they were the most potent derivatives with IC_50_ values equal 4.2 and 2.9 µM against A549 cells, and equal 4.8 and 3.8 µM against H441 cells, respectively. Moreover, our results indicated that **10b** and **10d** were much effective towards EGFR-mutated NSCLC cell lines (NCI-H1650 and NCI-H1975 cells) than gefitinib. Finally, compounds **10b** and **10d** induce G_2_/M cell cycle arrest and apoptosis and inhibit migration in A549 cancerous cells. Furthermore, the molecular docking study explored the binding mode and possible different interactions between the target compounds and the active sites of EGFR and VEGFR-2 enzymes. Accordingly, thiazolyl-pyrazoline scaffold can be considered as promising scaffold for further development of more potent dual EGFR and VEGFR-2 inhibitors.

## 4. Experimental

### 4.1. Chemistry

#### 4.1.1. General

Melting points were measured with a Stuart melting point apparatus (Stuart Scientific, Redhill, UK) and were uncorrected. Infra-red (IR) spectra were recorded on FT-IR spectrometer using KBr discs (Perkin Elmer, Waltham, MA). Mass spectra (MS) were performed on a Varian electron impact EI mass spectrometer (EI-MS) at 70 eV at Regional Centre for Mycology and Biotechnology, Al-Azhar University utilising Thermo Scientific ISQ LT mass spectrometer (Thermo Fisher Scientific Inc., Waltham, MA). NMR Spectra were recorded on a Bruker NMR spectrometer (Bruker Biospin GmbH, Rheinstetten, Germany) at Center for Drug Discovery Research and Development, Faculty of Pharmacy, Ain Shams University. ^1^H spectra were run at 400 MHz and ^13^C spectra were run at 100 MHz in deuterated dimethyl sulfoxide (DMSO-d_6_) or chloroform C*D*Cl_3_. Chemical shifts are expressed in *δ* values (*ppm*) using the solvent peak as internal standard. All coupling constant (*J*) values are given in hertz. The abbreviations used are as follows: s, singlet; d, doublet; m, multiplet. Elemental analyses were carried out at the Regional Centre for Mycology and Biotechnology, Al-Azhar University utilising Thermo Scientific Flash 2000 elemental analyser (Thermo Fisher Scientific Inc., Waltham, MA). Analytical thin-layer chromatography (TLC) was employed routinely to follow the course of reactions and to check the purity of products using aluminium sheets pre coated with silica gel (Kieselgel, F254, pore size 60 Å, Merck, Darmstadt, Germany) and observed under a UV lamp (short-wavelength, 254 nm). All reagents and solvents were purified and dried by standard techniques.

#### 4.1.2. General procedure for preparation of 1-(2,4-dichlorophenyl)-3-(aryl) prop-2-en-1-one 3a, b

To a solution of 2,4-dichloroacetophenone **1** (1.76 g, 9.3 mmol) and an appropriate aldehyde derivative **2a, b** (9.3 mmol) in absolute ethanol (40 ml), 10% sodium hydroxide solution (15 ml) was added portion wise. The mixture was stirred for 4 h at room temperature. The separated precipitate was filtered, washed with water, dried and crystallised from ethanol to afford compounds **3a, b**, respectively.

##### 4.1.2.1. 1-(2,4-Dichlorophenyl)-3-(4-methoxyphenyl)prop-2-en-1-one (3a)

Yellow powder in 87% yield, m.p. 102–103 °C (reported m.p. 106–107 °C^60^) IR (KBr, *ν* cm^−1^): 1653 (C=O); ^1^H NMR (DMSO-d_6_, 400 MHz) *δ ppm*: 3.81 (s, 3H, OCH_3_), 6.99 (d, *J* = 7.6 Hz, 2H, Ar-H), 7.11 (d, *J* = 16.8 Hz, 1H, olefinic H), 7.38 (d, *J* = 15.2 Hz, 1H, olefinic H), 7.58 (s, 2H, Ar-H), 7.72–7.76 (m, 3H, Ar-H); MS (EI) *m/z* (%): 307.28 (M^+^, 9.98), 131.17 (100); Anal. Calcd. for C_16_H_12_Cl_2_O_2_ (307.17): C, 62.56; H, 3.94; Found; C, 62.50; H, 3.97%.

##### 4.1.2.2. 1-(2,4-Dichlorophenyl)-3-(3,4-dimethoxyphenyl)prop-2-en-1-one (3b)

Yellow powder in 96% yield, m.p. 138–139 °C (reported m.p. 134–136 °C^61^) IR (KBr, *ν* cm^−1^): 1657 (C=O); ^1^H NMR (DMSO-d_6_, 400 MHz) *δ ppm:* 3.81 (s, 6H, 2 (OCH_3_)), 7.01 (d, *J* = 8.4 Hz, 1H, Ar-H), 7.16 (d, *J* = 16 Hz, 1H, olefinic H), 7.31–7.41 (m, 3H, Ar-H + olefinic H), 7.58 (s, 2H, Ar-H), 7.78 (s, 1H, Ar-H); MS (EI) *m/z* (%): 337.14 (M^+^, 7.37), 83.13 (100); Anal. Calcd. for C_17_H_14_Cl_2_O_3_ (337.2): C, 60.55; H, 4.18; Found; C, 60.62; H, 4.13%.

#### 4.1.3. General procedure for preparation of carbothioamide derivatives 5a, b

To a mixture of **3a, b** (1 mmol) and thiosemicarbazide **4** (1 mmol) in absolute ethanol (50 ml), sodium hydroxide (0.29 g, 7 mmol) was added. The reaction mixture was heated under reflux with stirring for 6 h. After cooling, the formed product was filtered, washed with ethanol, dried and crystallised from ethanol to give the corresponding pyrazoline derivatives **5a, b**, respectively.

##### 4.1.3.1. 3-(2,4-Dichlorophenyl)-5-(4-methoxyphenyl)-4,5-dihydro-1H-pyrazole-1-carbothioamide (5a)

Yellow powder in 70% yield, m.p. 168–169 °C (reported m.p. 175–178 °C^62^) IR (KBr, *ν* cm^−1^): 3418 and 3247 (NH_2_), 1599 (C = N), 1236 and 828 (C = S); ^1^H NMR (DMSO-d_6_, 400 MHz) *δ* ppm: 3.18 (dd, *J* = 2.8, 16 Hz, 1H, H_A_), 3.73 (s, 3H, OCH_3_), 4.03 (dd, *J* = 12.6, 17.2 Hz, 1H, H_M_), 5.87 (dd, *J* = 3.2, 12 Hz, 1H, H_X_), 6.88 (d, *J* = 10.4 Hz, 2H, Ar-H), 7.08 (d, *J* = 8.4 Hz, 2H, Ar-H), 7.52 (dd, *J* = 2.4, 8.4 Hz, 1H, Ar-H), 7.71 (d, *J* = 1.6 Hz, 1H, Ar-H), 7.75 (s, 1H, NH, D_2_O exchangeable), 8.00 (d, *J* = 8 Hz, 1H, Ar-H), 8.13 (s, 1H, NH, D_2_O exchangeable); ^13 ^C NMR (DMSO-d_6_, 100 MHz) *δ* ppm: 45.7 (CH_2_ pyrazoline), 55.5 (OCH_3_), 63.0 (CH pyrazoline), 114.3 (2 C), 127.1 (2 C), 128.1, 129.3, 130.7, 132.7, 133.4, 135.2, 135.7, 153.3, 158.7, 177.0 (C=S); MS (EI) *m/z* (%): 380.16 (M^+^, 28.54), 182.72 (100), Anal. Calcd. for C_17_H_15_Cl_2_N_3_OS (380.29): C, 53.69; H, 3.98; N, 11.05; Found; C, 53.74; H, 3.95; N, 11.13%.

##### 4.1.3.2. 3-(2,4-Dichlorophenyl)-5-(3,4-dimethoxyphenyl)-4,5-dihydro-1H-pyrazole-1-carbothioamide (5b)

Light yellow powder in 98% yield, m.p. 198–200 °C; IR (KBr, *ν* cm^−1^): 3444 and 3322 (NH_2_), 1581 (C=N), 1254 and 833 (C=S); ^1^H NMR (DMSO-d_6_, 400 MHz) *δ* ppm: 3.19–3.23 (m, 1H, H_A_), 3.72 (s, 6H, 2OCH_3_), 3.99–4.07 (dd, *J* = 17.6, 11.6 Hz, 1H, H_M_), 5.86–5.89 (m, 1H, H_X_), 6.65 (d, *J* = 8.8 Hz, 1H, Ar-H), 6.80 (s, 1H, Ar-H), 6.88 (d, *J* = 7.6 Hz, 1H, Ar-H), 7.52 (d, *J* = 9.6 Hz, 1H, Ar-H), 7.71 (s, 1H, Ar-H), 7.76 (s, 1H, NH, D_2_O exchangeable), 7.96 (d, *J* = 9.6 Hz, 1H, Ar-H), 8.13 (s, 1H, NH, D_2_O exchangeable); ^13 ^C NMR (DMSO-d_6_, 100 MHz) *δ* ppm: 45.7 (CH_2_ pyrazoline), 55.9 (OCH_3_), 56.0 (OCH_3_), 63.3 (CH pyrazoline), 110.0, 112.3, 117.6, 128.1, 129.4, 130.7, 132.7, 133.4, 135.5, 135.7, 148.3, 149.2, 153.5 (C=N), 177.1 (C=S); MS (EI) *m/z* (%): 410 (M^+^, 24.38), 216.30 (100); Anal. Calculated for C_18_H_17_Cl_2_N_3_O_2_S (410): C, 52.69; H, 4.18; N, 10.24; Found; C, 52.73; H, 4.11; N, 10.29%.

#### 4.1.4. General procedure for preparation of compounds 7a–7d

A mixture of carbothioamides **5a, b** (1 mmol) and the appropriate 1-aryl-2-bromoethanone **6a, b** (1.1 mmol) in absolute ethanol (20 ml) was heated under reflux for 4 h. After cooling, the formed precipitate was filtered and crystallised from ethanol to afford the corresponding compounds **7a–*7*d.**

##### 4.1.4.1. 2-(3-(2,4-Dichlorophenyl)-5-(4-methoxyphenyl)-4,5-dihydro-1H-pyrazol-1-yl)-4-phenylthiazole (7a)

Yellow powder in 55% yield, m.p. 72–75 °C; IR (KBr, *ν* cm^−1^): 1583 (C=N); ^1^H NMR (DMSO-d_6_, 400 MHz) *δ* ppm: 3.38–3.44 (m, 1H, H_A_), 3.70 (s, 3H, OCH_3_), 4.05–4.12 (m, 1H, H_M_), 5.64 (s, 1H, H_X_), 6.91 (d, *J* = 8.8, 2H, Ar-H), 7.26–7.35 (m, 6H, 5 Ar-H and thiazole H) , 7.51 (d, *J* = 12 Hz, 1H, Ar-H), 7.69–7.79 (m, 4H, Ar-H); MS (EI) *m/z* (%): 480 (M^+^, 16.80), 76.10 (100); Anal. Calcd. for C_25_H_19_Cl_2_N_3_OS (480): C, 62.50; H, 3.99; N, 8.75; Found; C, 62.41; H, 4.17; N, 8.82%.

##### 4.1.4.2. 4-(4-Chlorophenyl)-2-(3-(2,4-dichlorophenyl)-5-(4-methoxyphenyl)-4,5-dihydro-1H-pyrazol-1-yl)thiazole (7b)

Yellow powder in 60% yield, m.p. 140–141 °C; IR (KBr, *ν* cm^−1^): 1583 (C=N); ^1^H NMR (DMSO-d_6_, 400 MHz) *δ* ppm: 3.42–3.46 (m, 1H, H_A_), 3.72 (s, 3H, OCH_3_), 4.10 (dd, *J* = 17.6, 12 Hz, 1H, H_M_), 5.63 (dd, *J* = 14, 5.2 Hz, 1H, H_X_), 6.91 (d, *J* = 8.8 Hz, 2H, Ar-H), 7.34–7.42 (m, 5H, Ar-H and thiazole H), 7.53 (d, *J* = 10.4 Hz, 1H, Ar-H), 7.72–7.80 (m, 4H, Ar-H); ^13 ^C NMR (DMSO-d_6_, 100 MHz) *δ* ppm: 45.8 (CH_2_ pyrazoline), 55.5 (OCH_3_), 64.3 (CH pyrazoline), 106.0, 114.4 (2 C), 127.7 (2 C), 128.1, 128.5 (2 C), 129.0 (2 C), 129.2, 130.9, 132.2, 132.5, 132.9, 133.7, 133.7, 135.0, 149.8, 150.8, 159.2, 164.8; MS (EI) *m/z* (%): 514 (M^+^, 10.28), 89.34 (100); Anal. Calcd. for C_25_H_18_Cl_3_N_3_OS (514): C, 58.32; H, 3.52; N, 8.16; Found; C, 58.44; H, 3.76; N, 8.41%.

##### 4.1.4.3. 2-(3-(2,4-Dichlorophenyl)-5-(3,4-dimethoxyphenyl)-4,5-dihydro-1H-pyrazol-1-yl)-4-phenylthiazole (7c)

Yellow powder in 69% yield, m.p. 120–122 °C; IR (KBr, *ν* cm^−1^): 1584 (C=N); ^1^H NMR (DMSO-d_6_, 400 MHz) *δ* ppm: 3.50 (dd, *J* = 17.6, 8 Hz, 1H, H_A_), 3.72 (s, 3H, OCH_3_), 3.78 (s, 3H, OCH_3_), 4.12 (dd, *J* = 17.6, 12.4 Hz, 1H, H_M_), 5.64 (dd, *J* = 12.4, 7.2 Hz, 1H, H_X_), 6.92–7.09 (m, 3H, Ar-H), 7.26–7.38 (m, 4H, 3 Ar-H, thiazole H), 7.56 (dd, *J* = 9.2, 2.8 Hz, 1H, Ar-H), 7.76–7.78 (m, 3H, Ar-H), 7.82 (d, *J* = 8.8 Hz, 1H, Ar-H); ^13 ^C NMR (DMSO-d_6_, 100 MHz) *δ* ppm: 45.8 (CH_2_ pyrazoline), 55.9 (2OCH_3_), 64.7 (CH pyrazoline), 105.2, 111.6, 112.3, 119.2, 126.0 (2 C), 128.1, 128.2, 129.0, 129.4 (2 C), 130.8, 132.3, 132.9, 134.2, 134.9, 135.0, 148.8, 149.0, 150.8, 151.0, 164.77; MS (EI) *m/z* (%): 510 (M^+^, 15.80), 96.39 (100); Anal. Calcd. for C_26_H_21_Cl_2_N_3_O_2_S (510): C, 61.18; H, 4.15; N, 8.23; Found; C, 61.02; H, 4.39; N, 8.47%.

##### 4.1.4.4. 4-(4-Chlorophenyl)-2-(3-(2,4-dichlorophenyl)-5-(3,4-dimethoxyphenyl)-4,5-dihydro-1H-pyrazol-1-yl)thiazole (7d)

Yellow powder in 79% yield, m.p. 158–159 °C; IR (KBr, *ν* cm^−1^): 1584 (C=N); ^1^H NMR (DMSO-d_6_, 400 MHz) *δ* ppm: 3.48 (dd, *J* = 15.2, 8 Hz, 1H, H_A_), 3.72 (s, 3H, OCH_3_), 3.76 (s, 3H, OCH_3_), 4.11 (dd, *J* = 14.8, 9.6 Hz, 1H, H_M_), 5.62 (dd, *J* = 15.2, 9.2 Hz, 1H, H_X_), 6.93 (s, 2H, Ar-H), 7.06 (s, 1H, Ar-H), 7.40–7.43 (m, 3H, 2Ar-H and thiazole H), 7.54 (d, *J* = 7.6 Hz, 1H, Ar-H), 7.75–7.81 (m, 4H, Ar-H); MS (EI) *m/z* (%): 544 (M^+^, 21.11), 218.49 (100); Anal. Calcd. for C_26_H_20_Cl_3_N_3_O_2_S (544): C, 57.31; H, 3.70; N, 7.71; Found; C, 57.53; H, 3.86; N, 7.89%.

#### 4.1.5. General procedure for preparation of compounds 10a–10d

A mixture of carbothioamides **5a**, **b** (1 mmol) and 3-chloropentane-2,4-diones **9a–9c** (1.1 mmol) in absolute ethanol (30 ml) was heated under reflux for 4 h. After cooling, the formed precipitate was filtered and crystallised from ethanol to afford the corresponding compounds **10a–*10*d.**

##### 4.1.5.1. 1-(2-(3-(2,4-Dichlorophenyl)-5-(4-methoxyphenyl)-4,5-dihydro-1H-pyrazol-1-yl)-4-methylthiazol-5-yl)ethan-1-one (10a)

Yellow powder in 72% yield, m.p. 205–207 °C; IR (KBr, *ν* cm^−1^): 1647 (C=O); ^1^H NMR (DMSO-d_6_, 400 MHz) *δ* ppm: 2.38 (s, 3H, CH_3_), 2.40 (s, 3H, CH_3_), 3.73 (s, 3H, OCH_3_), 4.15 (dd, *J* = 18.8, 11.6 Hz, 1H, H_M_) 5.70 (dd, *J* = 11.6, 5.6 Hz, 1H, H_X_), 6.90 (d, *J* = 10.4 Hz, 2H, Ar-H), 7.21 (d, *J* = 8.8 Hz, 2H, Ar-H), 7.55 (d, *J* = 8.8 Hz, 1H, Ar-H), 7.77 (s, 1H, Ar-H), 7.81 (d, *J* = 9.6 Hz, 1H, Ar-H); ^13^C NMR (DMSO-d_6_, 100 MHz) *δ* ppm: 19.1 (CH_3_), 30.1 (CH_3_), 46.1 (CH_2_ pyrazoline), 55.5 (OCH_3_), 63.1 (CH pyrazoline), 114.6 (2 C), 124.6, 127.8, 128.2, 128.8, 131.0, 132.5, 133.2, 133.3, 135.5, 153.4, 157.5, 159.2, 165.2 (aromatic carbons), 189.7 (C=O); MS (EI) *m/z* (%): 460 (M^+^, 15.52), 153.75 (100); Anal. Calcd. for C_22_H_19_Cl_2_N_3_O_2_S (460): C, 57.40; H, 4.16; N, 9.13; Found; C, 57.31; H, 4.30; N, 9.25%.

##### 4.1.5.2. Ethyl-2-(3-(2,4-dichlorophenyl)-5-(4-methoxyphenyl)-4,5-dihydro-1H-pyrazol-1-yl)-4-methylthiazole-5-carboxylate (10b)

Yellow powder in 63% yield, m.p. 192–195 °C; IR (KBr, *ν* cm^−1^): 1669 (C=O); ^1^H NMR (C*D*Cl_3_, 400 MHz) *δ* ppm: 1.35 (t, *J* = 7.6 Hz, 3H, CH_3_), 2.54 (s, 3H, CH_3_), 3.53 (dd, *J* = 18.4, 8 Hz, 1H, H_A_), 3.81 (s, 3H, OCH_3_), 4.11 (dd, *J* = 20.4, 9.2 Hz, 1H, H_M_), 4.29 (q, *J* = 7.6 Hz, 2H, CH_2_), 5.90 (s, 1H, H_X_), 6.88 (d, *J* = 8.4 Hz, 2H, Ar-H), 7.27–7.29 (m, 2H, Ar-H), 7.34 (dd, *J* = 9.2, 3.6 Hz, 1H, Ar-H), 7.48 (d, *J* = 2.4 Hz, 1H, Ar-H), 7.85 (d, *J* = 7.6 Hz, 1H, Ar-H); ^13 ^C NMR (DMSO-d_6_, 100 MHz) *δ* ppm: 14.7 (CH_3_), 17.7 (CH_3_), 46.3 (CH_2_ pyrazoline), 55.5 (OCH_3_), 60.7 (CH_2_), 63.2 (CH pyrazoline), 111.4, 114.6, 127.8 (2 C), 128.2, 128.8, 131.0, 132.5, 133.1, 133.3, 135.5, 153.2, 159.2, 159.35, 162.2 (aromatic carbons), 165.3 (C=O); MS (EI) *m/z* (%): 490 (M^+^, 10.41), 55.10 (100); Anal. Calcd. for C_23_H_21_Cl_2_N_3_O_3_S (490): C, 56.33; H, 4.32; N, 8.57; Found; C, 56.60; H, 4.47; N, 8.78%.

##### 4.1.5.3. 2-(3-(2,4-Dichlorophenyl)-5-(4-methoxyphenyl)-4,5-dihydro-1H-pyrazol-1-yl)-4-methyl-N-phenylthiazole-5-carboxamide (10c)

White powder in 74% yield, m.p. 195–197 °C; IR (KBr, *ν* cm^−1^): 3257 (NH), 1637 (C=O); ^1^H NMR (DMSO-d_6_, 400 MHz) *δ* ppm: 2.36 (s, 3H, CH_3_), 3.35–3.40 (m, 1H, H_A_), 3.71 (s, 3H, OCH_3_), 4.15 (dd, *J* = 17.6, 12 Hz, 1H, H_M_) 5.68–5.71 (m, 1H, H_X_), 6.92 (d, *J* = 8.8 Hz, 2H, Ar-H), 7.06 (t, *J* = 8.8 Hz, 1H, Ar-H), 7.23 (d, *J* = 8.8 Hz, 2H, Ar-H), 7.30 (t, *J* = 8.8 Hz, 2H, Ar-H), 7.55 (d, *J* = 10.4 Hz, 1H, Ar-H), 7.62 (d, *J* = 8.8 Hz, 2H, Ar-H), 7.76 (s, 1H, Ar-H), 7.80 (d, *J* = 9.6 Hz, 1H, Ar-H), 9.72 (s, 1H, NH, D_2_O exchangeable); MS (EI) *m/z* (%): 537 (M^+^, 36.86), 245.37 (100); Anal. Calcd. for C_27_H_22_Cl_2_N_4_O_2_S (537): C, 60.34; H, 4.13; N, 10.42; Found; C, 60.49; H, 4.22; N, 10.68%.

##### 4.1.5.4. 2-(3-(2,4-Dichlorophenyl)-5-(3,4-dimethoxyphenyl)-4,5-dihydro-1H-pyrazol-1-yl)-4-methyl-N-phenylthiazole-5-carboxamide (10d)

Buff powder in 50% yield, m.p. 166–167 °C; IR (KBr, *ν* cm^−1^): 3262 (NH), 1637 (C=O); ^1^H NMR (DMSO-d_6_, 400 MHz) *δ* ppm: 2.38 (s, 3H, CH_3_), 3.72 (s, 3H, OCH_3_), 3.74 (s, 3H, OCH_3_), 4.14 (dd, *J* = 18.4, 12 Hz, 1H, H_M_), 5.68 (dd, *J* = 12, 5.6 Hz, 1H, H_X_), 6.78 (d, *J* = 7.6 Hz, 1H, Ar-H), 6.87–6.95 (m, 2H, Ar-H), 7.06 (t, *J* = 6.4 Hz, 1H, Ar-H), 7.30 (t, *J* = 7.2 Hz, 2H, Ar-H), 7.55 (d, *J* = 11.6 Hz, 1H, Ar-H), 7.63 (d, *J* = 7.2 Hz, 2H, Ar-H), 7.75–7.81 (m, 2H, Ar-H), 8.14 (s, 1H, NH, D_2_O exchangeable); ^13 ^C NMR (DMSO-d_6_, 100 MHz) *δ* ppm: 18.2 (CH_3_), 46.1 (CH_2_ pyrazoline), 56.0 (2OCH_3_), 63.7 (CH pyrazoline), 110.7, 112.3, 116.5, 118.3, 120.8, 124.0 (2 C), 128.2, 129.0 (2 C), 130.9, 132.4, 133.0, 133.7, 135.3, 139.5, 148.7, 149.2, 152.3, 154.6, 160.9 (aromatic carbons), 163.8 (C=O); MS (EI) *m/z* (%): 567 (M^+^, 34.07), 299.67 (100); Anal. Calcd. for C_28_H_24_Cl_2_N_4_O_3_S (567): C, 59.26; H, 4.26; N, 9.87; Found; C, 59.40; H, 4.38; N, 9.82%.

#### 4.1.6. General procedure for preparation of compounds 13a–13f

A mixture of carbothioamides **5a, b** (1 mmol) and 2-oxo-*N*-arylpropanehydrazonoyl chlorides **12a–12c** (1.1 mmol) in absolute ethanol (30 ml) was heated under reflux for 4 h. The produced product was filtered and washed with hot ethanol to give the corresponding compounds **13a–13f**.

##### 4.1.6.1. 2-(3-(2,4-Dichlorophenyl)-5-(4-methoxyphenyl)-4,5-dihydro-1H-pyrazol-1-yl)-4-methyl-5-(phenyldiazenyl)thiazole (13a)

Red powder in 50% yield, m.p. 168–170 °C; IR (KBr, *ν* cm^−1^): 1582 (N = N); ^1^H NMR (DMSO-d_6_, 400 MHz) *δ* ppm: 3.46–3.47 (m, 1H, H_A_), 3.74 (s, 3H, CH_3_), 4.19 (dd, *J* = 18, 12 Hz, 1H, H_M_), 5.81 (dd, *J* = 12.4, 5.6 Hz, 1H, H_X_), 6.93 (d, *J* = 6.8 Hz, 2H, Ar-H), 7.25 (d, *J* = 12 Hz, 2H, Ar-H), 7.38 (t, *J* = 6 Hz, 1H, Ar-H), 7.48 (t, *J* = 7.6 Hz, 2H, Ar-H), 7.56 (d, *J* = 7.6 Hz, 1H, Ar-H), 7.69 (d, *J* = 7.6, 2H, Ar-H), 7.78 (s, 1H, Ar-H), 7.85 (d, *J* = 9.6 Hz, 1H, Ar-H); ^13 ^C NMR (DMSO-d_6_, 100 MHz) *δ* ppm: 16.5 (CH_3_ of thiazole), 46.3 (CH_2_ pyrazoline), 55.6 (OCH_3_), 63.0 (CH pyrazoline), 114.7 (2 C), 122.1 (2 C), 127.8 (2 C), 128.2 (2 C), 128.6, 129.8, 131.1 (2 C), 132.7, 133.2, 133.3, 135.7, 141.4, 152.6, 154.3, 158.3, 159.3, 165.0; MS (EI) *m/z* (%): 524 (M^+^ + 2, 1.17), 522 (M^+^, 4.86), 40.16 (100); Anal. Calcd. for C_26_H_21_Cl_2_N_5_OS (522): C, 59.77; H, 4.05; N, 13.40; Found; C, 59.86; H, 4.13; N, 13.62%.

##### 4.1.6.2. 2-(3-(2,4-Dichlorophenyl)-5-(4-methoxyphenyl)-4,5-dihydro-1H-pyrazol-1-yl)-4-methyl-5-(p-tolyldiazenyl)thiazole (13b)

Red powder in 80% yield, m.p. 170–171 °C; IR (KBr, *ν* cm^−1^): 1582 (N=N); ^1^H NMR (C*D*Cl_3_, 400 MHz) *δ* ppm: 2.42 (s, 3H, CH_3_), 2.61 (s, 3H, CH_3_), 3.53 (dd, *J* = 18, 7.2 Hz, 1H, H_A_), 3.81 (s, 3H, OCH_3_), 4.12 (dd, *J* = 19.2, 9.6 Hz, 1H, H_M_), 5.77–5.80 (m, 1H, H_X_), 6.89 (d, *J* = 9.2 Hz, 2H, Ar-H), 7.23–7.29 (m, 4H, Ar-H), 7.32 (dd, *J* = 9.2, 2 Hz, 1H, Ar-H), 7.47 (d, *J* = 2.8 Hz, 1H, Ar-H), 7.67 (d, *J* = 6.4 Hz, 2H, Ar-H), 7.90 (d, *J* = 10 Hz, 1H, Ar-H); ^13 ^C NMR (C*D*Cl_3_, 100 MHz) *δ* ppm: 16.1 (CH_3_ of thiazole), 21.4 (CH_3_), 46.2 (CH_2_ pyrazoline), 55.3 (OCH_3_), 63.5 (CH pyrazoline), 114.3 (2 C), 122.0 (2 C), 127.3 (2 C), 127.5 (2 C), 128.7, 129.6, 130.8 (2 C), 131.4, 132.6, 133.5, 136.3, 139.4, 142.2, 150.9, 152.3, 159.4, 164.8; MS (EI) *m/z* (%): 536 (M^+^, 32.38), 110.11 (100); Anal. Calcd. for C_27_H_23_Cl_2_N_5_OS (536): C, 60.45; H, 4.32; N, 13.05; Found; C, 60.31; H, 4.49; N, 13.28%.

##### 4.1.6.3. 5-((4-Chlorophenyl)diazenyl)-2-(3-(2,4-dichlorophenyl)-5-(4-methoxyphenyl)-4,5-dihydro-1H-pyrazol-1-yl)-4-methylthiazole (13c)

Red powder in 80% yield, m.p. 170–171 °C; IR (KBr, *ν* cm^−1^): 1582 (N=N); ^1^H NMR (DMSO-d_6_, 400 MHz) *δ* ppm: 3.44 (dd, *J* = 16.8, 6.4 Hz, 1H, H_A_), 3.73 (s, 3H, CH_3_), 4.18 (dd, *J* = 16, 8 Hz, 1H, H_M_), 5.80 (dd, *J* = 9.6, 6 Hz, 1H, H_X_), 6.93 (d, *J* = 7.2 Hz, 2H, Ar-H), 7.24 (d, *J* = 9.6 Hz, 2H, Ar-H), 7.51 (d, *J* = 8.8 Hz, 2H, Ar-H), 7.55 (d, *J* = 8 Hz, 1H, Ar-H), 7.68 (d, *J* = 8 Hz, 2H, Ar-H), 7.77 (s, 1H, Ar-H), 7.84 (d, *J* = 7.6 Hz, 1H, Ar-H); ^13 ^C NMR (DMSO-d_6_, 100 MHz) *δ* ppm: 16.6 (CH_3_), 46.3 (CH_2_ pyrazoline), 55.6 (OCH_3_), 63.0 (CH pyrazoline), 114.7 (2 C), 120.2, 123.6 (2 C), 127.8, 128.3, 128.5, 129.8, 131.1, 132.7 (2 C), 133.1, 133.3, 133.8, 135.8, 141.3, 151.3, 154.7, 159.3, 159.4, 165.3; MS (EI) *m/z* (%): 556 (M^+^, 35.77), 311.62 (100); Anal. Calcd. for C_26_H_20_Cl_3_N_5_OS (556): C, 56.07; H, 3.62; N, 12.58; Found; C, 56.20; H, 3.88; N, 12.61%.

##### 4.1.6.4. 2-(3-(2,4-Dichlorophenyl)-5-(3,4-dimethoxyphenyl)-4,5-dihydro-1H-pyrazol-1-yl)-4-methyl-5-(phenyldiazenyl)thiazole (13d)

Reddish orange powder in 50% yield, m.p. 187–188 °C; IR (KBr, *ν* cm^−1^): 1587 (N=N); ^1^H NMR (DMSO-d_6_, 400 MHz) *δ* ppm: 3.44–3.50 (m, 1H, H_A_), 3.72 (s, 3H, OCH_3_), 3.75 (s, 3H, OCH_3_), 4.18 (dd, *J* = 17.2, 11.6 Hz, 1H, H_M_), 5.80 (dd, *J* = 11.2, 5.6 Hz, 1H, H_X_), 6.79 (d, *J* = 6.4 Hz, 1H, Ar-H), 6.91–6.96 (m, 2H, Ar-H), 7.37 (t, *J* = 7.6 Hz, 1H, Ar-H), 7.48 (t, *J* = 8.4 Hz, 2H, Ar-H), 7.55 (d, *J* = 8.8 Hz, 1H, Ar-H), 7.68 (d, *J* = 8.4 Hz, 2H, Ar-H), 7.76 (s, 1H, Ar-H), 7.83 (d, *J* = 7.6 Hz, 1H, Ar-H); ^13 ^C NMR (DMSO-d_6_, 100 MHz) *δ* ppm: 16.4 (CH_3_ of thiazole), 46.2 (CH_2_ pyrazoline), 55.9 (2OCH_3_), 63.3 (CH pyrazoline), 110.5 (2 C), 112.3, 118.1, 122.1 (2 C), 128.3 (2 C), 129.8, 131.0 (2 C), 132.6, 133.2 (2 C), 133.4, 135.8 (2 C), 148.8, 149.2 (2 C), 152.6, 165.1; MS (EI) *m/z* (%): 552 (M^+^, 15.69), 329.74 (100); Anal. Calcd. for C_27_H_23_Cl_2_N_5_O_2_S (552): C, 58.70; H, 4.20; N, 12.68; Found; C, 58.92; H, 3.37; N, 12.68%.

##### 4.1.6.5. 2-(3-(2,4-Dichlorophenyl)-5-(3,4-dimethoxyphenyl)-4,5-dihydro-1H-pyrazol-1-yl)-4-methyl-5-(p-tolyldiazenyl)thiazole (13e)

Reddish orange powder in 73% yield, m.p. 176–177 °C; IR (KBr, *ν* cm^−1^): 1589 (N=N); ^1^H NMR (C*D*Cl_3_, 400 MHz) *δ* ppm: 2.41 (s, 3H, CH_3_), 2.59 (s, 3H, CH_3_), 3.55 (dd, *J* = 18, 4 Hz, 1H, H_A_), 3.87 (s, 6H, 2OCH_3_), 4.10 (dd, *J* = 17.2, 13.2 Hz, 1H, H_M_), 5.73 (dd, *J* = 13.2, 4 Hz, 1H, H_X_), 6.83–6.86 (m, 3H, Ar-H), 7.22–7.34 (m, 3H, Ar-H), 7.46 (s, 1H, Ar-H), 7.66 (d, *J* = 9.6 Hz, 2H, Ar-H), 7.87 (d, *J* = 8.8 Hz, 1H, Ar-H); ^13 ^C NMR (C*D*Cl_3_, 100 MHz) *δ* ppm: 16.1 (CH_3_), 21.4 (CH_3_), 46.1 (CH_2_ pyrazoline), 55.9 (OCH_3_), 56.0 (OCH_3_), 63.7 (CH pyrazoline), 109.4, 111.4, 118.1, 122.0 (2 C), 127.5, 128.7, 129.6 (2 C), 130.8, 131.3, 133.0, 133.5, 136.3, 139.4, 148.8, 149.2, 150.9, 152.3, 156.5, 164.8; MS (EI) *m/z* (%): 566 (M^+^, 16.00), 46.07 (100); Anal. Calcd. for C_28_H_25_Cl_2_N_5_O_2_S (566): C, 59.36; H, 4.45; N, 12.36; Found; C, 59.46; H, 4.38; N, 12.59%.

##### 4.1.6.6. 5-((4-Chlorophenyl)diazenyl)-2-(3-(2,4-dichlorophenyl)-5-(3,4-dimethoxyphenyl)-4,5-dihydro-1H-pyrazol-1-yl)-4-methylthiazole (13f)

Orange powder in 70% yield, m.p. 190–191 °C; IR (KBr, *ν* cm^−1^): 1586 (N=N); ^1^H NMR (DMSO-d_6_, 400 MHz) *δ* ppm: 2.53 (s, 3H, CH_3_), 3.48 (dd, *J* = 17.6, 5.6 Hz, 1H, H_A_), 3.73 (s, 3H, OCH_3_), 3.75 (s, 3H, OCH_3_), 4.19 (dd, *J* = 19.6, 12.8 Hz, 1H, H_M_), 5.81 (dd, *J* = 12, 5.6 Hz, 1H, H_X_), 6.78 (d, *J* = 7.6 Hz, 1H, Ar-H), 6.92–6.96 (m, 2H, Ar-H), 7.53 (d, *J* = 9.2 Hz, 2H, Ar-H), 7.57 (d, *J* = 7.6 Hz, 1H, Ar-H), 7.71 (d, *J* = 9.2 Hz, 2H, Ar-H), 7.79 (d, *J* = 3.2 Hz, 1H, Ar-H), 7.85 (d, *J* = 10 Hz, 1H, Ar-H); MS (EI) *m/z* (%): 586 (M^+^, 55.20), 458.78 (100); Anal. Calcd. for C_27_H_22_Cl_3_N_5_O_2_S (586): C, 55.25; H, 3.78; N, 11.93; Found; C, 55.43; H, 3.89; N, 12.14%.

### 4.2. Biological evaluation

#### 4.2.1. Cell culture and reagents

Human leukaemia (K562 and KG-1a), breast (MCF-7, BT-549 and HCC70), lung (A549, H441, NCI-H1650 and NCI-H1975), colon (HCT116), liver (HepG2) cancer cell lines and WI-38 human lung fibroblast cell line were obtained from the American Type Culture Collection (ATCC; Manassas, VA) and were cultured in their suitable media containing 10% foetal bovine serum (FBS; Sigma-Aldrich) in a humidified atmosphere with 5% CO_2_ at 37 °C. Gefitinib and vandetanib were purchased from Sigma-Aldrich. All chemicals used in this study were analytical or cell-culture grade.

#### 4.2.2. Kinase inhibition assay

The EGFR kinase assay was carried out in 96-well plates coated with PGT (poly l-glutamic acid l-tyrosine, 4:1, Sigma Aldrich, St. Louis, MO) as previously described^63^. IC_50_ values were calculated using GraphPad Prism 5. Each experiment was carried out at least three times. The VEGFR-2 kinase assay was carried out in 96-well streptavidin coated plate using recombinant human VEGFR-2/KDR ELISA kit according to manufacturer’s instructions. Moreover, the IC_50_ values of **10b** and **10d** on 11 different kinases (TEK, SYK, EPHB2, ABL1, LCK, CLK1, ROCK1, PKC, AKT1, CDK1 and CDK5) were determined using Z'-LYTE® technology which is based on FRET (Invitrogen/Life Technologies).

#### 4.2.3. Western blot analysis

The protein extraction and quantification were done as previously described^64^.

#### 4.2.4. Anti-proliferative assay

Cytotoxicity was measured using the MTT assay. Human leukaemia (K562 and KG-1a), breast (MCF-7, BT-549 and HCC70), lung (A549, H441, NCI-H1650 and NCI-H1975), colon (HCT116), liver (HepG2) cancer cell lines and WI-38 human lung fibroblast cell line were plated at a density of 1 × 10^4^ cells per well in 96-well plates overnight and then treated with the compounds. After 24 h treatment, 20 μL of MTT solution (2 mg/mL in phosphate-buffered saline [PBS]) was added to each well and the cells were cultured for another 4 h at 37 °C. The medium was aspirated and 150 μL DMSO was added to solubilise MTT formazan crystals. The plates were then shaken, and the optical density was determined at 570 nm using an ELISA plate reader (Model 550, Bio-Rad, Hercules, CA). At least three independent experiments were performed. The IC_50_ values were calculated using GraphPad Prism 5 (Version 5.01, GraphPad Software, San Diego, CA).

#### 4.2.5. Cell cycle and apoptosis analyses

DNA content was measured using 7-aminoactinmycin D (7AAD) (Biotium, Inc., Hayward, CA) staining and is commonly used for cell cycle phase analysis as previously described^65^. Apoptosis was evaluated using the annexin V/propidium iodide (PI) staining kit (BioLegend, San Diego, CA) according to the manufacturer’s instructions and as previously described.

### 4.3. Molecular docking

In this work, all the docking studies were conducted using Molecular Operating Environment (MOE 2019.02) software^67^^,^^68^. The crystal structures of EGFR in complex with erlotinib and VEGFR-2 in complex with sorafenib were downloaded from the protein databank PDB IDs 1M17 and 4ASD, respectively. All the receptors and ligands were prepared using the default parameters of MOE software. The binding sites in the two targets were determined by selecting the pocket surrounding the binding domain of the co-crystallised ligands. Prior commencing the docking of compounds **10b** and **10d**, a pose-retrieval docking experiments for the X-ray coordinates of the co-crystallised ligands in their corresponding binding sites was carried out. The RMSD values between the co-crystallised pose and the docking pose from the previous step were 0.85 and 0.63 Å for erlotinib and sorafenib, respectively. After the positive indication on the docking validity, compounds **10b** and **10d** were docked into the two receptors using the same conditions. Finally, the results of the docking were visualised using the 2 D and 3 D interaction diagrams generated by MOE.

### 4.4. Scratch wound healing assay

A549 cells were seeded and incubated in 6-well cell culture plates till reaching confluence. Then, the confluent monolayers were scratched to form a “wound” using a sterile needle. The cells were treated with either vehicle as a control, **10b** or **10d** for 36 h. The images were recorded at 0 and 36 h to monitor the migration of cells into the wounded area using a light photomicroscope.

### 4.5. Statistical analysis

Data were presented as the means ± standard error of mean (SEM). Student’s *t*-test was performed to determine the statistical significance compared to the vehicle treated control, gefitinib or vandetanib. Statistical significance was defined as *p < 0.05, **p < 0.01 or ***p < 0.001. Data are representative of three independent experiments.

## Supplementary Material

Supplemental Material
