# Supplementary material for: Discovery of novel thiazolyl-pyrazolines as dual EGFR and VEGFR-2 inhibitors endowed with in vitro antitumor activity towards non-small lung cancer
Source: J Enzyme Inhib Med Chem. 2022 Aug 23;37(1):2265–82. doi: 10.1080/14756366.2022.2104841 (PMC9415638; doi:10.1080/14756366.2022.2104841)
Supplement: Supplemental Material [file IENZ_A_2104841_SM4599.pdf]

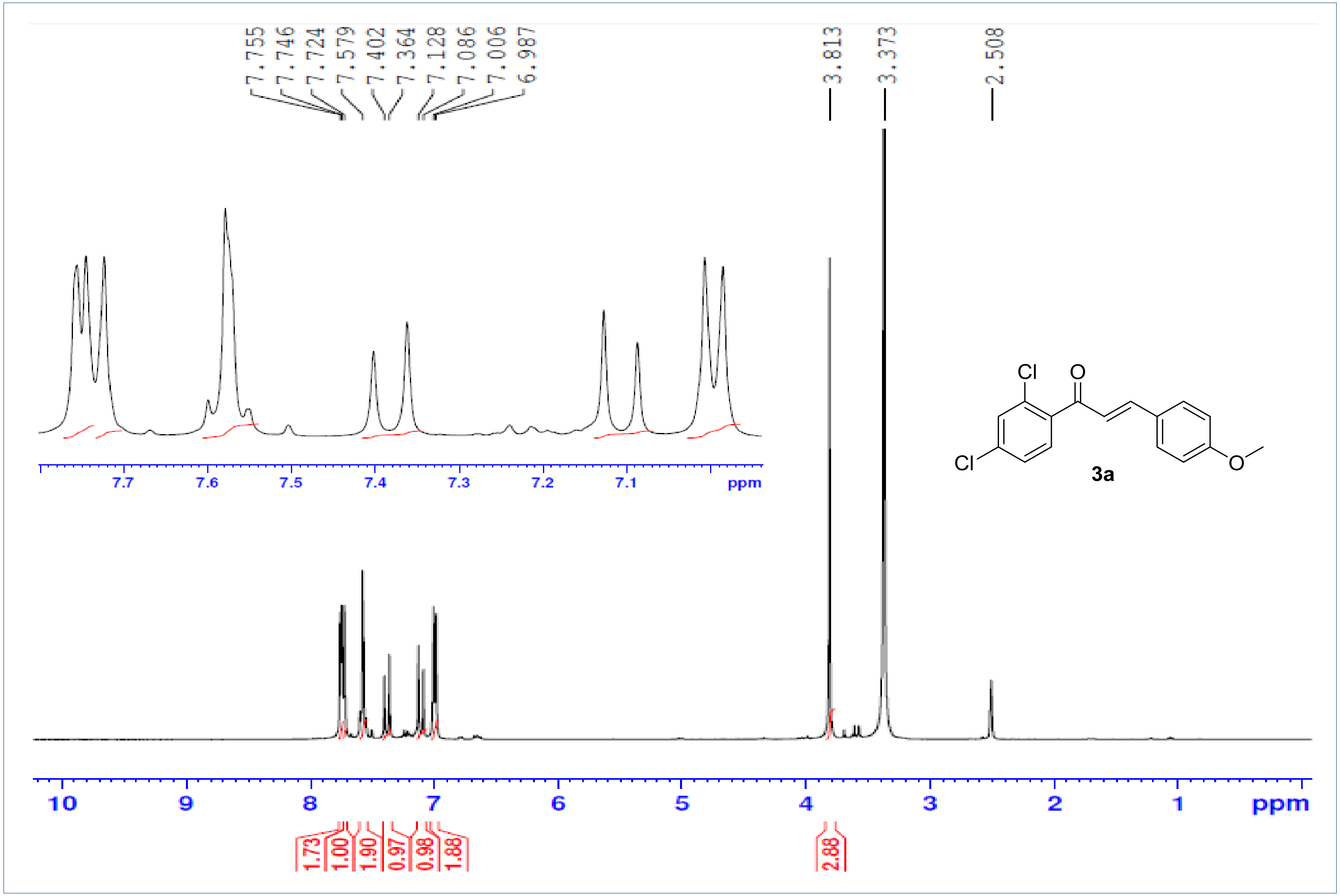

Appendix (1): ^{1}H NMR spectrum of compound **3a** (DMSO-d_6, 400 MHz).

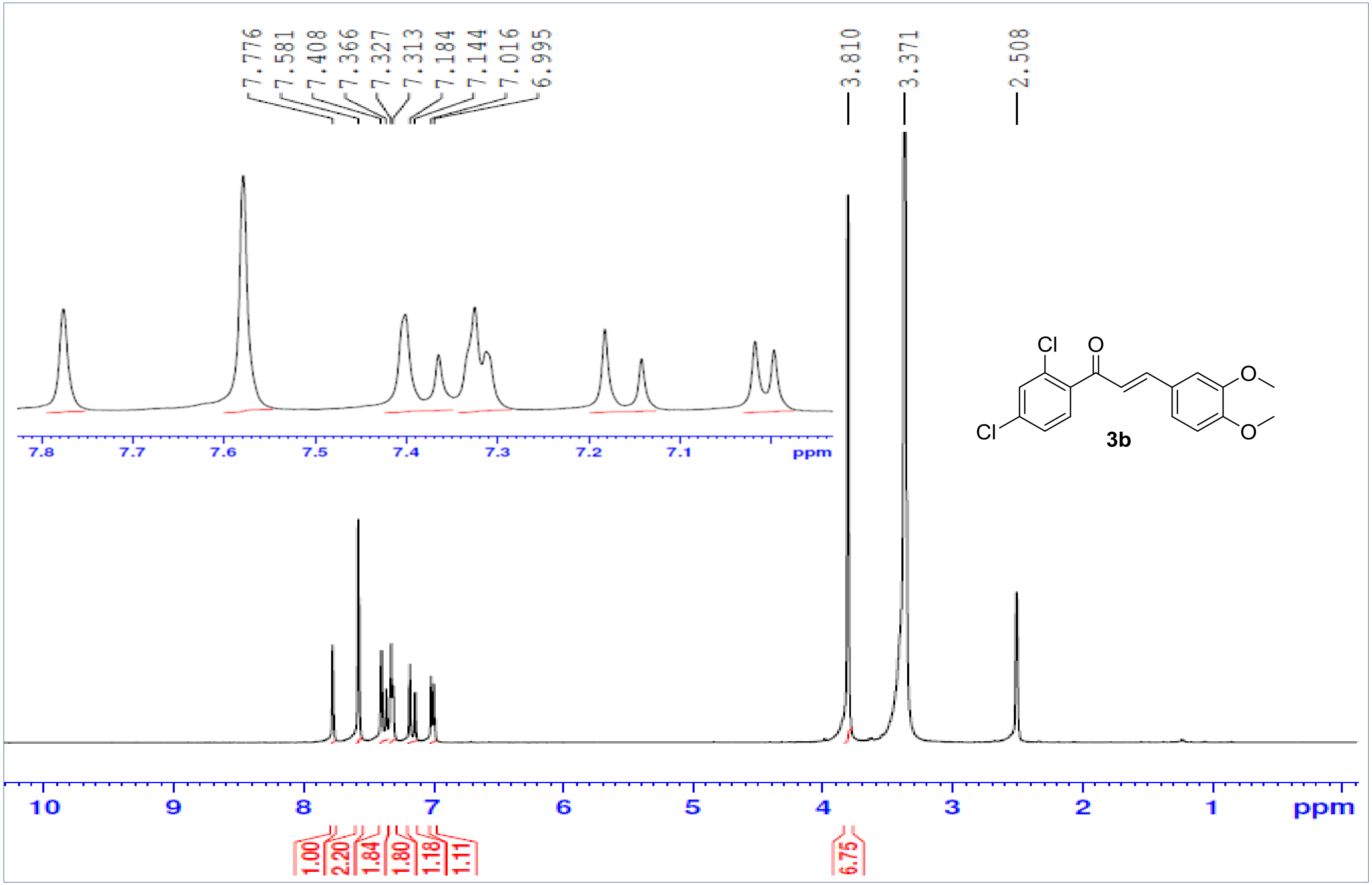

Appendix (2): ^{1}H NMR spectrum of compound **3b** (DMSO-d_6, 400 MHz).

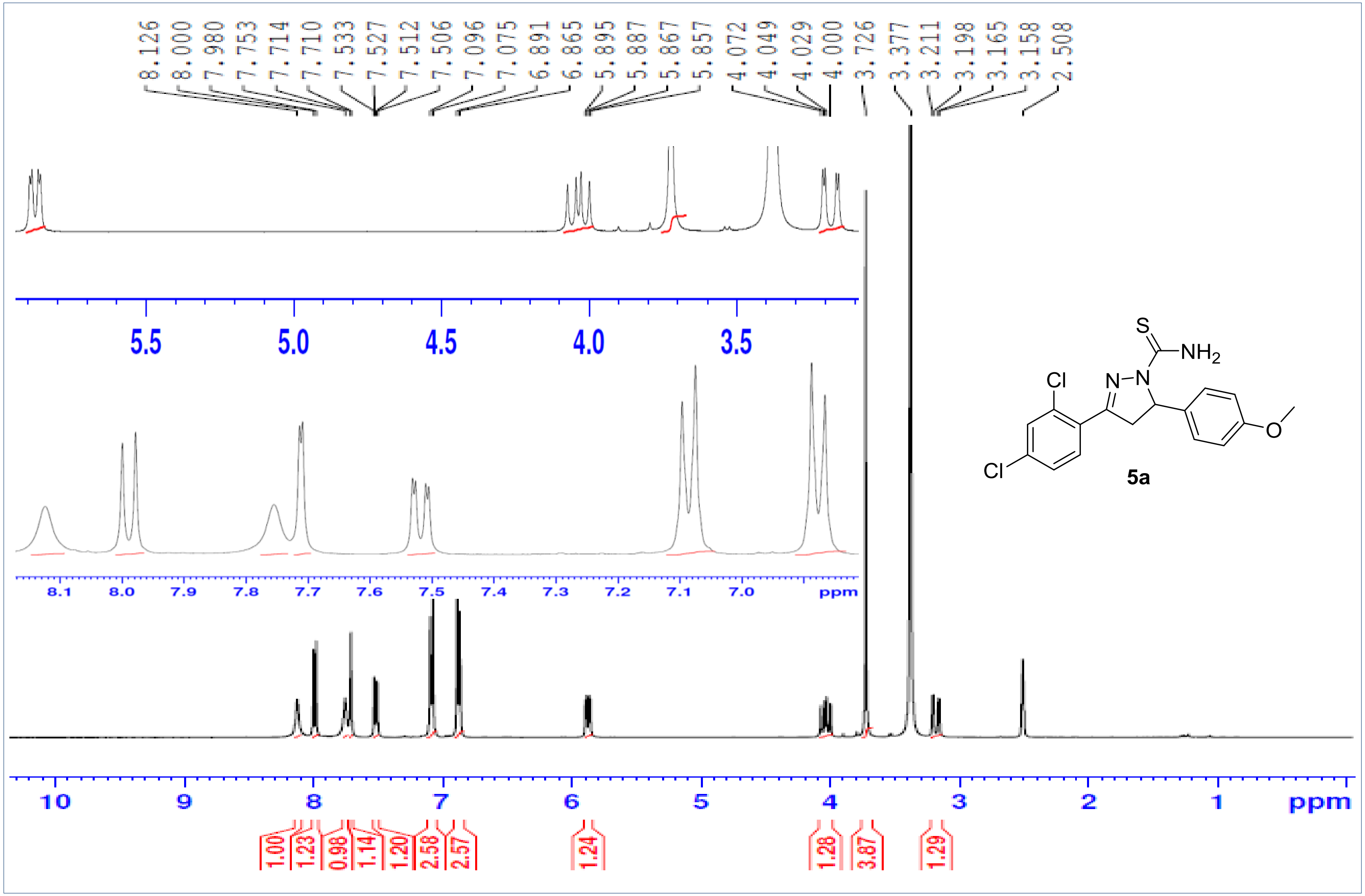

Appendix (3): ^{1}H NMR spectrum of compound **5a** (DMSO-d_6, 400 MHz).

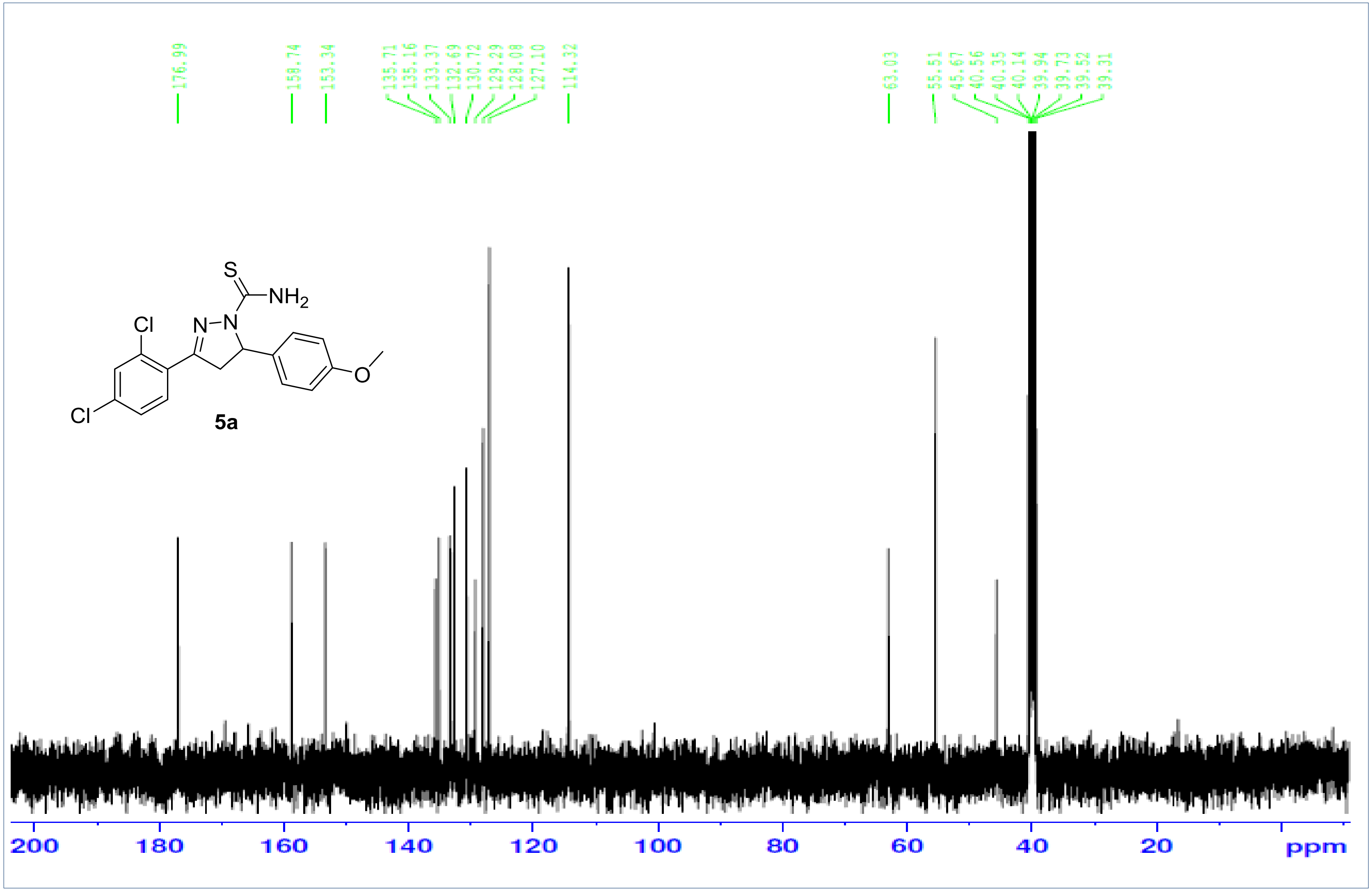

Appendix (4): ^{13}C NMR spectrum of compound **5a** (DMSO-d_6, 100 MHz).

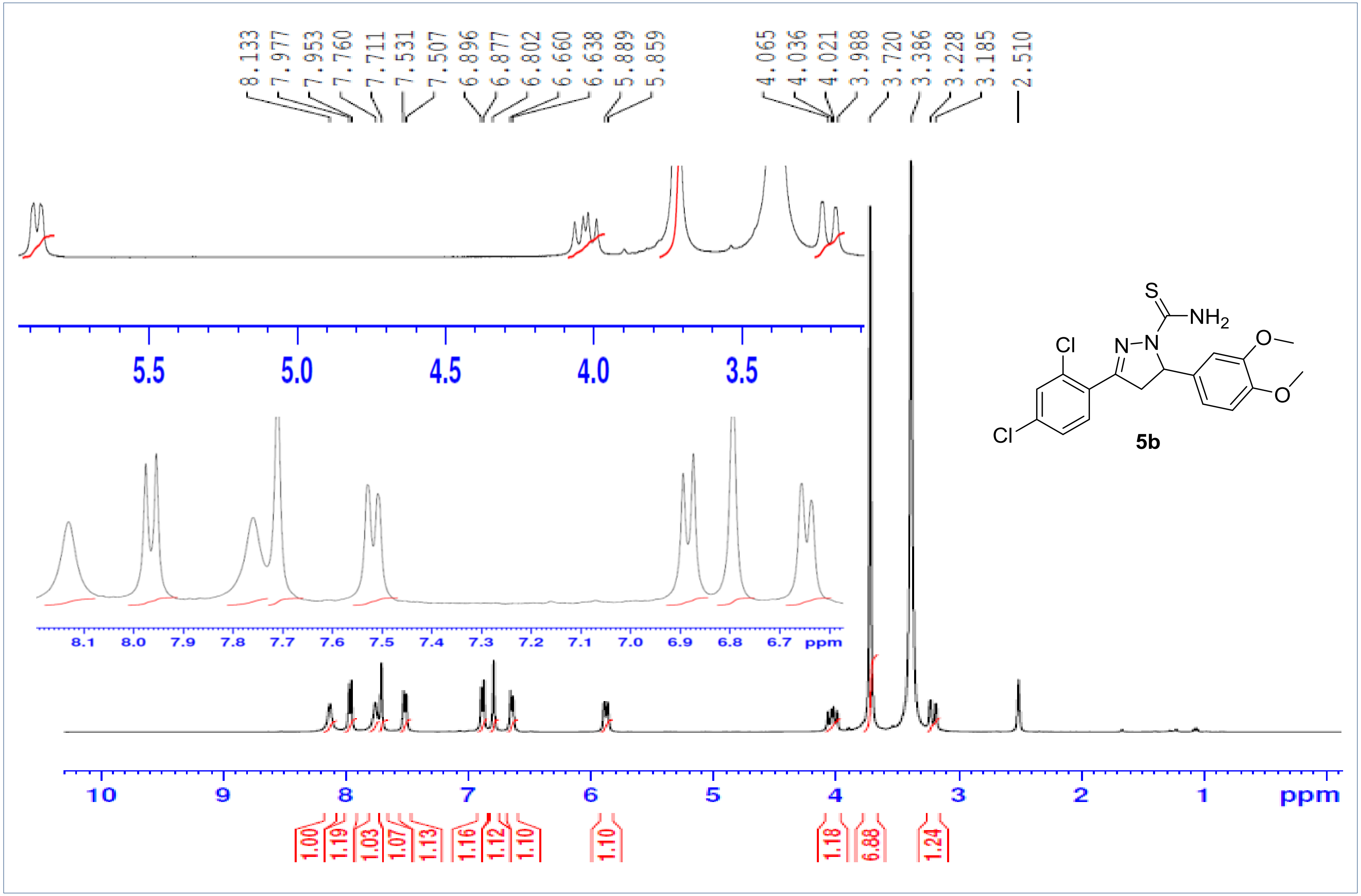

Appendix (5): ^{1}H NMR spectrum of compound **5b** (DMSO-d_6, 400 MHz).

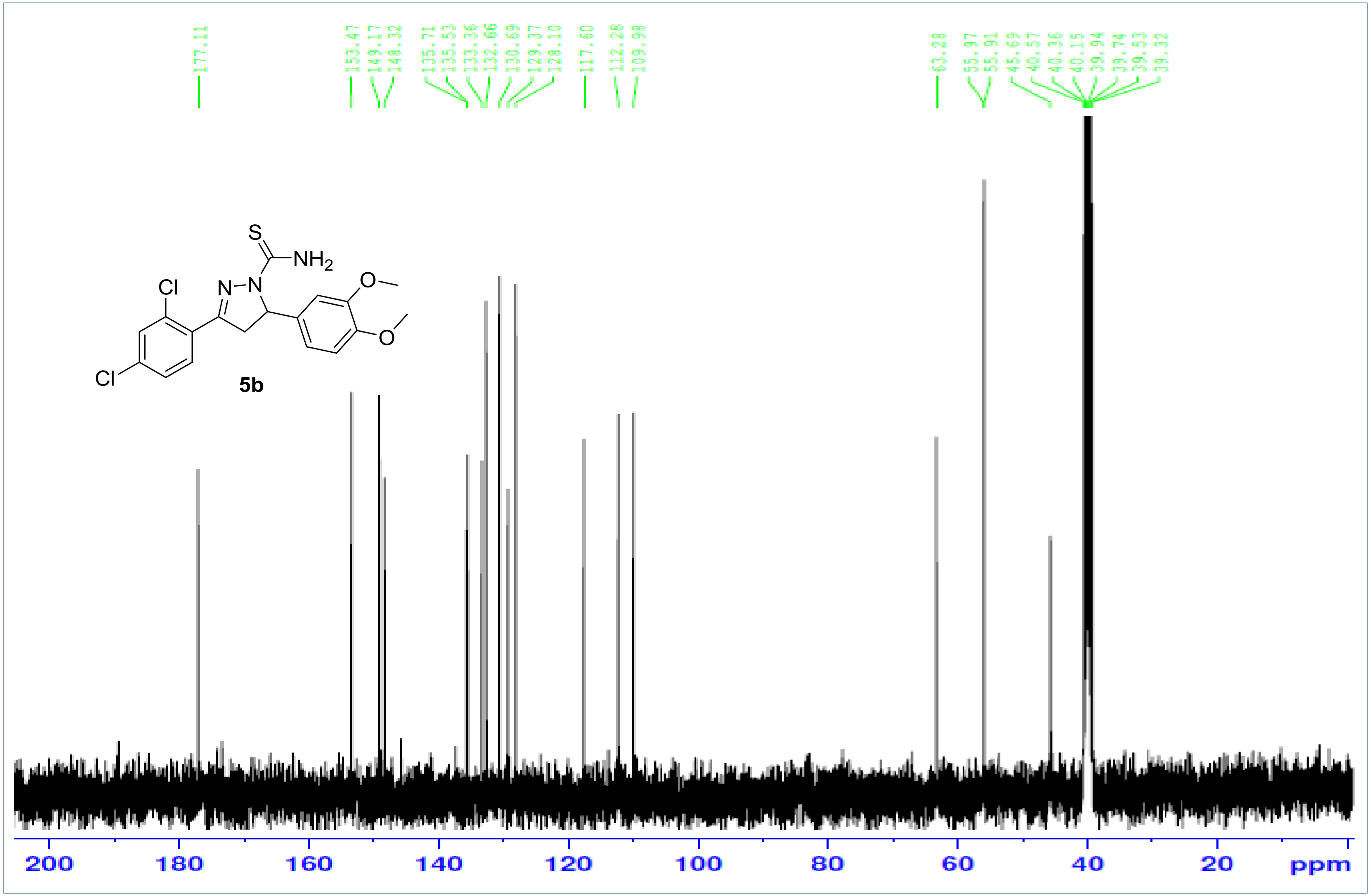

Appendix (6): ^{13}C NMR spectrum of compound **5b** (DMSO-d_6, 100 MHz).

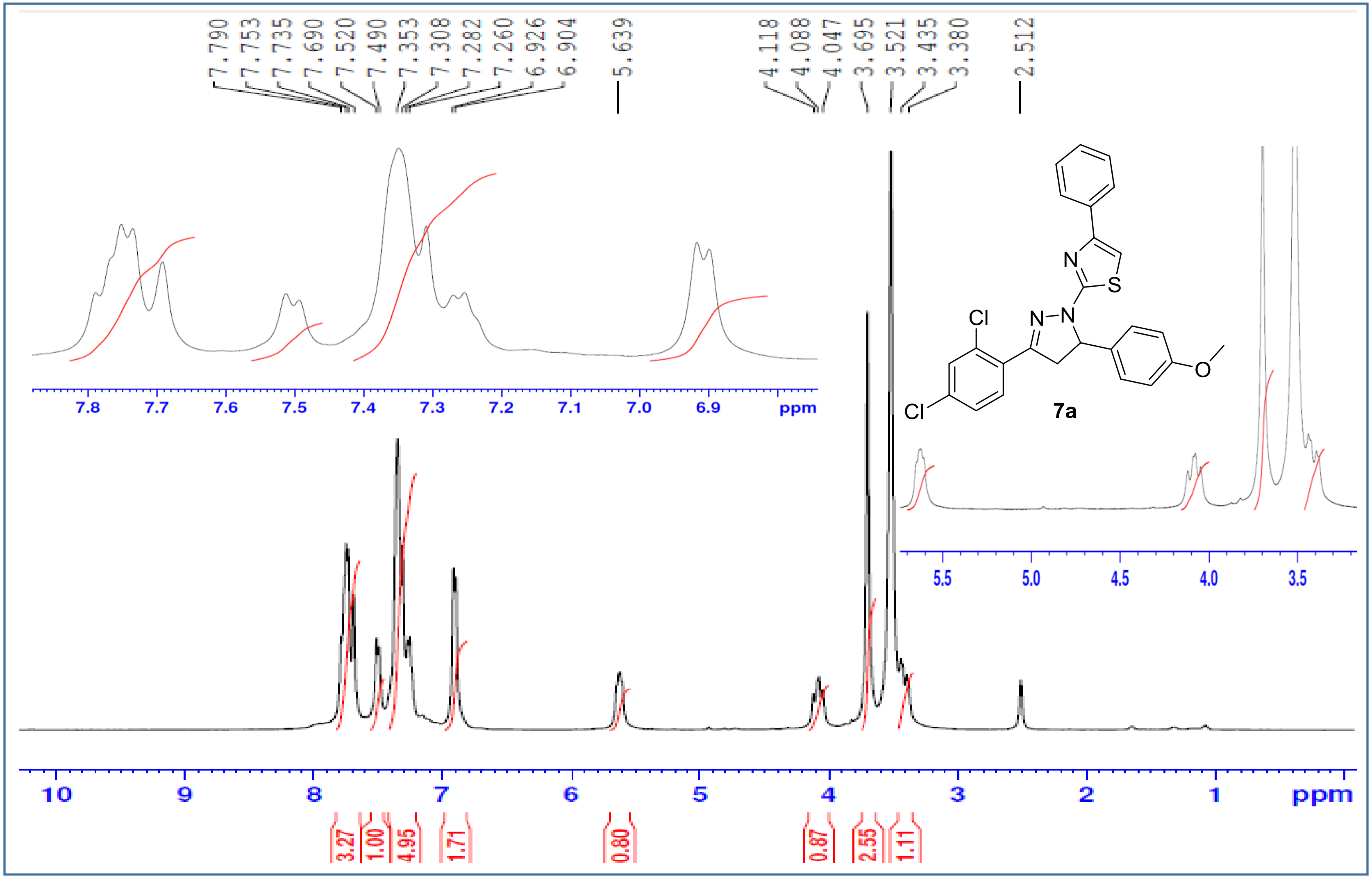

Appendix (7): ^{1}H NMR spectrum of compound **7a** (DMSO-d_6, 400 MHz).

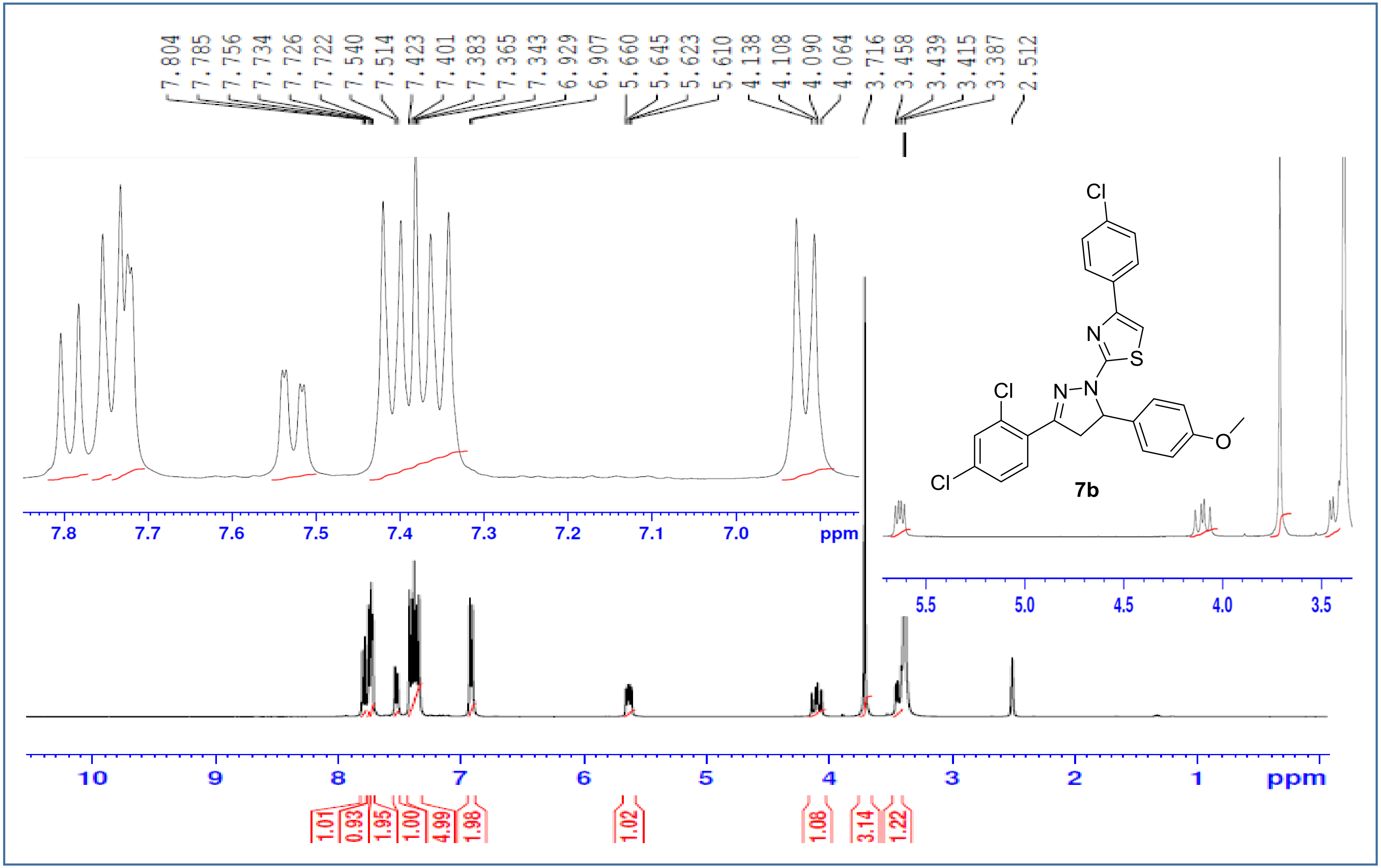

Appendix (8): ^{1}H NMR spectrum of compound **7b** (DMSO-d_6, 400 MHz).

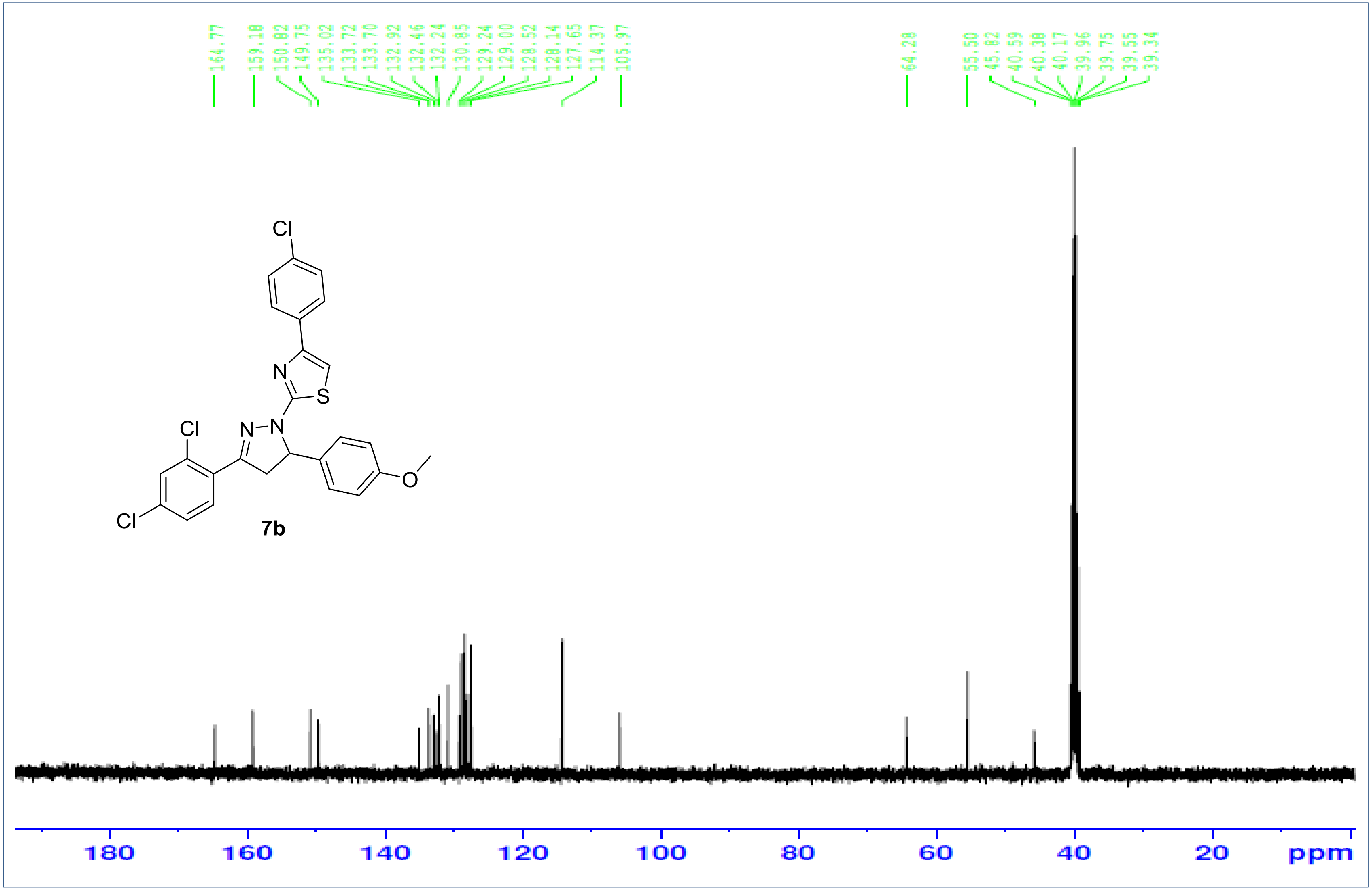

Appendix (9): ^{13}C NMR spectrum of compound **7b** (DMSO-d_6, 100 MHz).

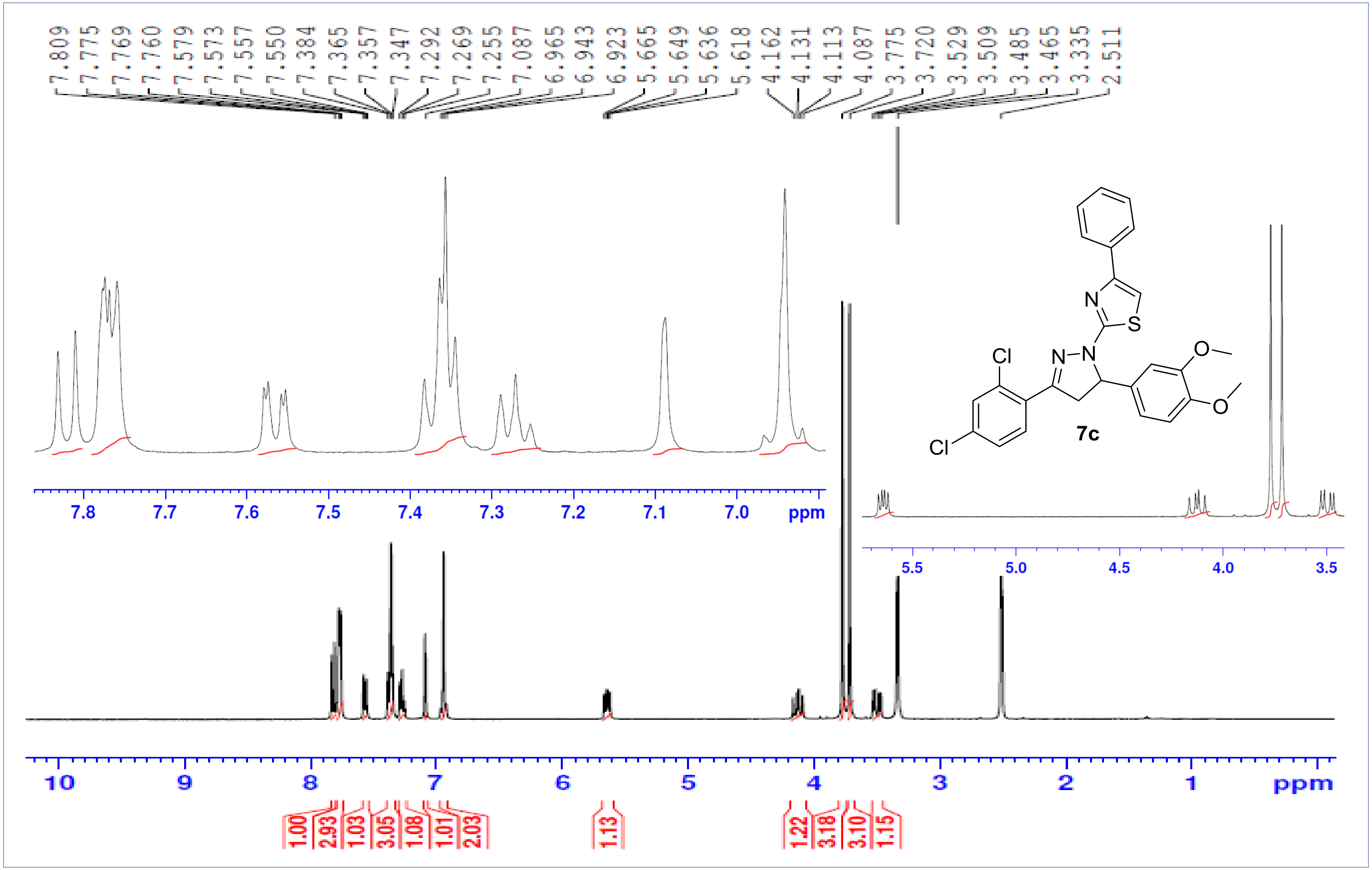

Appendix (10): ^{1}H NMR spectrum of compound **7c** (DMSO-d_6, 400 MHz).

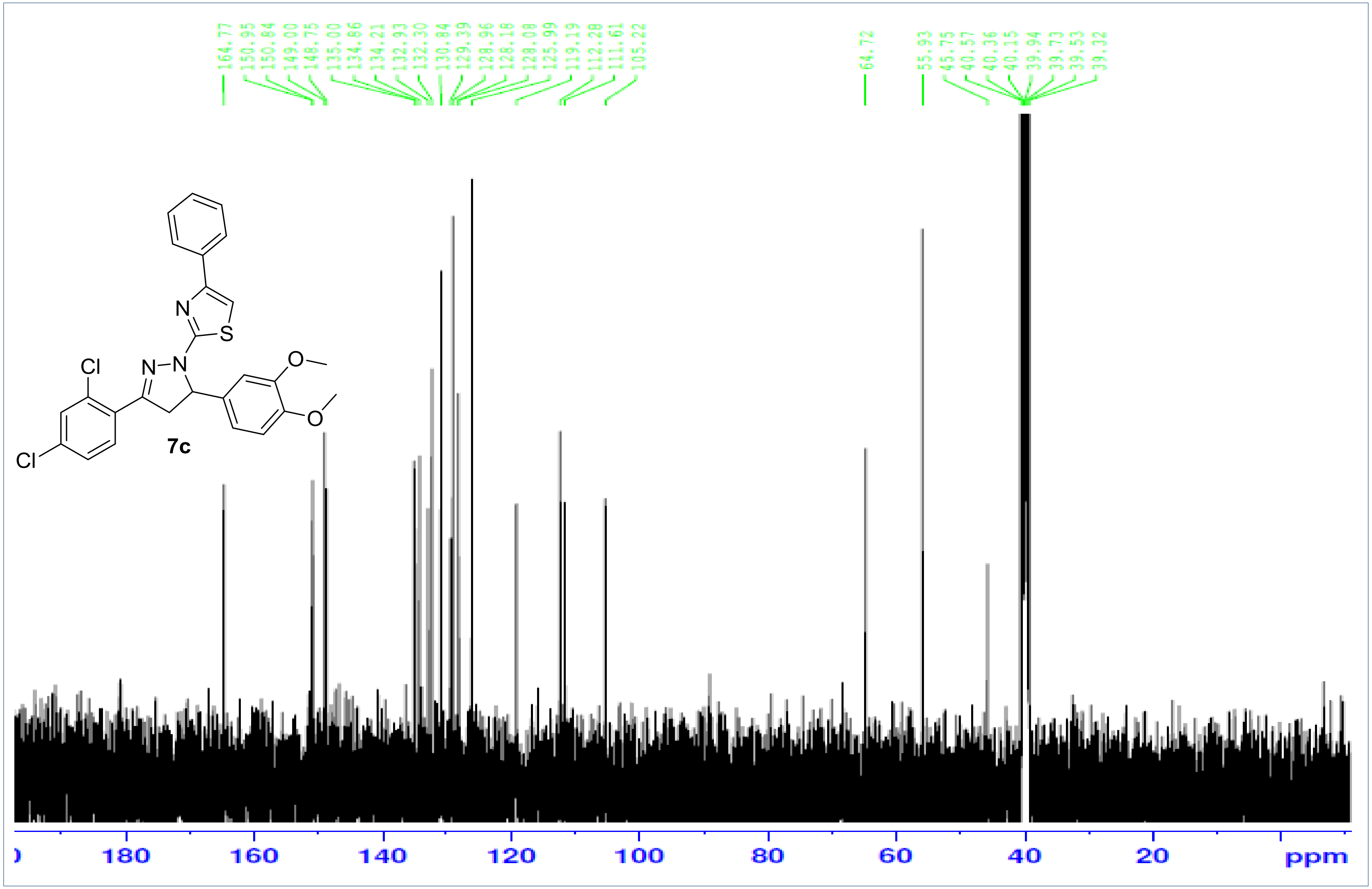

Appendix (11): ^{13}C NMR spectrum of compound **7c** (DMSO-d_6, 100 MHz).

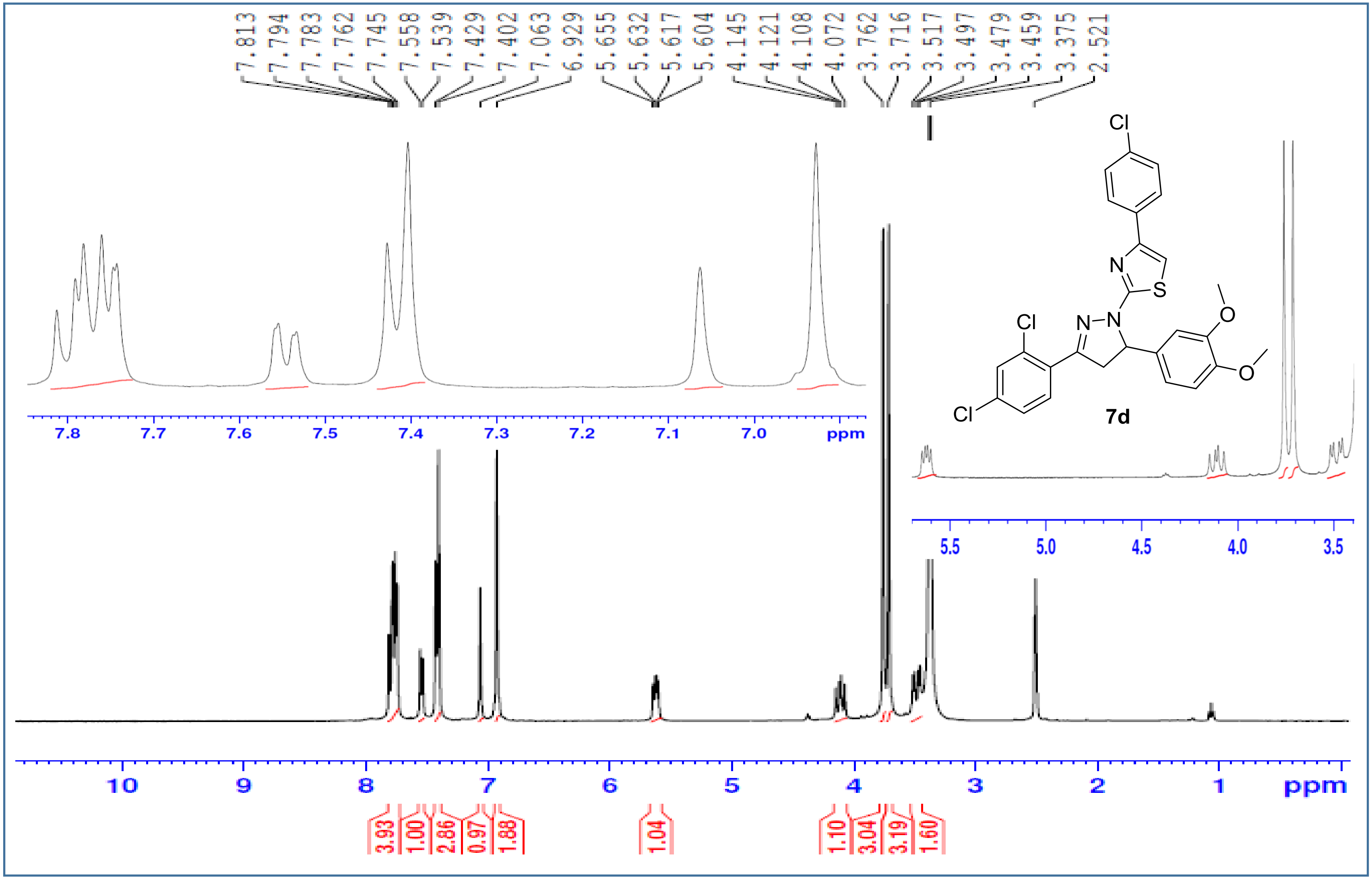

Appendix (12): ^{1}H NMR spectrum of compound **7d** (DMSO-d_6, 400 MHz).

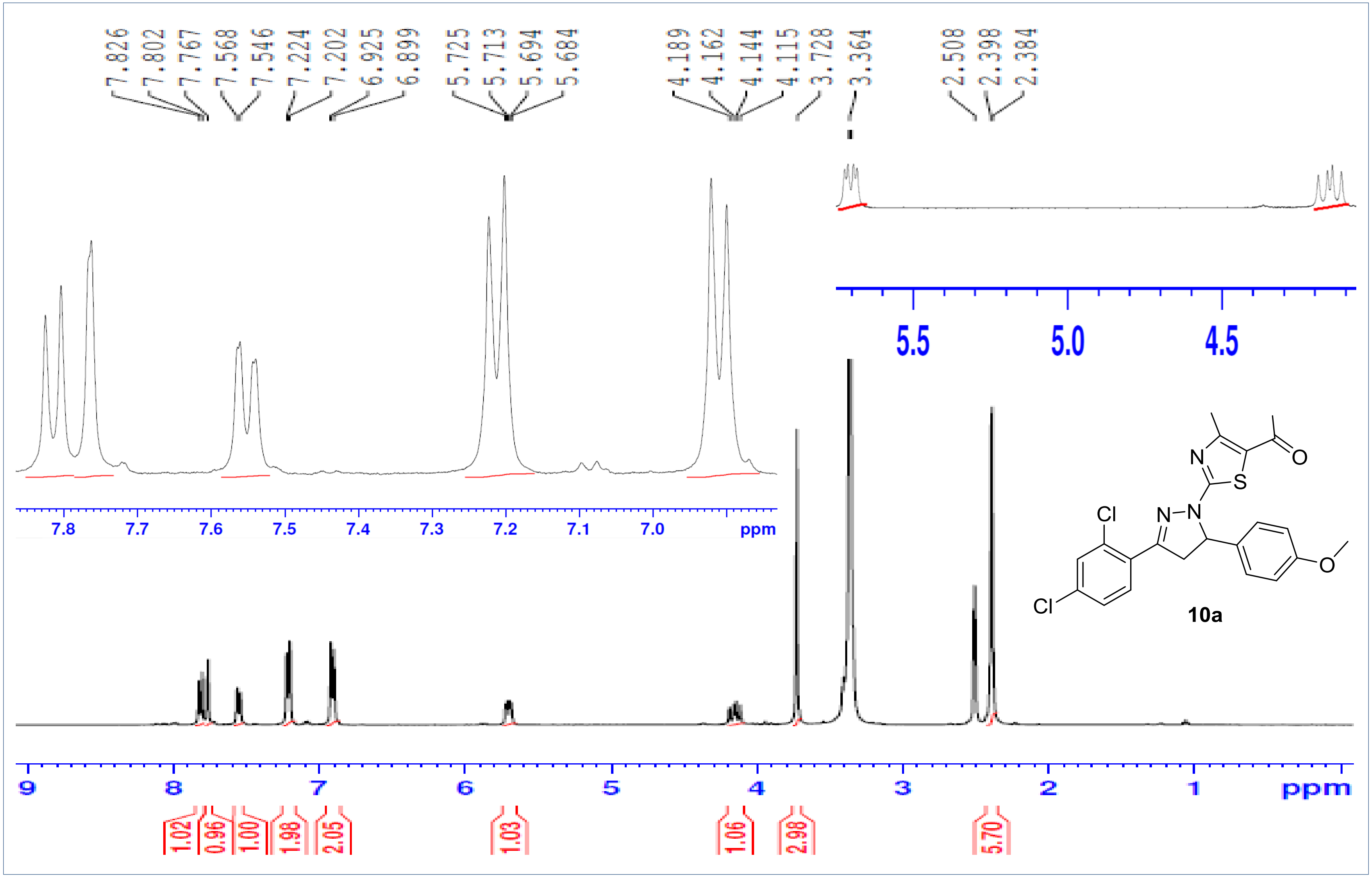

Appendix (13): ^{1}H NMR spectrum of compound **10a** (DMSO-d_6, 400 MHz).

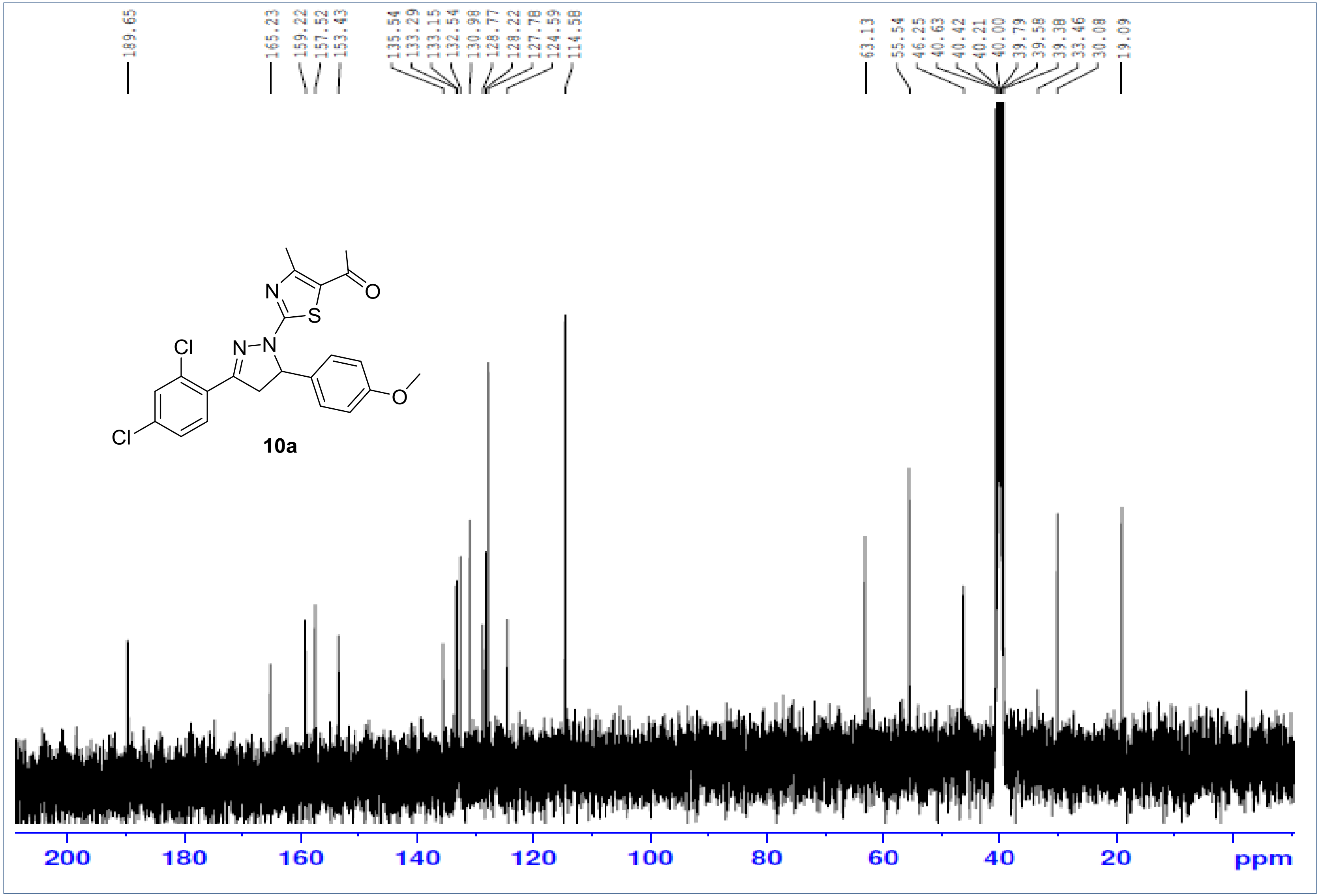

Appendix (14): ^{13}C NMR spectrum of compound **10a** (DMSO-d_6, 100 MHz).

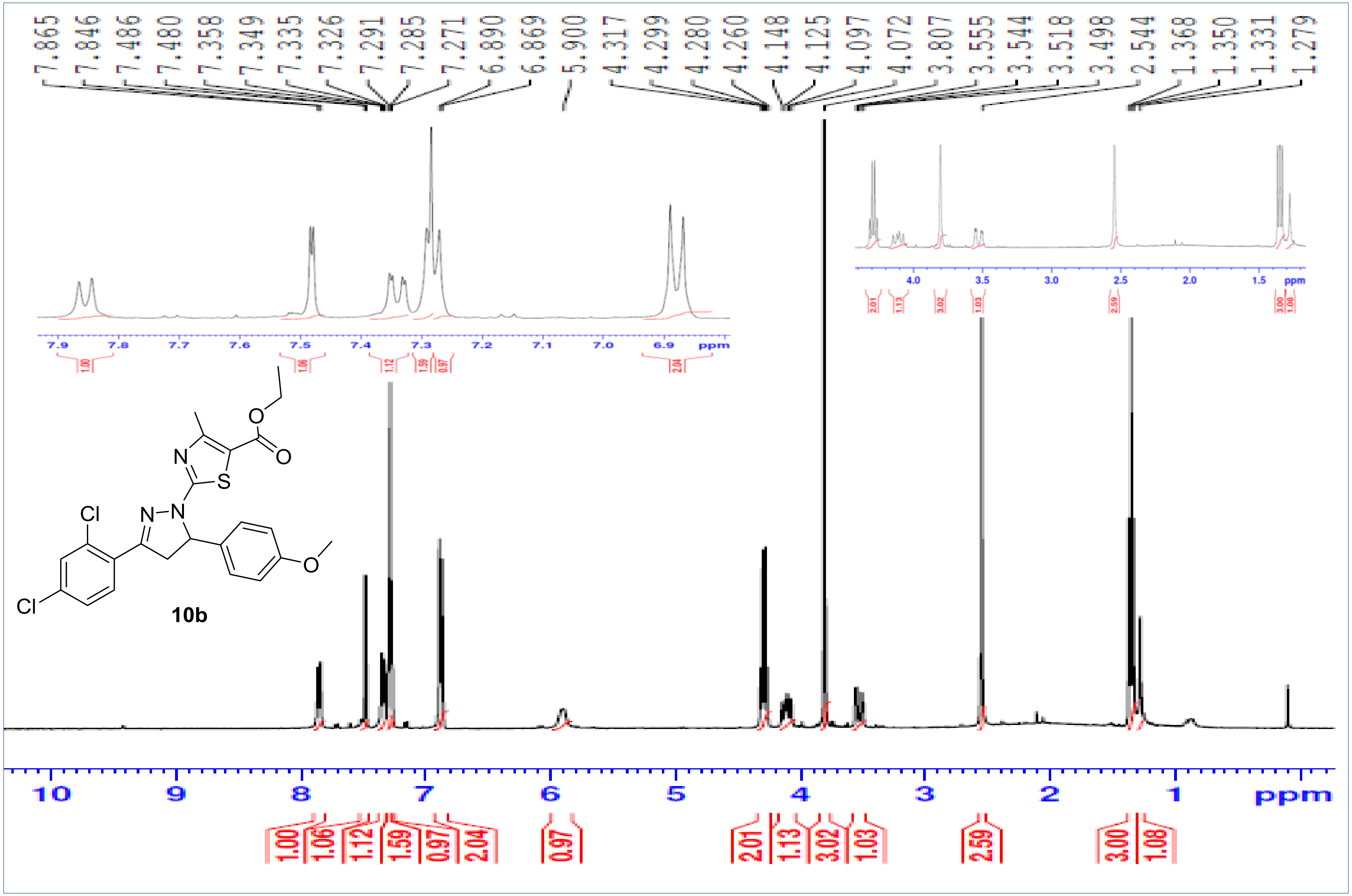

Appendix (15): ^{1}H NMR spectrum of compound **10b** (C*D*Cl_3, 400 MHz).

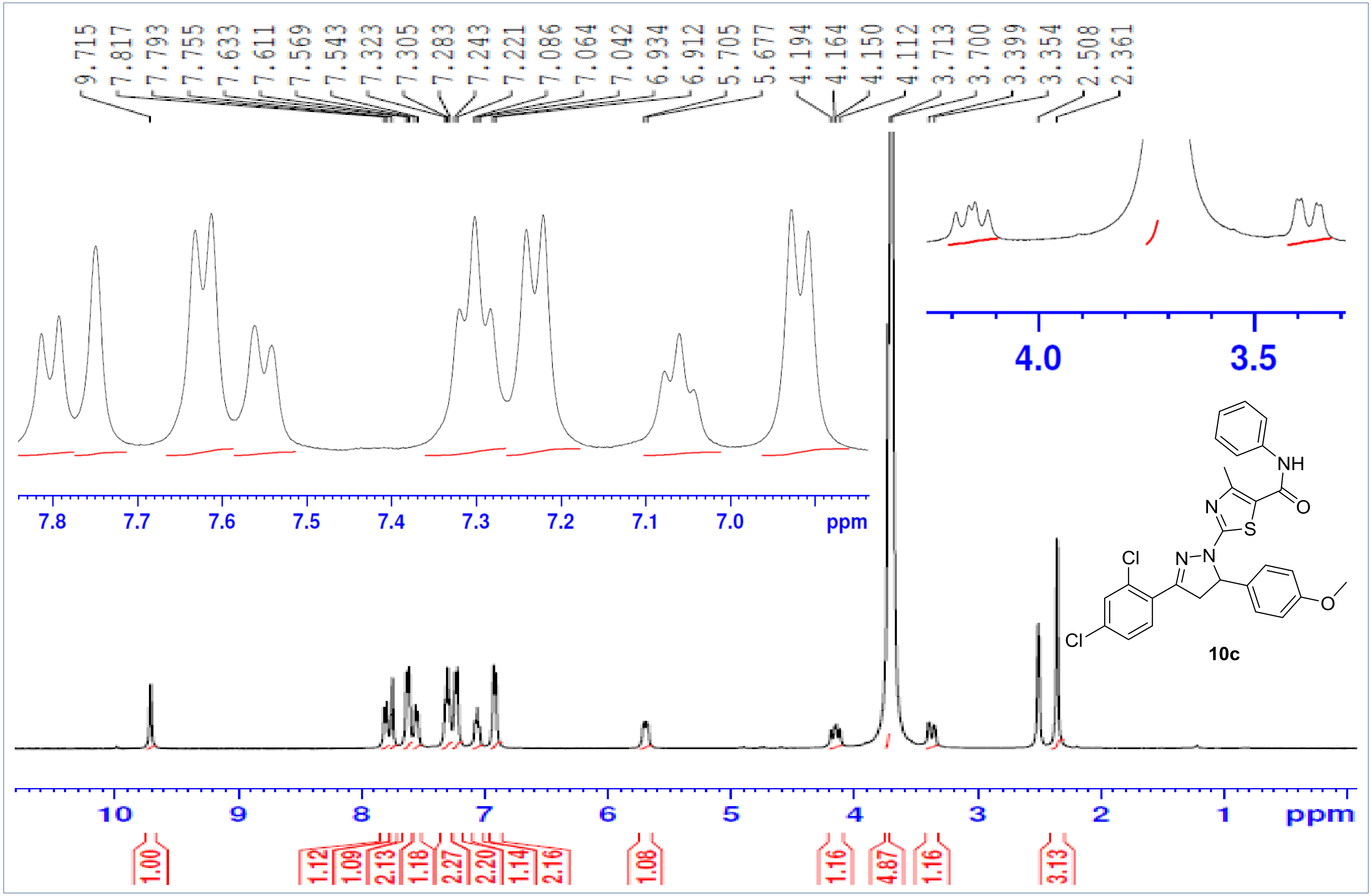

Appendix (16): ^{1}H NMR spectrum of compound **10c** (DMSO-d_6, 400 MHz).

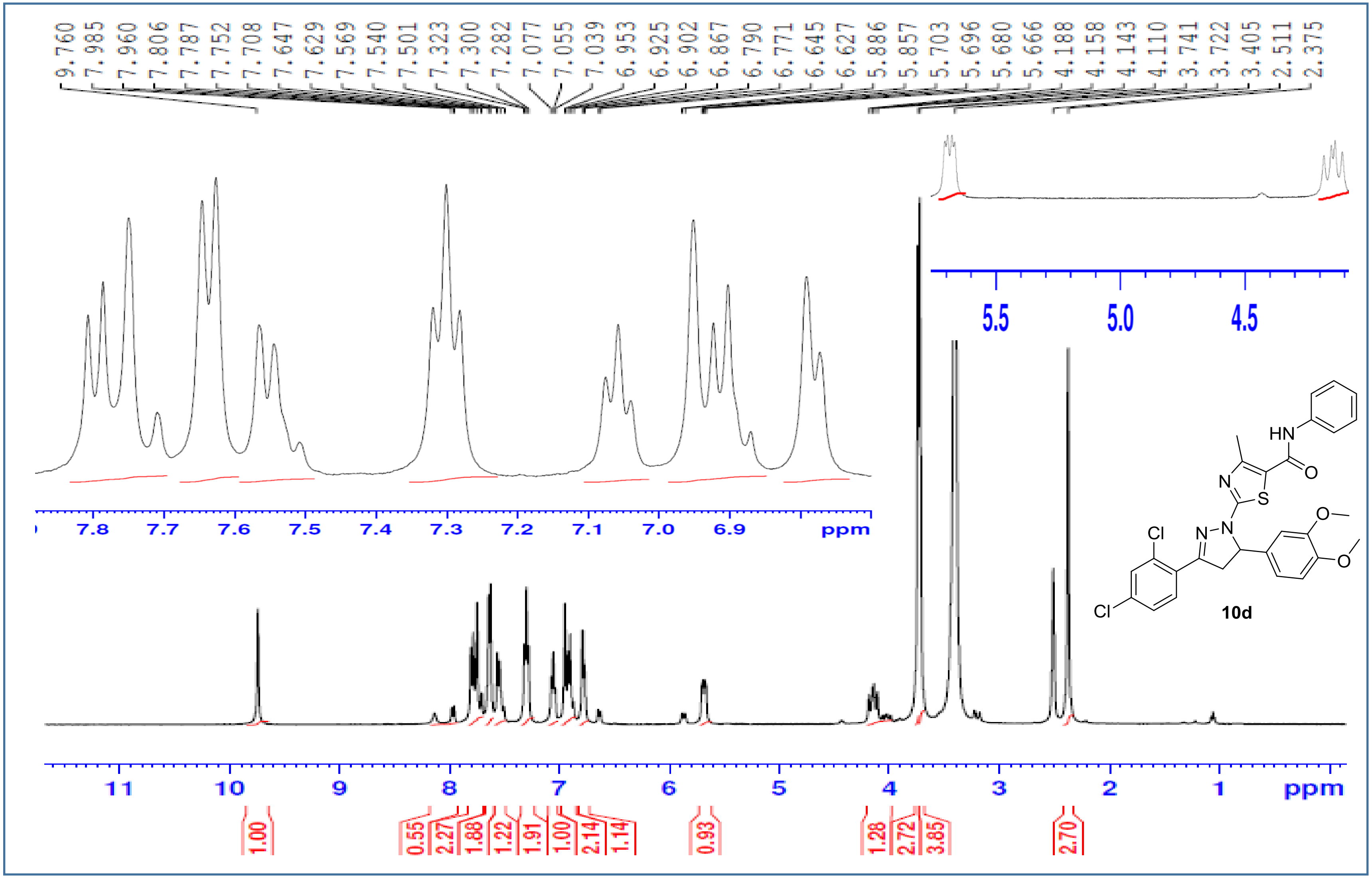

Appendix (17): ^{1}H NMR spectrum of compound **10d** (DMSO-d_6, 400 MHz).

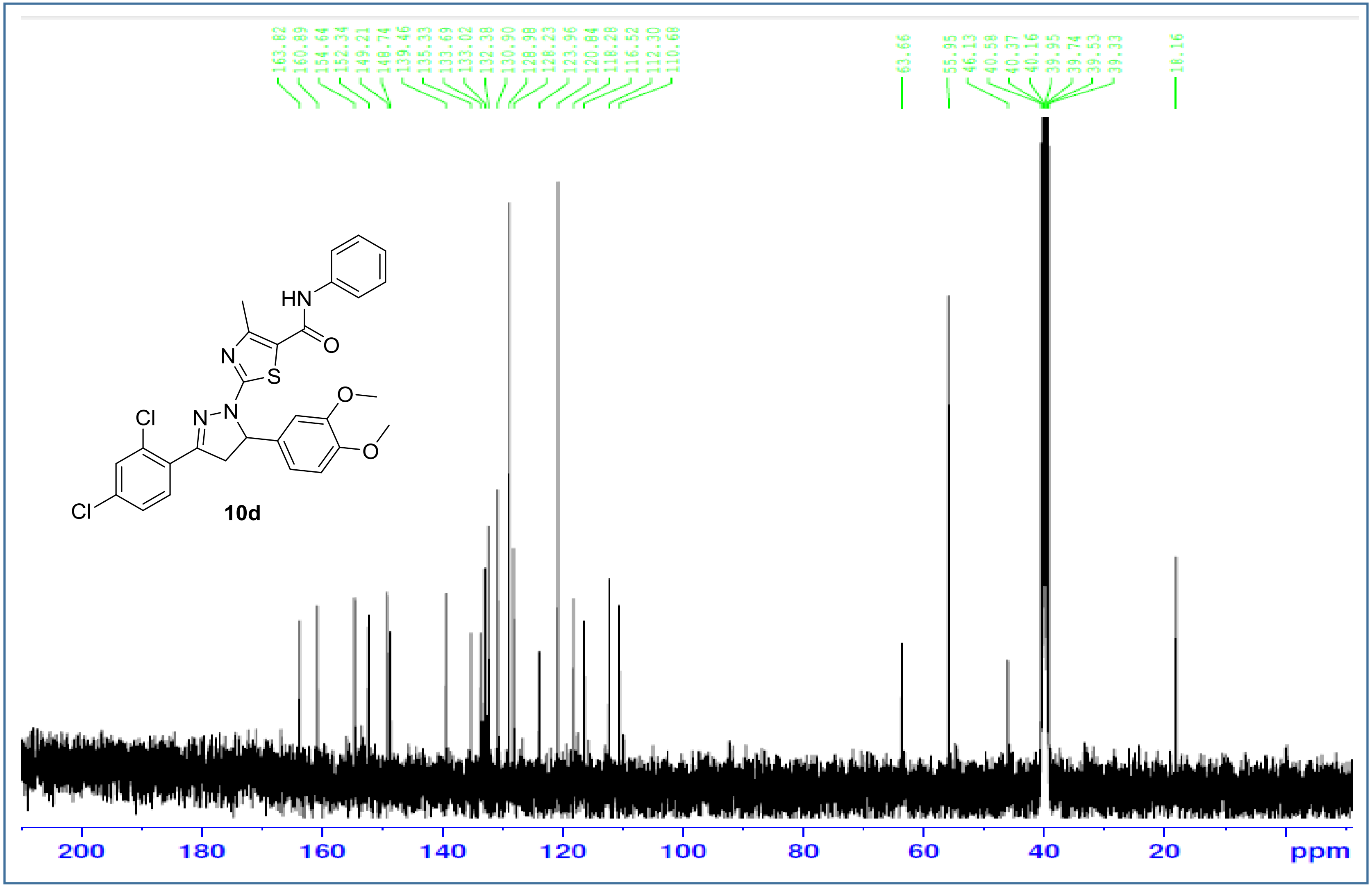

Appendix (18): ^{13}C NMR spectrum of compound **10d** (DMSO-d_6, 100 MHz).

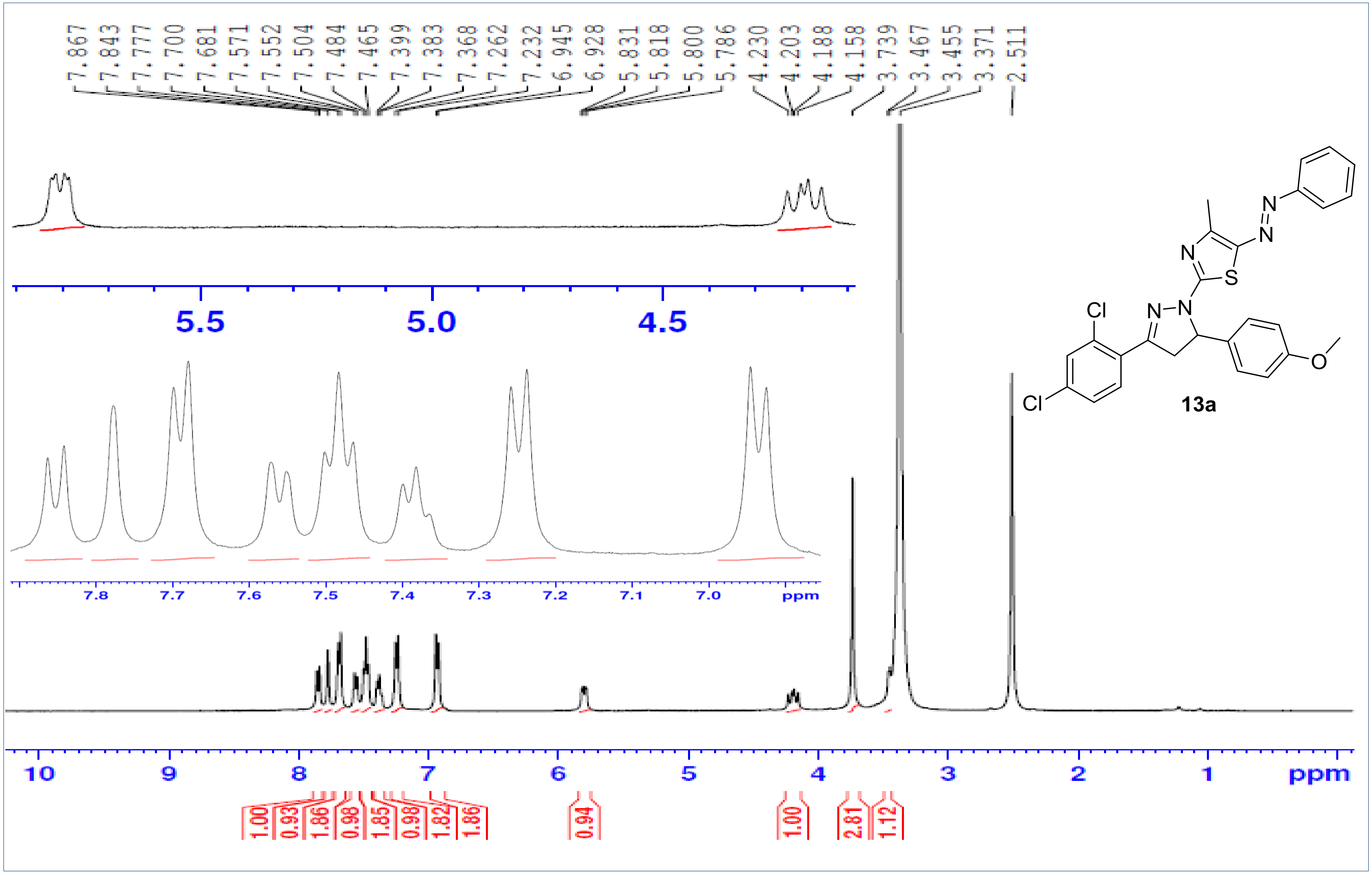

Appendix (19): ^{1}H NMR spectrum of compound **13a** (DMSO-d_6, 400 MHz).

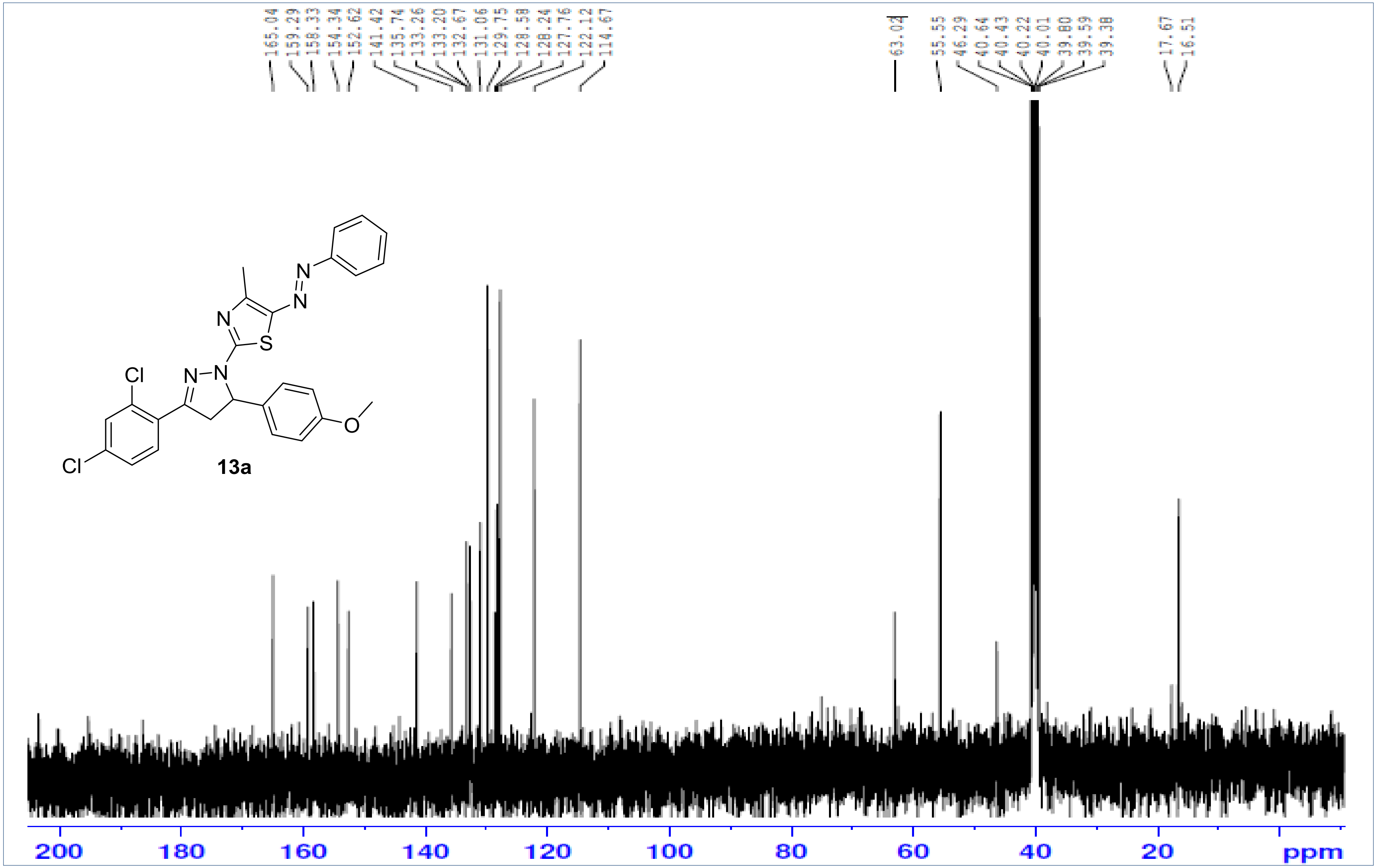

Appendix (20): ^{13}C NMR spectrum of compound **13a** (DMSO-d_6, 100 MHz).

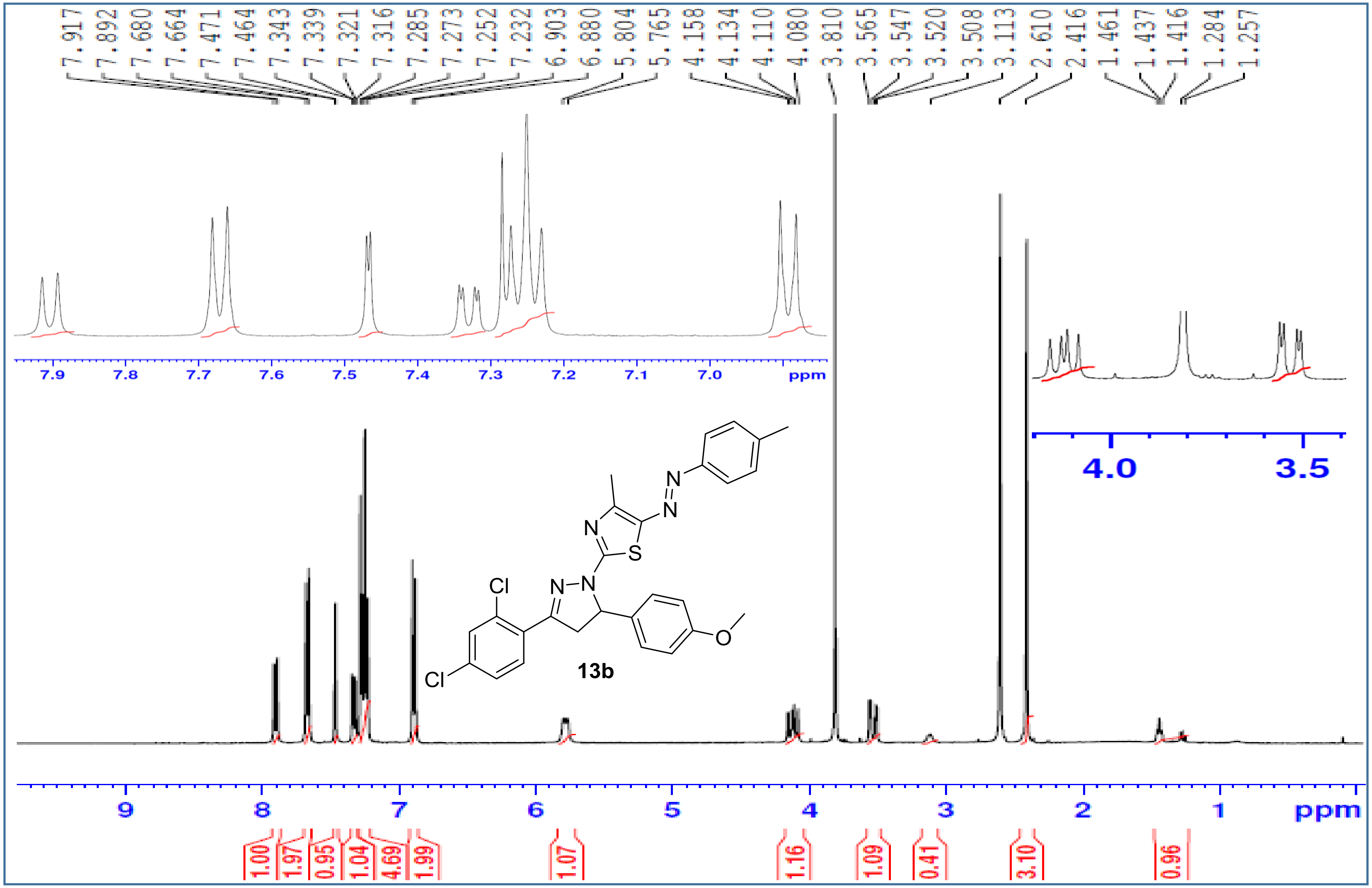

Appendix (21): ¹H NMR spectrum of compound **13b** (C*D*Cl$_3$, 400 MHz).

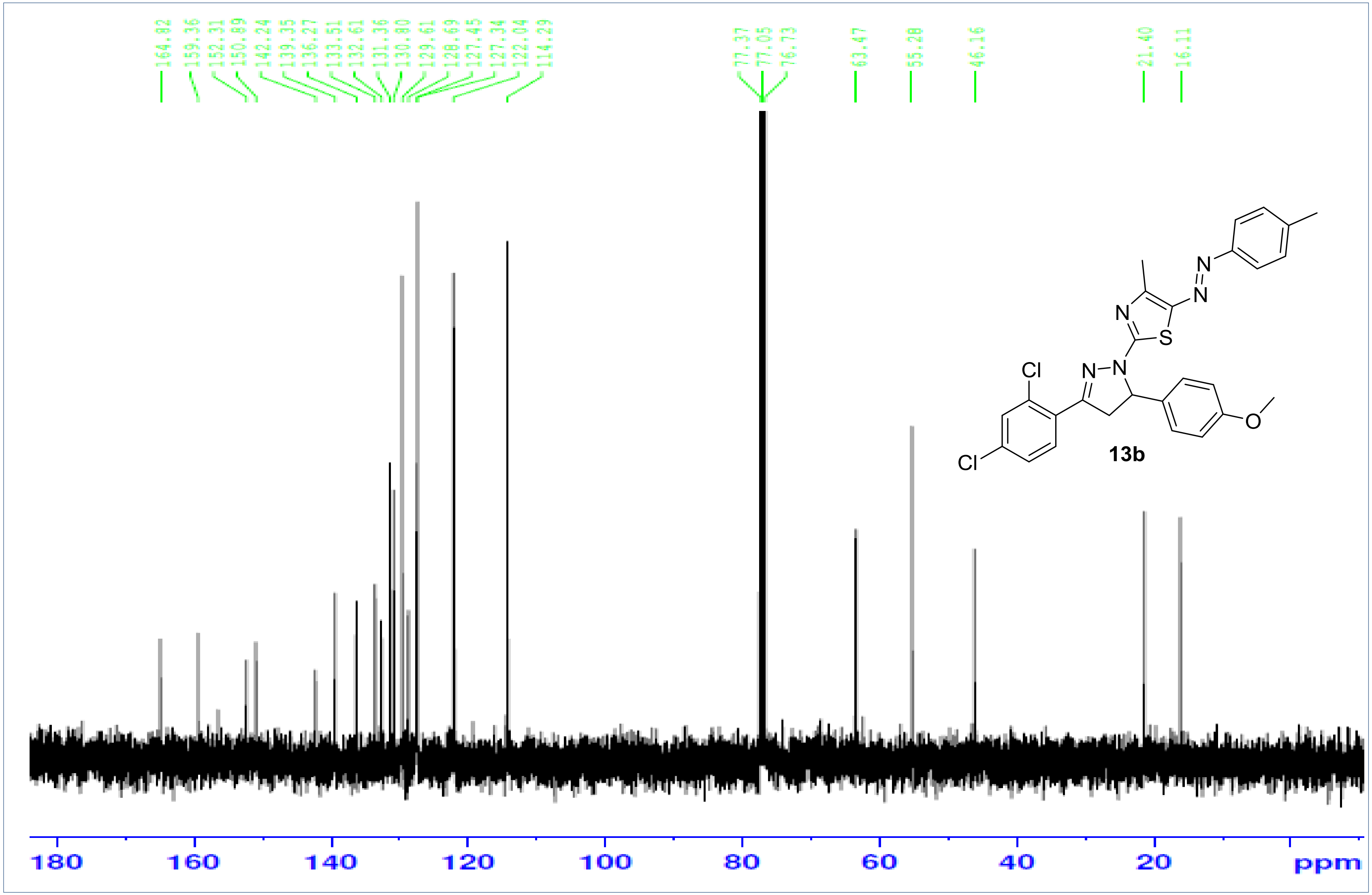

Appendix (22): ^{13}C NMR spectrum of compound **13b** (C*D*Cl_3, 100 MHz).

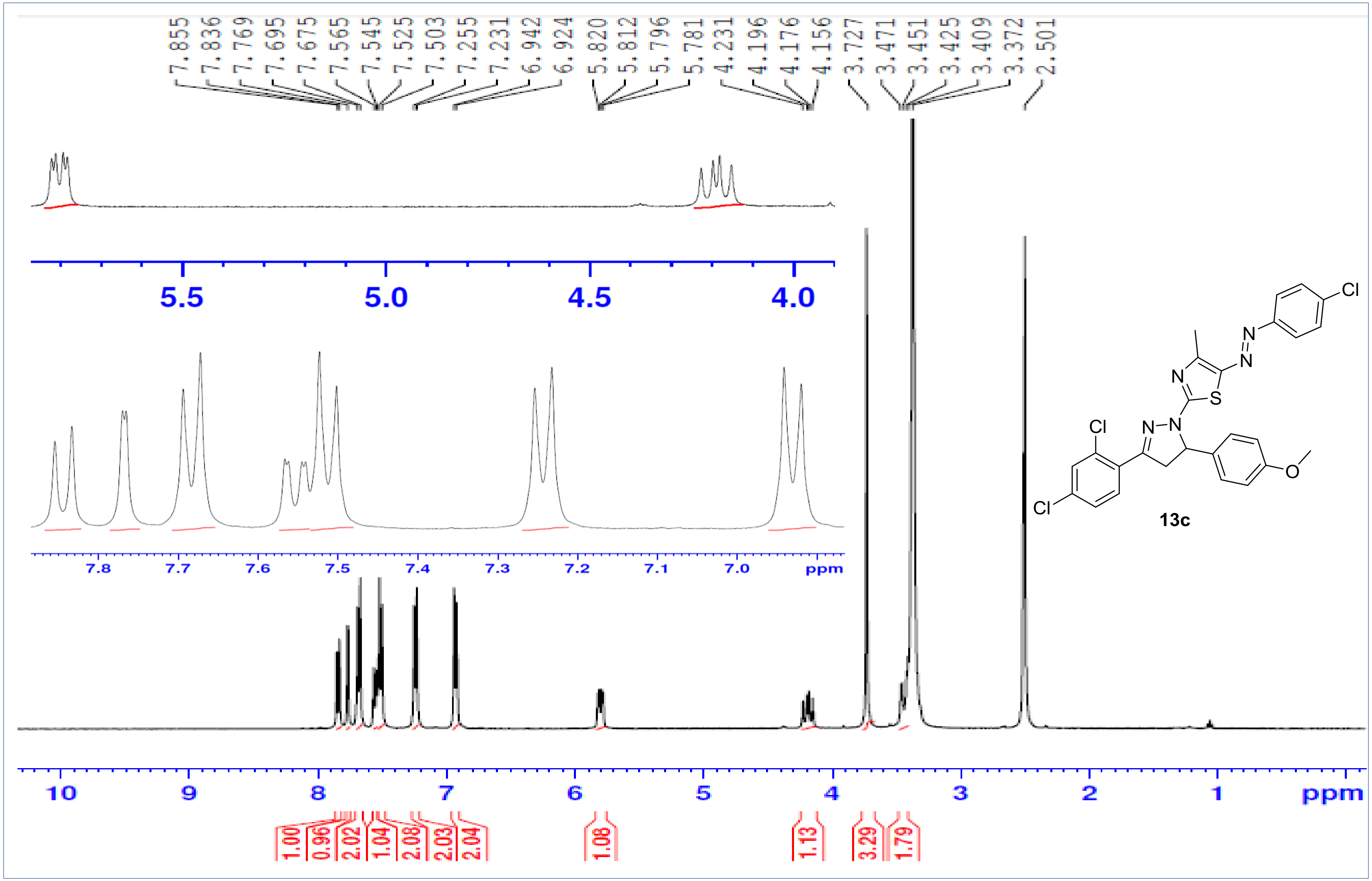

Appendix (23): ¹H NMR spectrum of compound **13c** (DMSO-d_6, 400 MHz).

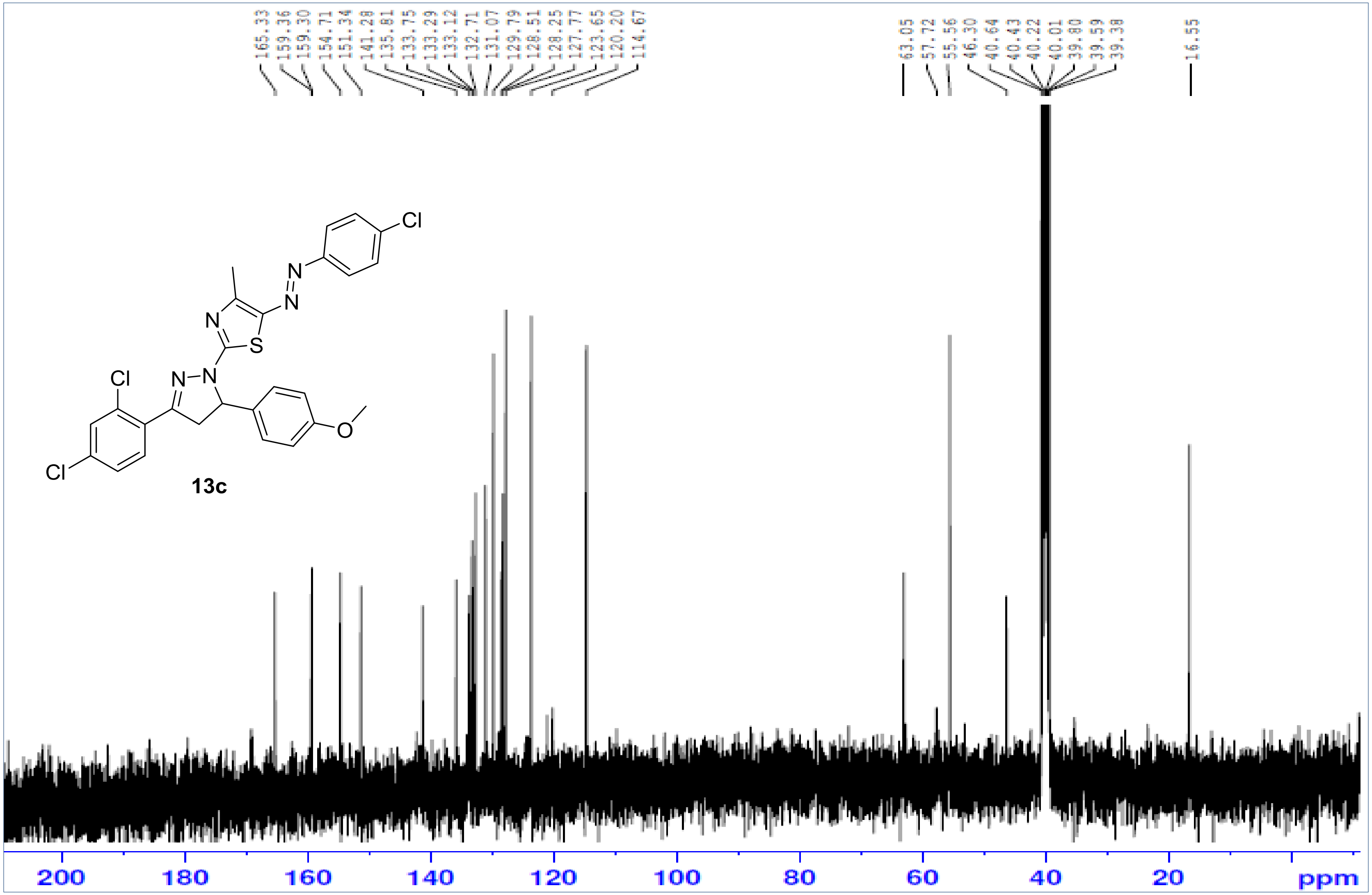

Appendix (24): ^{13}C NMR spectrum of compound **13c** (DMSO-d_6, 100 MHz).

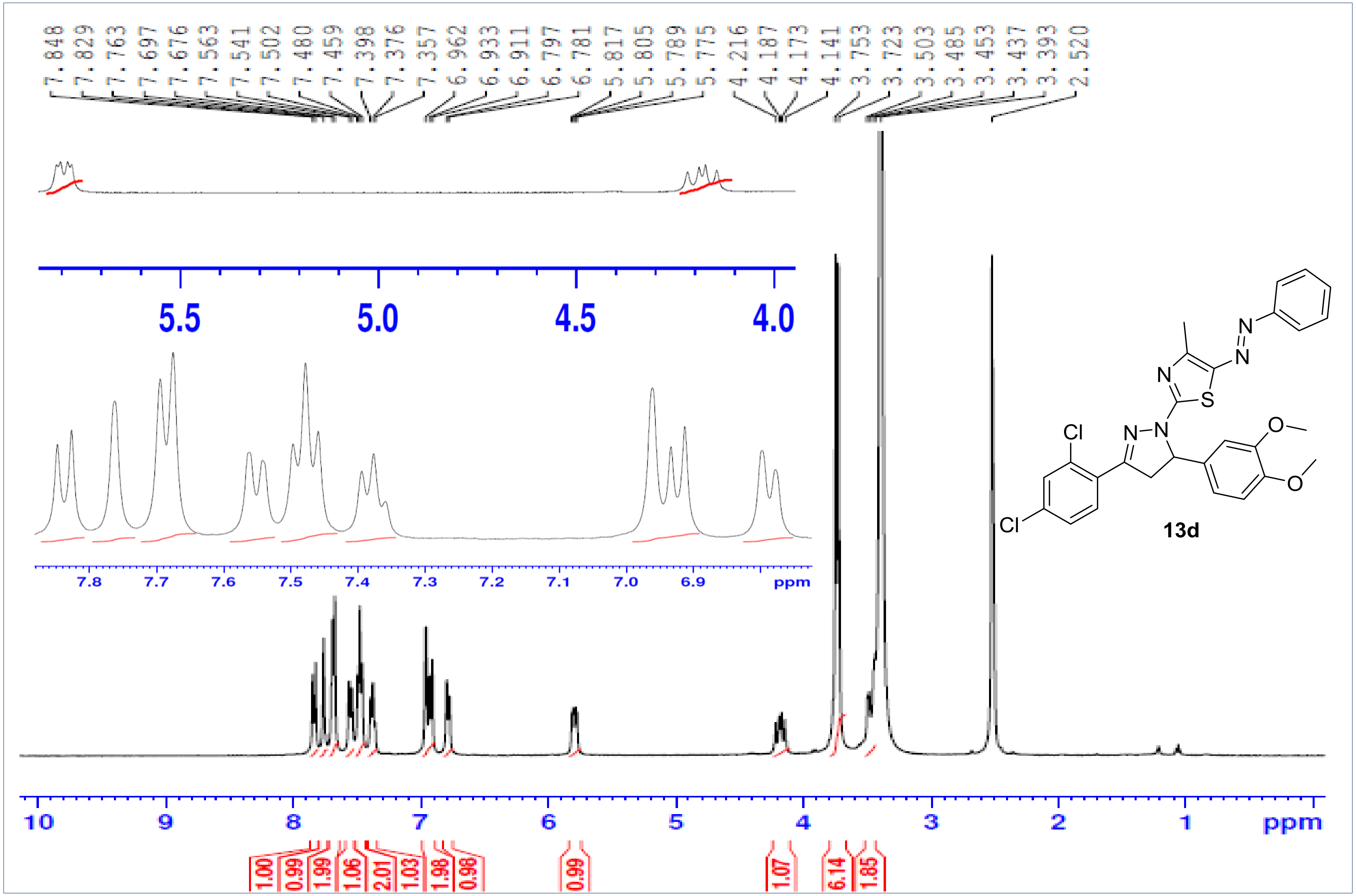

Appendix (25): ^{1}H NMR spectrum of compound **13d** (DMSO-d_6, 400 MHz).

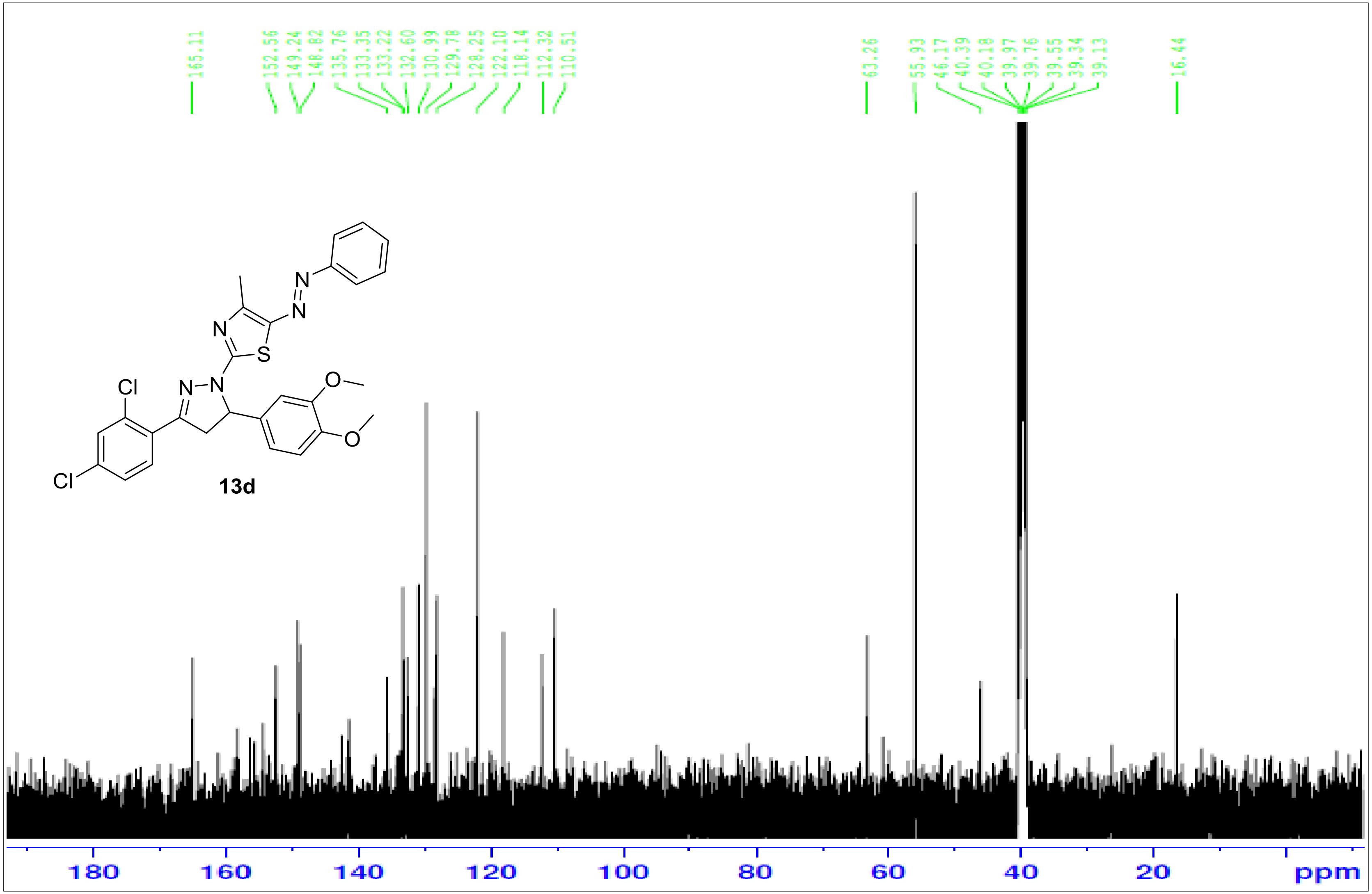

Appendix (26): ^{13}C NMR spectrum of compound **13d** (DMSO-d_6, 100 MHz).

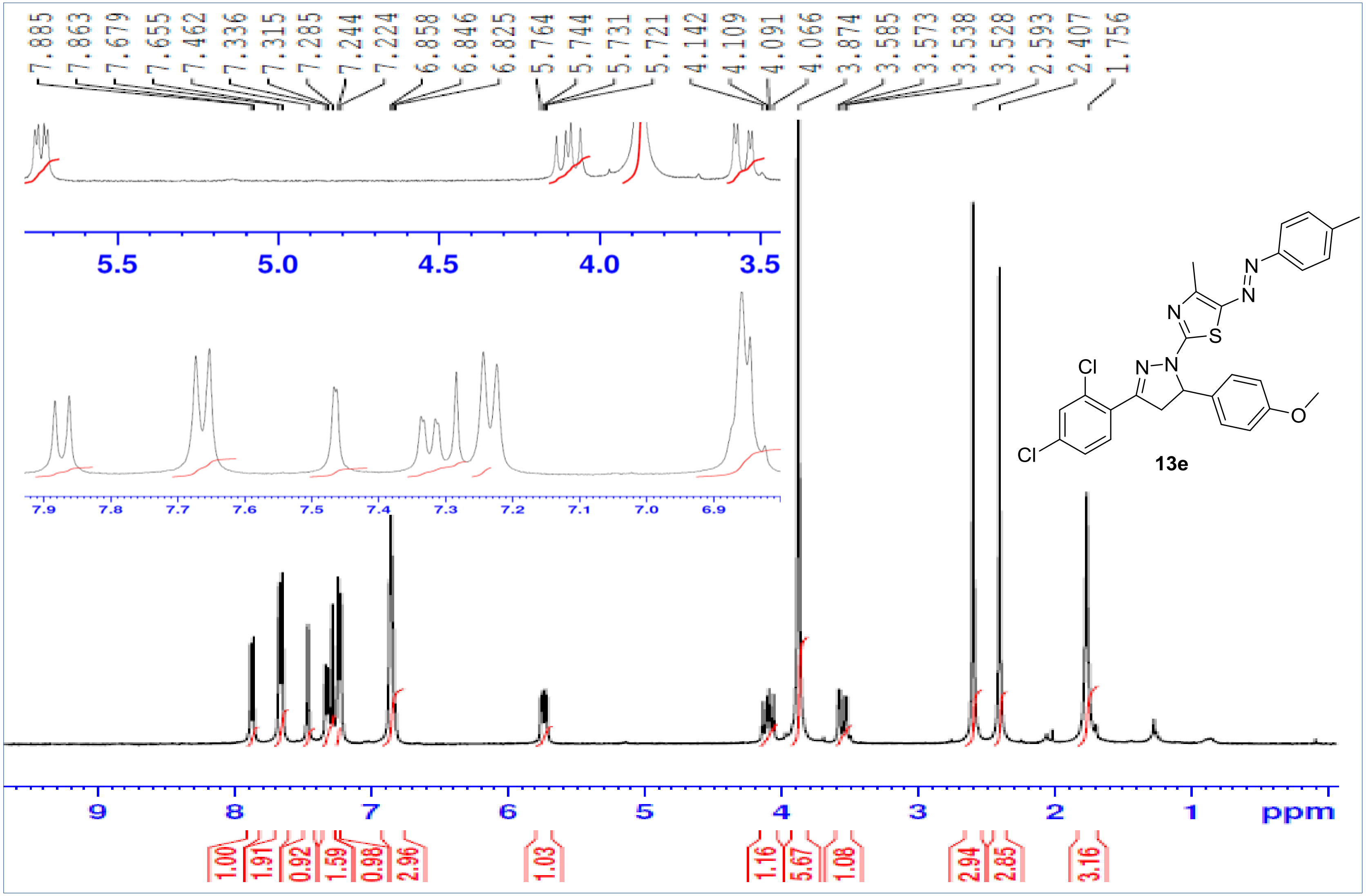

Appendix (27): ^{1}H NMR spectrum of compound **13e** (C*D*Cl$_3$, 400 MHz).

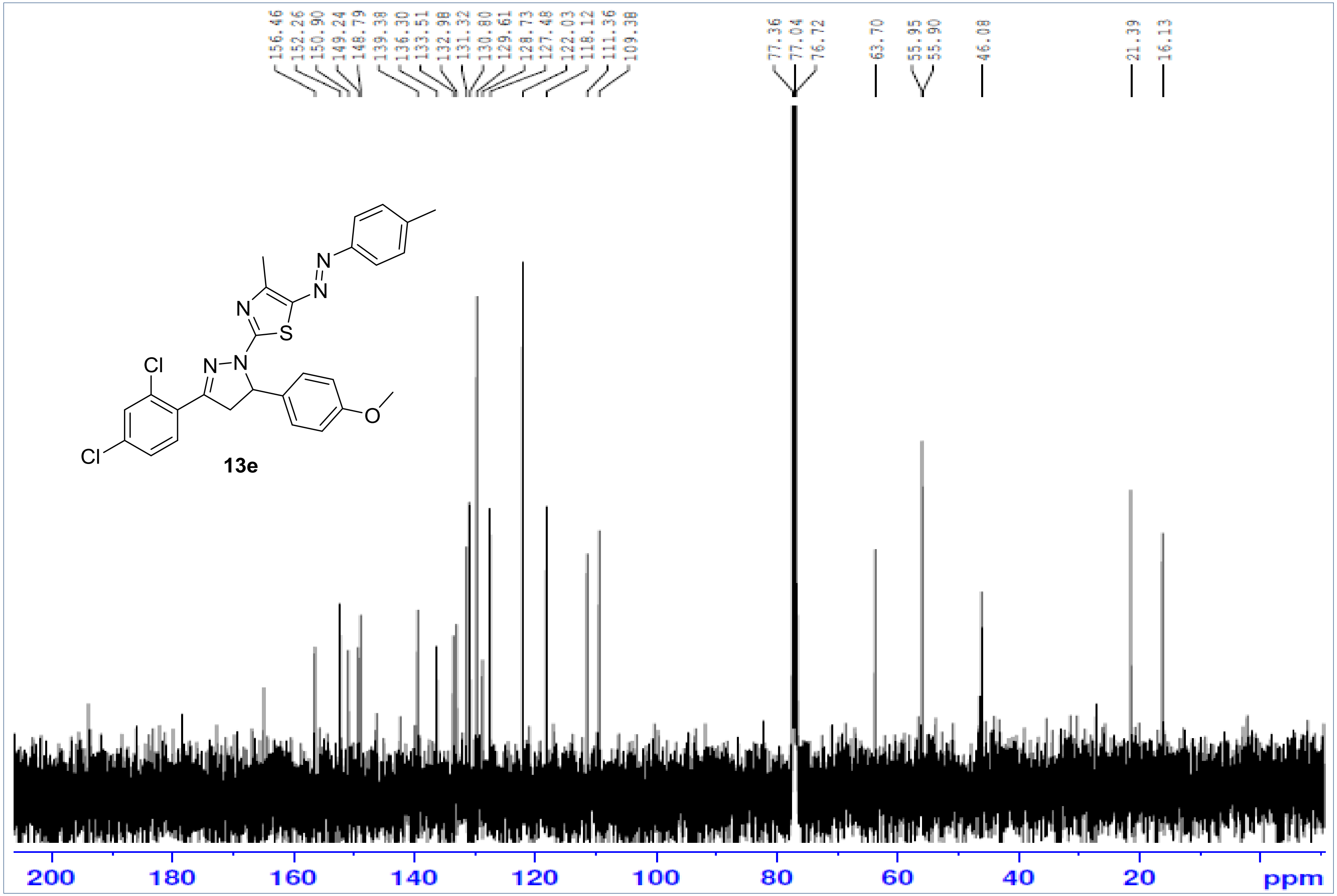

Appendix (28): ^{13}C NMR spectrum of compound **13e** (C*D*Cl$_3$, 100 MHz).

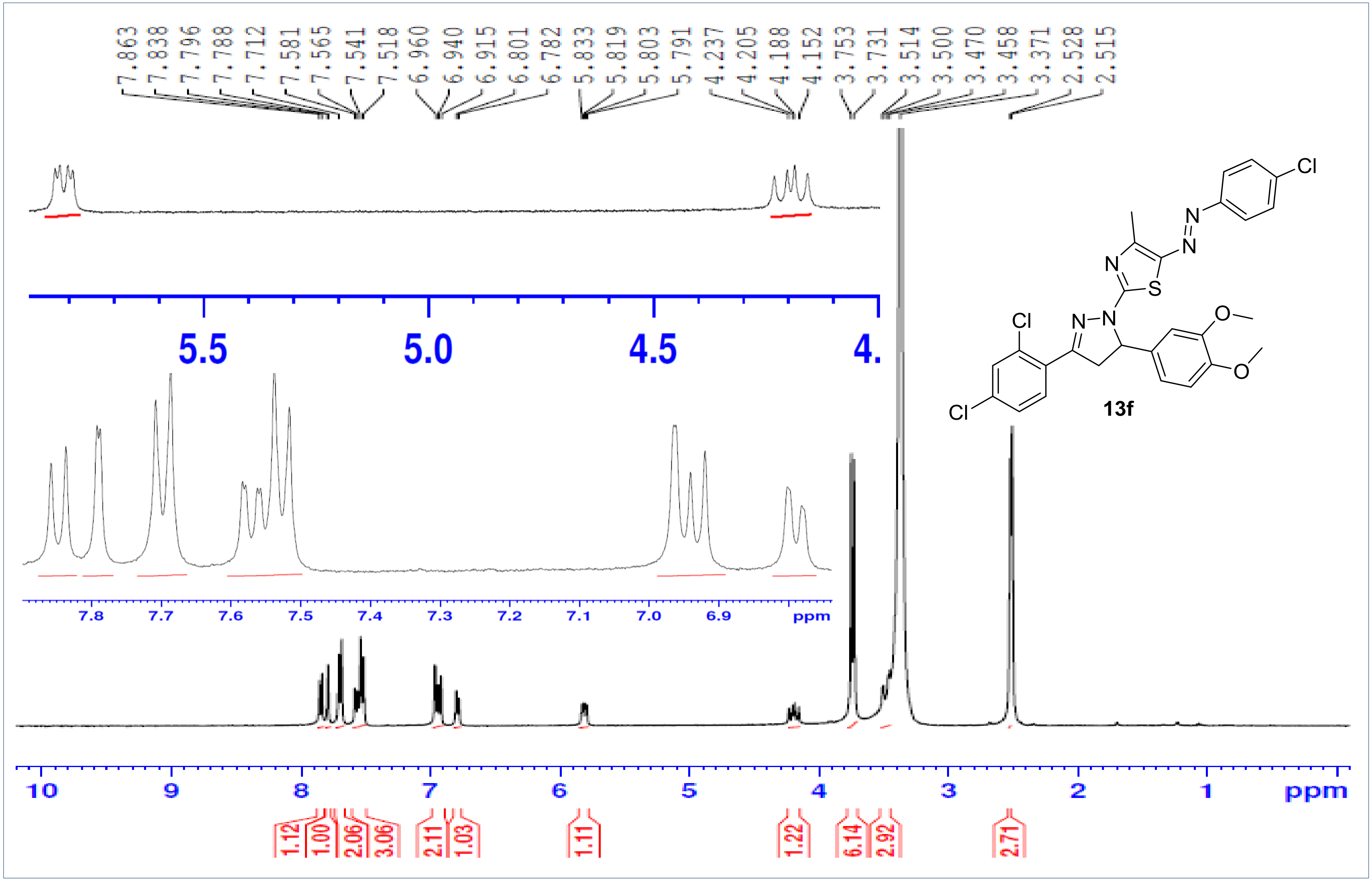

Appendix (29): ^{1}H NMR spectrum of compound **13f** (DMSO-d_6, 400 MHz).
